# A universal programmable Gaussian boson sampler for drug discovery

**DOI:** 10.1038/s43588-023-00526-y

**Published:** 2023-10-12

**Authors:** Shang Yu, Zhi-Peng Zhong, Yuhua Fang, Raj B. Patel, Qing-Peng Li, Wei Liu, Zhenghao Li, Liang Xu, Steven Sagona-Stophel, Ewan Mer, Sarah E. Thomas, Yu Meng, Zhi-Peng Li, Yuan-Ze Yang, Zhao-An Wang, Nai-Jie Guo, Wen-Hao Zhang, Geoffrey K. Tranmer, Ying Dong, Yi-Tao Wang, Jian-Shun Tang, Chuan-Feng Li, Ian A. Walmsley, Guang-Can Guo

**Affiliations:** 1 Research Center for Quantum Sensing, Zhejiang Lab, Hangzhou, People’s Republic of China; 2 Quantum Optics and Laser Science, Blackett Laboratory, Imperial College London, London, UK; 3 CAS Key Laboratory of Quantum Information, University of Science and Technology of China, Hefei, China; 4 CAS Center For Excellence in Quantum Information and Quantum Physics, University of Science and Technology of China, Hefei, China; 5 College of Pharmacy, Rady Faculty of Health Sciences, University of Manitoba, Winnipeg, Manitoba Canada; 6 Hefei National Laboratory, University of Science and Technology of China, Hefei, China

**Keywords:** Quantum optics, Quantum physics, Drug discovery

## Abstract

Gaussian boson sampling (GBS) has the potential to solve complex graph problems, such as clique finding, which is relevant to drug discovery tasks. However, realizing the full benefits of quantum enhancements requires large-scale quantum hardware with universal programmability. Here we have developed a time-bin-encoded GBS photonic quantum processor that is universal, programmable and software-scalable. Our processor features freely adjustable squeezing parameters and can implement arbitrary unitary operations with a programmable interferometer. Leveraging our processor, we successfully executed clique finding on a 32-node graph, achieving approximately twice the success probability compared to classical sampling. As proof of concept, we implemented a versatile quantum drug discovery platform using this GBS processor, enabling molecular docking and RNA-folding prediction tasks. Our work achieves GBS circuitry with its universal and programmable architecture, advancing GBS toward use in real-world applications.

## Abstract (web summary)

This study develops a programmable quantum processor, named Abacus, and applies it to Gaussian boson sampling tasks targeting drug discovery challenges.

## Main

Quantum computing technology has developed rapidly in recent years^1–7^, and an exponential ‘speed-up’ compared to classical methods has been experimentally demonstrated for certain algorithms^4,5,7–9^. Quantum sampling tasks, like boson sampling^10–12^, have proven to be challenging to solve within a reasonable time frame on classical computers, but can be implemented and solved efficiently on photonic processors^1,13^. As a variant of boson sampling, Gaussian boson sampling (GBS)^14^ uses squeezed light to encode and carry the input states, making the method easier to scale. The method shows great capacity to demonstrate quantum advantage in optical systems^5,7^.

The prospect of achieving quantum advantage has motivated the discovery of several real-world applications, such as dense graph searching^15,16^, molecular vibronic spectra calculations^6,17^ and molecular docking^18^. For these tasks, a GBS device should be programmable and scalable to a large number of modes^5,6^; however, achieving such capability is a challenging task^16^ due to the experimental complexity involved in preparing the large number of individually addressable input states and phase-shifters necessary to achieve universal programmability^5,6^.

Time-bin encoding of Gaussian states is an effective means of achieving scale and programmability^7,16,19–21^. First, it is resource efficient because only one squeezed source and one detector are required^16^. Second, time-bin operation provides phase stability and exhibits losses comparable with other approaches^22^. Time-bin interferometers show flexibility in reconfiguration because they can realize arbitrary-dimension linear transformations with the same setup. Recently, quantum computational advantage has been demonstrated with a programmable time-bin-encoded GBS machine^7^, though in that research, universality was sacrificed to avoid the accumulation of loss.

This prompts us to consider a universal and programmable time-bin GBS machine that can fulfill various practical tasks. The GBS algorithm can potentially be applied to many important problems and enhance their solution, for example, the complete subgraph (clique) finding task^23,24^. Some structure-based drug design methods, like molecular docking or protein folding prediction, can be interpreted as equivalent to the problem of finding the maximum weighted clique in their corresponding graph models^18,25,26^. Hence, a universal, programmable, GBS machine equipped with freely adjustable squeezers and interferometer can be utilized for these tasks and extend the range of practical applications based on graph theory. Inspired by this prospect, in this work we built a scalable, universal and programmable time-bin GBS machine, making a stride toward using GBS in drug discovery applications.

## Results

### Programmable GBS machine and sampling results

The GBS machine, named ‘Abacus’, comprises four primary components: a tunable squeezed-state source for precise light state control^27–30^; a quantum processing unit based on the architecture of Clements et al.^31^ that ensures stable, programmable quantum information processing^32^; a quantum sequential access memory with a 180-meter optical fiber designed for efficient data storage and cyclic operations; and a detection module featuring superconducting nanowire single-photon detectors (SNSPDs) for collision-free photon measurements. Further details are provided in the Methods section ‘Details about the programmable GBS machine’, as well as in Supplementary Information Sections 1 and 2.

**Fig. 1:**
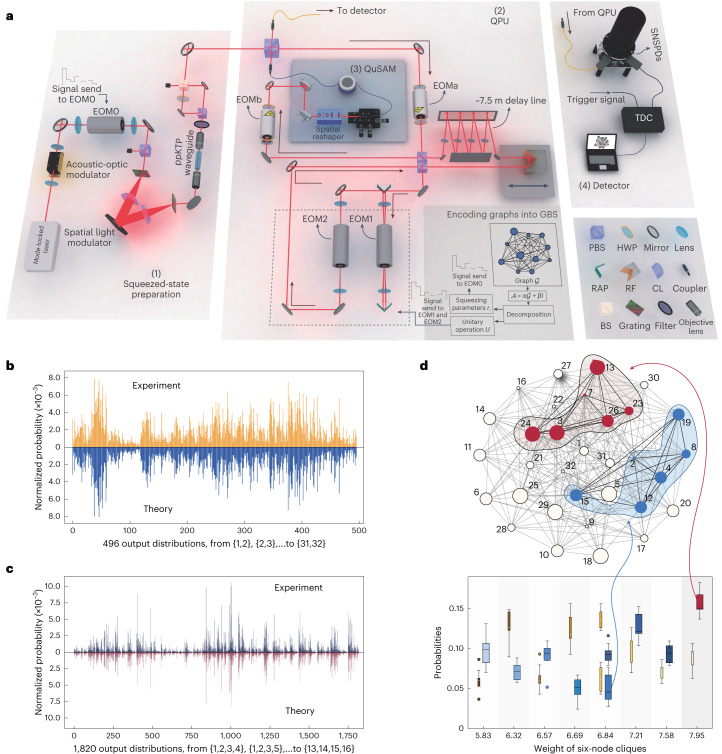
Implementation, verification and application of Abacus. **a**, The GBS machine consists of four main parts: (1) squeezed-state preparation, (2) QPU, (3) QuSAM and (4) detection. The red lines represent the squeezed-light signal, while the dark red arrows indicate the direction of the signal light within the QPU loop. For clarity, the control system, which includes three arbitrary waveform generators and a control computer, is not shown. The abbreviations indicate PBS, half-wave plate (HWP), right-angle prism mirrors (RAP), roof prism mirror (RF), cylindrical lens (CL), beam splitter (BS) and time-to-digital converter (TDC). **b**, GBS results, probability distribution of all 496 two-photon detection events in a 32-mode experiment. The horizontal axis numbering represents a methodical ordering by (*i*,*j*) from 0 to $C_{2}^{32}-1$ $({C^n}_k = n!/(k!(n - k)!))$, corresponding to the detection of a photon in output mode (*i*,*j*). **c**, GBS results, probability distribution of all 1,820 four-photon detection events in a 16-mode experiment. The horizontal axis numbering represents a methodical ordering by (*i*,*j*,*k*,*l*) from 0 to $C_{4}^{16}-1$, corresponding to the detection of a photon in output mode (*i*,*j*,*k*,*l*). **d**, Finding the maximum weighted clique in a 32-node graph. The probabilities of the six-node cliques in the graph ${{{{\mathcal{G}}}}}^{32}$ are shown at the bottom with box-and-whisker plots. The corresponding graph is shown above; the labels beside the nodes denote the corresponding order and the weight of the nodes is represented by their size. The statistics are calculated from ten individual experiments, each with around 300 samples. The wide blue boxes of varying shades, and the red box, symbolize the GBS experimental results. The lower and upper limits of the ‘whisker’ denote the minimum and maximum values (excluding outliers), respectively; and the horizontal black (or white) lines inside the boxes indicate the median value. The shaded regions denote the cliques identified through GBS results, with the red-shaded regions representing the maximum weighted cliques. The classical uniform sampling results are portrayed by slim yellow boxes of varying shades. In a few cases, outlier data are displayed as circles that match the color of the corresponding box. The diagram shows that the GBS machine can identify the maximum weighted clique with a higher success rate. Source data

As illustrated in Fig. 1a, this time-bin-encoded GBS machine enables us to expand the number of modes arbitrarily and freely set the required squeezing parameters, denoted as *r*_*i*_, and linear transformation matrix for the tasks with a series of electro-optic modulators (EOMs). Thus, this universal and programmable architecture supports the running of arbitrary GBS circuits on this machine. As a concrete example, benefitting from these features, the adjacency matrix ${\mathcal{A}}$ of a graph ${{{\mathcal{G}}}}$ can be encoded into this GBS machine by decomposing ${{{\mathcal{L}}}}({\mathcal{A}})$ (Laplacian of the adjacency matrix of graph ${{{\mathcal{G}}}}$) after a suitable re-scaling, as shown in the inset of Fig. 1a.

The validation of Abacus is demonstrated by the sampling results from running two random GBS circuits with different dimensions. The normalized photon sampling distribution probabilities are shown in Fig. 1b,c. In Fig. 1b, a 32-mode random interferometer is chosen, and only four squeezers are turned on, with *r*_1−3,32_ = 2.23. The statistical results of all two-photon detection events are plotted, and the total variation distance between experimental and theoretical results is 0.054. Similarly, the four-photon distribution pattern is shown in Fig. 1c, conducted on a 16-mode GBS with all 16 squeezers turned on and *r*_max_ = 1.8 (here, the total variation distance is 0.175). We also use the modified likelihood ratio test introduced in ref. ^33^ to exclude the thermal state and distinguishable photon hypotheses; these details can be found in Supplementary Information Section 2G. These results show that Abacus can perform the sampling tasks with high fidelity.

### Finding the maximum weighted clique with GBS

Not only can GBS be used to demonstrate quantum advantage in the laboratory^5,7^ as a near-term specific-function quantum computer, it can also be used in solving certain problems in real-world applications. Here, we use Abacus to solve max clique decision problems, which are NP-hard (non-deterministic polynomial time) problems in graph theory and play a crucial role in many applications^25^.

Clique refers to all the maximal complete subgraphs in a graph ${{{\mathcal{G}}}}$; the clique-finding problem has a complexity which scales exponentially with the number of nodes. Here, we use Abacus to find the maximum weighted clique in a graph. A 32-node weighted graph ${{{{\mathcal{G}}}}}^{32}$ is artificially constructed here (details are shown in Supplementary Information Sections 3 and 4), and the essential step is encoding ${{{{\mathcal{G}}}}}^{32}$ onto our GBS machine. Using the method introduced in refs. ^18,24^, we perform Takagi–Autonne decomposition to the Laplacian of graph ${{{\mathcal{G}}}}^{32}$, with appropriate re-scaling, and obtain the unitary operation *U* and the squeezing parameters *r*_*i*_, which are required to be programmed on the GBS (Methods). Then, we control the acoustic-optical modulator chopper with an arbitrary waveform generator to pump the periodically polled potassium titanyl phosphate (ppKTP) waveguide with 32 sequential pulses. EOM0 is used to adjust *r*_*i*_ for each time bin, and *U*, the unitary operation, is achieved by adjusting the input voltages of EOM1 and EOM2 in the time-bin interferometer. After mapping ${{{{\mathcal{G}}}}}^{32}$ onto Abacus, around 300 five (or more)-photon sampling results are collected in each experiment. Using these sampling data, we can find the cliques with nodes corresponding to the 30-time postprocessed sampling results (see details in Methods). Figure 1d displays all six-node cliques and their corresponding probabilities. The maximum weighted clique stands out as the most probable among them. In comparison to classical sampling with the same postprocessing iterations, GBS demonstrates a substantially higher probability of successfully finding the maximum weighted clique, approximately twice as much. This indicates that GBS can perform the clique-finding task with high efficiency^18^.

### Molecular docking with GBS

If the graph is constructed according to an actual system occupying the network structure, the clique-finding task then could be utilized to find the optimal subset corresponding to the maximum weighted clique. Recent research shows that the information of the best docking orientation of the protein–ligand complex can be predicted by the maximum weighted clique of a corresponding binding interaction graph (BIG), which is a weighted graph constructed based on docking modes between ligand and receptor^18^. In the BIG, the weighted nodes represent the interacting pharmacophore pairs weighted by potential, and the edges represent the compatible contacts (Methods). By encoding the BIG on Abacus, we can solve molecular docking problems by finding the maximum weighted clique in the BIG^18,34^, as we demonstrated in Fig. 1d.

**Fig. 2:**
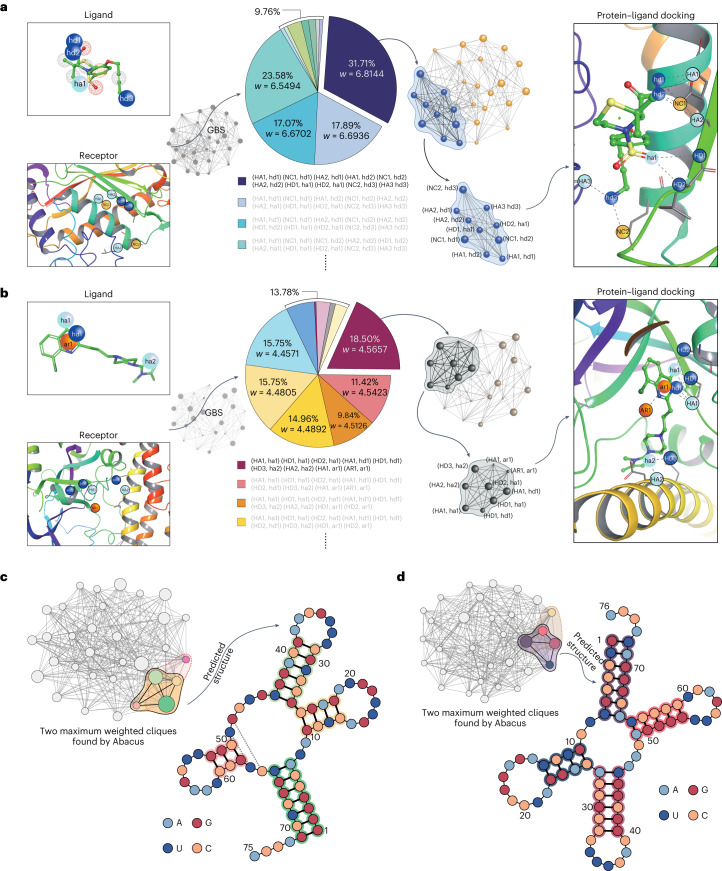
Molecular docking and RNA-folding prediction results obtained with Abacus. **a**, The docking pair of PARP-CQ (see text). Abacus is encoded with a 24-node BIG and finds the maximum weighted clique, using 347 sample data and 100-iteration local searches. **b**, Another 28-node BIG constructed by the complex of TACE-TS (see text), using 254 sample data and 10 iterations. In **a** and **b** the colored spheres denote the pharmacophore points we considered in the experiment (cyan: hydrogen-bond acceptor, HA, ha; blue: hydrogen-bond donor, HD, hd; yellow: negative charge, NC; orange: aromatic, AR, ar; and we use capital letters to represent pharmacophore points in the protein and lowercase letters to represent pharmacophore points in the ligand.) The sphere meshes in the ligands are other possible pharmacophore points but are not considered in our experiments. The shaded region in panels **a** and **b** denotes the maximum weighted cliques, as determined by the experimental results. All the cliques found from GBS experiments are shown in the pie charts; the maximum weighted cliques in both cases have a major proportion in the experimental results obtained from QIVS. More details are found in Supplementary Information Sections 5 and 6. **c**,**d**, The GBS-based RNA-folding prediction results for two RNA sequences (in **c**, accession no. AH003339; in **d**, accession no. AB041850). The max weighted cliques are highlighted in colored regions; they represent the most likely folding structures. The four colored circles (A, G, U, C) in the RNA structure represent the four different bases and the gray dashed lines represent the false negative base-pair matching. The colors of nodes within the clique, outlined by solid black lines, correspond to the predicted stems (base-pairs with matching shadow colors) in the RNA folding structure. For example, in **c**, the yellow node corresponds to the predicted stem with base-pairs 10–25, 11–24, 12–23 and 13–22. The cliques encircled by dashed pink (**c**) and yellow lines (**d**) represent other folding structures, though they are less accurate. More details are shown in Supplementary Information Section 7. Source data

To demonstrate the capability of GBS in solving molecular docking problems, we build a quantum inverse virtual screening (QIVS) platform based on Abacus and use two pairs of protein–ligand complexes with different drug properties to demonstrate the ability of QIVS in drug design and verified the practicability of the platform. In the first case (Fig. 2a), a 28-node BIG ${{{{\mathcal{G}}}}}_{_{\,{{\mbox{PARP-CQ}}}}\,}^{28}$ is constructed based on the Poly (ADP-Ribose) polymerase-1 (PARP) and an 8-chloroquinazolinone-based inhibitor (PARP-CQ), which is a promising candidate for anticancer drugs^35–37^ and for some central nervous system diseases such as Parkinson’s and Alzheimer’s diseases^38,39^. The structures of ligand and protein and their BIG are shown in Fig. 2a. By encoding ${{{{\mathcal{G}}}}}_{\,{{\mbox{PARP-CQ}}}\,}^{28}$ onto Abacus, we collect the sampling results and find the associated cliques with postprocessing (that is, shrink and local search)^18^. The pie chart in Fig. 2a shows all the cliques we find with GBS experiments, where each sector corresponds to one the various cliques with corresponding weights. The maximum weighted clique (with seven nodes and weight = 6.8144) occupies the major proportion. This demonstrates we can use Abacus to find the best binding pose (Fig. 2a, right side) of this complex with a high success rate.

In the second case (Fig. 2b), we use the complex of tumor necrosis factor (TNF) converting enzyme (TACE) and thiomorpholine sulfonamide hydroxamate inhibitor (TACE-TS)^40^, which are involved in inflammatory diseases^41^. Aromatic pharmacophore points are included and, in order to increase the accuracy of the docking results, an improved algorithm is used. Considering the fact that the interaction strength between various pharmacophore points may create some behavior differences, the variable distance is used to compare the distance between different points when we construct the 24-node BIG ${{{{\mathcal{G}}}}}_{\,{{\mbox{TACE-TS}}}\,}^{24}$. GBS experiments then are performed by programming the circuit with another set of polarization rotation (*θ*_*i*_), phase shift (*φ*_*i*_) and *r*_*i*_. We find 11 cliques in the sampling results; and six of them (maximum size *N* = 9) appear with relatively high probabilities. The protein–ligand docking position suggested by the maximum weighted clique (with weight (*w*) = 4.5657) is shown on the right in Fig. 2b. Compared with the method used by Banchi et al.^18^, the improved method we proposed here obtains a more accurate binding pose result; the detailed comparison analysis is shown in the Supplementary Information Sections 5 and 6.

Although there is relatively high loss in the experiment, the maximum weighted clique can still be found with a high success probability through postprocessing, which is robust with regard to noise^18^. The above results, predicted by GBS experiments, agree well with the outcomes obtained from the corresponding co-crystal structures, which can be found by reviewing complex structures within a certain distance (*τ*) to each other^40,42,43^, through their entries in the Protein Data Bank (PDB) for PARP1 (PDB entry no. 7ONR) and TACE (PDB entry no. 2A8H).

### GBS for RNA-folding prediction

The molecular docking process relies heavily on the protein structure, and the fact is that many pathogenic proteins associated with human diseases cannot be targeted by conventional small-molecule drugs or biomacromolecules^44^. In recent years, nucleic acid drugs have received attention in the pharmaceutical field as a potential solution to overcome the limitations of existing target drugs and to treat previously untargeted diseases. Predicting RNA structures has become an important task in discovering these nucleic acid drugs, as it can aid in identifying potential drug targets and predicting small-molecule-drugs’ interactions with RNA molecules^45^. However, predicting RNA structures by calculation has proven difficult, as only a few RNA structures are known. Nevertheless, exciting work in protein and RNA structure prediction has emerged recently, with artificial intelligence technology being particularly prominent^45,46^. Quantum computational technology also has great potential to solve this folding prediction task^47,48^. However, no solution to this problem has yet been experimentally demonstrated on devices that can exhibit quantum computational advantages (such as GBS devices) due to their programmability limitations.

Using our universal programmable GBS device Abacus, we use a method, inspired by Tang et al.^26^, for predicting RNA sequence folding. This approach involves modeling the RNA sequence as a weighted full stem graph (WFSG) and then encoding it into our universal programmable GBS device. The WFSG captures all possible folding information of the RNA sequence, where each node represents a possible stem in the sequence, and the edges indicate the co-existence between them^26^. The weight of each node corresponds to the length of the stem it represents. Then, the RNA-folding prediction can be obtained by finding the maximum weighted cliques in WFSG^26^. To demonstrate the effectiveness of our GBS machine in solving this problem, we conducted two experiments with different RNA fragments on Abacus; the results are shown in Fig. 2c,d.

In the first example, we predicted the secondary structure of an RNA sequence (accession no.: AH003339) by encoding the corresponding 32-node WFSG into Abacus. We found two maximum weighted cliques, and the Matthews correlation coefficient (MCC) of the best one (shown in light orange shadow in Fig. 2c) reached 0.953, which outperforms FOLD^49^ (best case) and RNAProbing^50^, with MCC values of only 0.864 and 0.934, respectively. In the second experiment, we use the RNA sequence of the organism Alanine (accession no.: AB041850) and encoded its corresponding WFSG, which had 31 nodes, into Abacus by modifying the control program. The best prediction with MCC = 1.00 among the two results is shown in Fig. 2d, and it is more accurate than those obtained by other methods, with FOLD achieving MCC = 0.870 and RNAProbing achieving MCC = 0.914. Details of the true reference folding and other information are provided in Supplementary Information Section 7.

## Discussion

The scalability and programmability of our universal GBS machine enable its utilization in real-world applications, as demonstrated in this work. The ability to program arbitrary graphs demonstrates that drug discovery tasks, such as molecular docking or RNA-folding prediction, can be performed efficiently by a purpose-built quantum computer. However, unequivocal quantum computational advantage^5,7^ has not been realized in our experiments due to photon loss. Although the question of whether GBS can outperform improved classical algorithms or quantum-inspired algorithms remains open^51^, and the potential for GBS to demonstrate computational advantages also relies on the properties of the encoded graph, we remain optimistic about scaling Abacus to several hundred modes using the ‘multicore encoding’ and ‘distributed computing’ methods. This scalability holds the potential to unlock quantum advantages in some specific real-world applications. Additionally, it is crucial to consider practical applications that encompass more complex protein structures, larger pharmacophore points and longer RNA sequences, which also necessitate the use of such a large-scale GBS machine. For a comprehensive discussion on scaling our GBS machine by minimizing loss and utilizing the multicore encoding and distributed computing methods, refer to Supplementary Information Section 8. Apart from offering programmability and universality, this work presents a promising hardware solution for the near-term industrial implementation of quantum computing in the biopharmaceutical industry. It also paves the way for diverse real-world applications in the future.

## Methods

### Details about the programmable GBS machine

The GBS machine shown in Fig. 1a, which we named Abacus, can be divided into four main parts: (1) Tunable squeezed-state source. The pump light from a mode-locked pulsed laser (80 MHz, 773 nm, ~150 fs) is reduced in repetition rate to 40 MHz by an acoustic-optic modulator. The electro-optic modulator (EOM0) and polarization beam splitter (PBS) are used to adjust the pump energy of each pulse. This controls the squeezing degree (*r*_*i*_) of the squeezed vacuum states in each time bin. The spectral mode of the pump light is modulated by a spatial light modulator, two gratings and two cylindrical lenses (CLs). Then, spectrally uncorrelated two-mode squeezed light is generated by pumping a 10-mm-long ppKTP waveguide^27–29^. Following interference at a 50:50 beamsplitter, a series of individually addressable single-mode squeezed states can be efficiently prepared^30^. (2) Quantum processing unit (QPU). The single-mode squeezed states are then sent into a time-bin interferometer, which is programmed for a specific unitary operation. This is achieved according to Clements’ architecture^31^, which is realized by a group of Mach–Zehnder interferometers consisting of two high-speed optical switches EOMa and EOMb, a 7.5 m delay line (to combine or separate two adjacent time bins) and a linear transformation $$T(\theta ,\varphi )=\left(\begin{array}{cc}{e}^{i\varphi }\cos \theta &-\sin \theta \\ {e}^{i\varphi }\sin \theta &\cos \theta \\ \end{array}\right),$$ where *φ* represents the phase shift and *θ* denotes the polarization rotation, these are achieved by EOM1 and EOM2, respectively. Since the optical path before and after *T*(*θ*, *φ*) passes through the same low-loss free-space delay line, the phase stability of the setup is well guaranteed, and the non-uniform loss expected in the fiber-loop scheme^32^ is mitigated. (3) Quantum sequential access memory (QuSAM). In each loop of evolution, the quantum memory is achieved by a 180-meter-long optical fiber delay line. The QuSAM ensures that the last time bin has completed the operation in one cycle before the first time bin enters into the next cycle. With a 4f beam-shaper system, we can efficiently couple the light from free space into single-mode optical fiber and realize a low-loss time-bin memory (with total efficiency of ~94%) by reshaping the spatial mode of the beam. (4) Detection module. SNSPDs are used to detect the single-photon events, since our experiments are performed in the collision-free space. To avoid the issue of the SNSPD dead-time (≲50 ns) being longer than the time interval between two adjacent time bins (25 ns), we use another EOM to separate two adjacent time bins and use two SNSPDs for detection. The throughput of each round-trip in the system is approximately 82%. In the case of Fig. 1b, the average count rate of two-fold events is 45 counts per second, and in the case of Fig. 1c, the four-fold average count rate is 24 counts per second, which are calculated from 10^7^ samples in 10 min (the repetition rate of each individual sampling experiment is 20 kHz). More details are provided in Supplementary Information Section 2.

This GBS machine has two main advantages compared with previous works: First, universal operation is possible since both the squeezers and arbitrary unitary matrices can be programmed on the time-bin interferometer. This makes it suitable not only for molecular docking of various molecules but also for other applications. Our architecture also provides flexibility in scaling to many modes via the control software. Compared to previous work^5,7^, our GBS setup supports adjustments to all the parameters: *n* squeezing parameters *r*_*i*_ and *n*(*n* − 1)/2 parameters for an arbitrary *U*. Our time-bin-encoding GBS setup is resource-efficient for scaling up. Specifically, when the number of modes increases, we do not need to add more squeezed-light sources. Independent of the number of modes we required in experiments, two analog EOMs (that is, EOM1 and EOM2) assisted with two light-switch EOMs (that is, EOMa and EOMb) are sufficient to realize any linear transformation. A resource advantage is also exhibited in the detection. As we discussed in the main text and Supplementary Information Section 2E, two SNSPDs are enough for collecting the ~30-mode GBS samples.

Second, non-uniform loss in previous time-bin interferometer implementations appears across different time-bin modes, and this limits the ability to perform an arbitrary unitary operation^32^. In this setup, we use a free-space delay line with transmittance 0.995 to greatly reduce the non-uniform loss. Thus, the mitigated non-uniform loss and dispersion-free features in our setup can better exhibit universality. The time-bin-encoded GBS scheme is intrinsically phase stable^20^. As shown in Fig. 1a, since every time bin goes through the same path, the slow phase fluctuations (caused by mechanical vibrations, temperature drifts or other unpredictable environmental noise) can be neglected compared to the high sampling rate where a sample is obtained in 50 μs. The 7.5 m free-space delay line is isolated from the environment to ensure that the phase between two adjacent time bins can be stable for up to 5 min. This is enough for collecting 10^6^ samples within 1 min.

### Mapping a graph onto GBS

As for a loopless, undirected and vertex-weighted graph ${{{\mathcal{G}}}}$, the corresponding adjacency matrix ${{{\mathcal{A}}}}$ is a symmetric (0,1)-matrix, where the vertex weights are given by *ω*_*i*_, and the entries are ${\mathcal{A} _{ii}=\omega _i}$, ${{{{\mathcal{A}}}}}_{ij}=1$ if there is an edge between vertex *i* and *j* and ${{{{\mathcal{A}}}}}_{ij}=0$ if otherwise. After finding the adjacency matrix ${{{\mathcal{A}}}}$ of a graph, then the key step to encode the graph ${{{\mathcal{G}}}}$ onto GBS machine is connecting ${{{\mathcal{A}}}}$ with the sampling matrix *A* through some proper transformation (the inset in Fig. 1a provides an example possibility).

For a pure Gaussian state, the sampling matrix *A* can be written as *B* ⊕ B^*^; then the Hafnian Haf(*A*) can be expressed as Haf(*B* ⊕ B^*^) = ∣Haf(*B*)∣^2^. Thus, the output probability of obtaining the sampling pattern *s* can be expressed as

1 $$\,{{\mbox{Pr}}}(s)=\frac{| {{\mbox{Haf}}}({B}_{s}){| }^{2}}{{n}_{1}!{n}_{2}!\cdots {n}_{N}!\sqrt{{{\mbox{det}}}\,(\sigma +{\mathbb{I}}/2)}}.$$

An immediate idea is to replace *B* here with the adjacency matrix $\varOmega ({{{\mathcal{D}}}}-{{{\mathcal{A}}}})\varOmega$, where *Ω* is a diagonal matrix with elements $({\varOmega }_{ii}={\omega }_{i})$ and ${{{\mathcal{D}}}}$ is the degree matrix of ${{{\mathcal{A}}}}$ defined as ${{{{\mathcal{D}}}}}_{ii}={\sum }_{j}{{{{\mathcal{A}}}}}_{ij}$. However, this does not work, since the eigenvalues of *B* should be within the interval [0, 1), such that the covariance matrix of the pure Gaussian state is positive definite^24^. From an experimental point of view, this restriction is because the eigenvalues of *B* denote the brightness of squeezing sources in the experiment. We can decompose the symmetric matrix *B* as^52^

2 $$B=U{\oplus }_{i}^{N}\tanh ({r}_{i}){U}^{T},$$

where *U* is the unitary matrix applied in experiments and *r*_*i*_ denotes the squeezing parameters for the vacuum states, as shown in Supplementary Information Fig. 1a. Thus, the value of eigenvalues $\tanh ({r}_{i})$ should be between 0 and 1, and 1 cannot be reached because that would correspond to infinite brightness.

To satisfy this condition, we can rescale ${{{\mathcal{A}}}}$ by carefully choosing the parameters *c* and *α* when we add the weight of each vertex to the adjacency matrix:

3 $${{{{\mathcal{A}}}}}^{{\prime} }=\varOmega ({{{\mathcal{D}}}}-{{{\mathcal{A}}}})\varOmega ,$$

where *Ω* is a diagonal matrix with elements *Ω*_*ii*_ = *c*(1 + *αω*_*i*_). By choosing parameters *c* and *α* suitably, we can obtain an ${{{{\mathcal{A}}}}}^{{\prime} }$ with the correct spectrum. Additionally, we also can maximize the maximum input photon number in the experiment over the choice of *c* and *α*. The ${{{\mathcal{D}}}}$ matrix is introduced so that the ${{{{\mathcal{A}}}}}^{{\prime} }$ matrix is positive definite^18^. But when GBS is operated in the collision-free subspace, ${{{\mathcal{D}}}}$ will not affect the results of $\,{{\mbox{Haf}}}\,({{{{\mathcal{A}}}}}^{{\prime} })$ and, according to Banchi et al.^18^, $\,{{\mbox{Haf}}}\,({{{{\mathcal{A}}}}}^{{\prime} })=\det (\Omega )\,{{\mbox{Haf}}}\,({{{\mathcal{A}}}})$. More details can be found in Supplementary Information Section 3.

### Constructing the adjacency matrix of a BIG

Inverse virtual screening is a structure-based approach to find potential drug targets for a given drug or active small molecule by calculation. For known drugs, inverse virtual screening technology can do the drug repositioning and provide reference for the study of new drug effects and drug side effects^53^. For active small molecules, inverse virtual screening technology can predict their potential targets, identify their therapeutic potential in diseases and provide direction for the later transformation and mechanism research of active compounds^54,55^.

If the graph is constructed according to an actual system occupying the network structure, the clique-finding task then could be utilized to find the optimal subset corresponding to the maximum weighted clique. Recent research shows that the information of the best docking orientation of the protein–ligand complex can be predicted by the maximum weighted clique of a corresponding BIG^18^. In the docking scheme of bioactive molecules, the BIG is a weighted graph constructed based on docking modes between ligand and receptor. In the BIG, the weighted nodes represent the interacting pharmacophore pairs weighted by potential and the edges represent the compatible contacts.

The edge generation in a BIG is determined by comparing the distances between the pharmacophore points on the ligand (${D}_{{\mathrm{L}}}^{x-x}$) and binding (${D}_{{\mathrm{P}}}^{x-x}$) sites. This is illustrated in boxes 1 and 2 of Supplementary Information Fig. 21. Each possible contact is represented by a vertex in BIG, and the corresponding weight is determined by the contact potential (shown in Supplementary Information Fig. 23). A BIG with only weighted vertices is illustrated in box 3 in Supplementary Information Fig. 21.

While the geometric distances between two contacts should normally be approximately the same on both the ligand and the binding site, they can exhibit some degree of flexibility^18^. A pair of contacts, for example, (C, a) and (B, c), is considered a *τ*-flexible contact pair if the difference between the distances of the pharmacophore points on the ligand (corresponding to vertices ‘C’ and ‘B’) and the distances of the pharmacophore points on the binding site (corresponding to vertices ‘a’ and ‘c’) is within *τ* + 2*ε*, as depicted in Supplementary Information Fig. 21. The constants *τ* and *ε* describe the flexibility constant and interaction distance, respectively, and they determine which edges appear in the BIG, as shown in boxes 4 and 5 in Supplementary Information Fig. 21.

It should be noted that, in the work of Banchi et al.^18^, *τ* and *ε* are set as constants for simplicity, as discussed above. However, more accurate methods can account for the changeable flexibility of the ligand and receptor^18,56^. *τ* and *ε* are no longer set as constants and can vary according to the various pharmacophore points in the contacts.

By encoding the BIG on Abacus, we can solve molecular docking problems by finding the maximum weighted clique^18,34^ in the BIG, as we demonstrated, with results shown in Fig. 2.

To better demonstrate the capability of Abacus in solving molecular docking problems, we build a QIVS platform, as shown in Supplementary Information Fig. 22. Inverse virtual screening (IVS) is a technology for finding potential drug targets for a given drug or small active molecule by calculations, and it has been applied to identifying targets, research on side effects and drug repurposing^57,58^. In such a computer-aided drug design method as IVS, a large amount of computational resources are usually required, since a huge number of proteins in the database need to be screened by this docking program to identify potential targets for a given ligand, with a run-time that scales with the size of the ligands and receptors.

Unlike the classical IVS, we replace the traditional molecular docking process with GBS and achieve a more efficient and accurate QIVS. According to the selected ligand and potential proteins, a corresponding BIG, ${{{{\mathcal{G}}}}}_{{{\mbox{BIG}}}}$, is constructed (shown in Supplementary Information Fig. 22) and is then encoded into the GBS machine. The best binding pose can be determined by finding the maximum weighted clique of this graph ${{{{\mathcal{G}}}}}_{{{\mbox{BIG}}}}$ (ref. ^18^), a task for which our programmable Abacus is well suited. More details can be found in Supplementary Information Section 5.

### Postprocessing method with ‘Shrinking’ and ‘Local search’

The presence of various types of noise in the experiments affects the probability of the maximum weighted clique obtained from the raw experimental data. In some cases, the subgraph obtained may not even be a clique. Certain types of noise are unavoidable in experiments (for example, photon loss), in which case we can use the raw experimental data as a seed which can be input to a postprocessing algorithm to generate cliques at a high-rate. The postprocessing method introduced in Banchi et al.^18^ is very useful for this purpose. We briefly review the postprocessing method and discuss how we use it in our experiments.

We use ‘Greedy Shrinking’ to ensure the subgraph obtained from the raw GBS data is a clique by removing the nodes based on the degree and weight of the nodes until it forms a clique. To obtain the maximum weighted clique, which usually occurs with a larger number of nodes, we perform an expansion with a ‘Local search’. This expands the clique by adding neighboring nodes within several iterations of the algorithm to generate the largest clique. This is represented as the ‘Postprocessing’ module in Supplementary Information Fig. 22. Strawberry Fields^59^ is used to perform the postprocessing. Further details can be found in the work of Banchi et al.^18^

### Further scaling by reducing loss

In our experiment, simultaneously achieving universality and programmability comes at the cost of loss, which increases with the circuit depth or, more specifically, the number of cycles. This relatively large loss exists in our GBS machine prohibits demonstration of quantum computational advantage. Although we mainly focus on the mapping of GBS to real-world applications in this work, with further developments toward low-loss optical components, realization of quantum advantage should be possible in the future.

Loss in Abacus can be reduced by various methods. Loss in the experiment mainly comes from (1) the coupling loss from the ppKTP waveguide to single-mode fiber, (2) insertion loss caused by EOMs, (3) the limited coupling efficiency from free space to QuSAM and (4) the limited detection efficiency of SNSPDs. Particularly, for the EOM insertion loss (2) and the limited QuSAM coupling efficiency (3), due to the characteristics of our free-space loop architecture, the total loss will increase exponentially with the loss inside the loop. Therefore, when the number of modes is large, small improvements to these sources of loss will greatly improve the overall loss.

First, for the coupling loss from the ppKTP waveguide to single-mode fiber, mode shaping techniques may be applied to match the spatial mode of light from the ppKTP waveguide to that of the fiber, potentially improving the coupling efficiency to greater than 0.9 (ref. ^60^). Second, the insertion loss of EOMs or other optical elements is unavoidable. However, an EOM with a shorter and lower loss crystal—driven by a higher-gain amplifier—could be used. Combining the actions of EOM1 and EOM2 into an integrated EOM operation will further reduce the loss experienced upon reflection and absorption at the end faces. Transmission has been shown to reach higher than 0.99 after optimizing the EOM^7^. Third, the coupling efficiency from free space to QuSAM can be improved up to ~0.97 by using a 4f or 8f imaging systems through spherical lenses and graded-index lens fiber couplers (see Madsen et al.^7^). In addition, a Herriott long-distance delay line can be used as a quantum memory for minimizing the loss^61^. Finally, the detection efficiency can be improved with the latest generation of nanowire detectors with detection efficiencies at 1,550 nm of up to 0.95. Through these methods to minimize the loss, the single-loop transmission can potentially reach ~90%. Thus, our GBS setup can be extended to at least 60 modes (for example, a 60-mode GBS experiment will have total transmission efficiency of ~0.12%, found from the product of the several involved transmission efficiencies, as 0.90^61^ (loop efficiency, to the loop-count power) × 0.9 (coupling efficiency from ppKTP to fiber) × 0.944 (filter after ppKTP induce) × 0.93 (coupling efficiency from QPU to fiber) × 0.973 (transmission of demultiplexer) × 0.95 (detection efficiency of SNSPDs).

### Implement displacement and photon-number resolving detection in time-domain GBS

Displacement operation *D*(*α*) is entirely feasible to include in Abacus. To achieve this, the photon source module needs to be rebuilt, which involves incorporating an optical parametric oscillator after the mode-locked laser (Chameleon) to generate a 1,550 nm laser. This laser is subsequently split into two separate paths. In one path, the light serves as the pump for generating squeezed states. In the other path, the light is utilized as a coherent state to achieve the displacement operation. The addition of a delay line ensures that the coherent state and squeezed state reach the beamsplitter simultaneously, enabling optimal interference at the output. Two EOMs facilitate the programmability of the amplitude and phase of the displacement operation. For more detailed information, please refer to Supplementary Information Section 2H.

Our time-domain GBS machine Abacus can also implement photon-number resolving detection with a transition edge sensor (TES). The TES initially needs to be cooled below its transition temperature of approximately 100 mK and then heated back to its transition temperature by applying a bias current^62^. To maintain the TES at this temperature, it should be operated inside a dilution refrigerator. After a photon absorption event, it takes approximately 5 μs for the TES to return to its original temperature. Therefore, the repetition rate of TES detectors is usually limited to around 100–300 kHz. This necessitates the installation of a demultiplexer in our time-domain GBS setup. To address this limitation for a time-domain GBS machine, a demultiplexer needs to be installed. By employing a loop structure, it is straightforward to implement a nine-channel demultiplexer using a single EOM. The EOM enables us to manipulate the polarization of photons in each time bin, thereby determining whether they exit the system through PBSs or undergo internal reflection and remain within the loop. The design corresponding to this approach is depicted in Supplementary Information Fig. 15, and further detailed information can be found in Supplementary Information Section 2I.

### Pharmacophore points selection

The selection of PARP–PARPi pharmacophore points—where PARPi refers to PARP inhibitors, including the PARP-CQ we used as shown in Fig. 2a—is according to previous research focused on PARP–PARPi relationships^63,64^. These articles have demonstrated important amino acid residues from the protein and functional groups from the inhibitor that will influence the efficacy of the protein–ligand interactions. We choose some of them for the GBS machine due to the size of the experiment.

The selection of pharmacophore points in this work is typically based on prior knowledge from experimental studies, structural analysis or computational modeling of similar PARP–PARPi complexes. The selection of pharmacophore points, such as hydrogen-bond acceptors and donors, negative charges, pi–pi interaction and aromatic ring in the PARP–PARPi complex is based on their known importance in the interaction between the protein, PARP and the ligand. These pharmacophore characteristics play a crucial role in the binding affinity and specificity of ligands to protein targets.

Hydrogen-bond interactions are important for stabilizing the protein–ligand complex. Such an interaction occurs between the hydrogen atom of the ligand and a hydrogen-bond acceptor or donor group on the protein. These pi–pi interactions contribute to the overall binding strength and specificity by forming specific and directional interactions; they involve the stacking of aromatic rings in the ligand and the protein. These interactions are driven by the pi electrons present in the aromatic systems and contribute to the stability of the complex. Such pi–pi interactions are often found in protein–ligand interactions and can enhance binding affinity. Some articles have reviewed pharmacophores in a PARP inhibitor, and in those studies the nicotinamide component is considered as a hydrogen-bond donor and acceptor, as well as being a part of pi–pi interaction with the tyrosine residue^63^.

Aromatic rings are frequently present in ligands and proteins and can participate in various types of interactions, including pi–pi stacking, hydrophobic interactions and van der Waals interactions. Aromatic rings provide a hydrophobic surface that can interact with complementary hydrophobic regions in the protein, contributing to the overall binding affinity. The aromatic ring at the tail of the compound is also critical, and we take it as a pharmacophore as well^42^.

Negative charges, represented by negatively charged atoms or functional groups, also play a major role in the PARP–PARPi complex. Such a negative charge can interact with positively charged residues on the protein, like arginine or lysine, through electrostatic interactions, and these interactions can also contribute to the stability of the complex and enhance ligand binding.

### Classical combinatorial optimization methods

In Supplementary Information Section 9, we discuss more details about the classical or quantum-inspired approaches like genetic algorithms, quantum approximate optimization algorithm, quantum annealing and digital annealer, which also can be used to realize clique finding and other similar tasks.

### Supplementary information

Supplementary Information Supplementary Figs. 1–46 and Discussion Sections 1–9.

Supplementary Data 1 Photon-number distribution of 32-mode GBS experiments.

Supplementary Data 2 Adjacency matrix of the graph of crystal structure of catalytic domain of TACE with a thiomorpholine sulfonamide hydroxamate inhibitor.

Supplementary Data 3 Adjacency matrix of the graph of PARP1 catalytic domain in complex with an 8-chloroquinazolinone-based inhibitor.

Supplementary Data 4 Adjacency matrix of tRNA fragment (accession no.: AH003339) used in experiments.

Supplementary Data 5 Adjacency matrix of tRNA fragment (accession no.: AB041850) used in experiments.

### Source data

Source Data Fig. 1 Experimental source data.

Source Data Fig. 2 Experimental source data and program for analyzing.

## Data Availability

Source data are provided with this paper. The experimental data used in this paper are also publicly available in a Zenodo repository at 10.5281/zenodo.8306628 with a citable release at ref. ^65^.

## Code availability

The codes used to generate the corresponding adjacency matrices in Fig. 2a–d, analyze experimental data and implement the Bron–Kerbosch and Maximum Clique algorithms for result verification are available at 10.5281/zenodo.8284043 with a citable release at ref. ^66^.
