# Supplementary material for: A universal programmable Gaussian boson sampler for drug discovery
Source: Nat Comput Sci. 2023 Oct 12;3(10):839–48. doi: 10.1038/s43588-023-00526-y (PMC10768638; doi:10.1038/s43588-023-00526-y)
Supplement: Supplementary file 1 — Supplementary Figs. 1–46 and Discussion Sections 1–9. [file 43588_2023_526_MOESM1_ESM.pdf]

nature computational science

Brief Communication https://doi.org/10.1038/s43588-023-00526-y

A universal programmable Gaussian boson sampler for drug discovery

In the format provided by the authors and unedited

CONTENTS

I. Brief review of Gaussian boson sampling 2

II. Experimental details 3
A. Squeezed light sources 3
B. EOMs and the linear transformation 5
C. Control system 7
D. Quantum sequential memory with fibre delay line 8
E. Detection system 8
F. Analysis of the total loss and its influence to the GBS experiments 9
G. Validation of GBS 12
H. Extended discussion: Implement time-domain GBS with displacement 13
I. Extended discussion: Realize photon-number resolving detection in time-domain GBS 14

III. Encoding a graph onto GBS machine 15

IV. Detailed results on the clique search 15

V. Brief introduction to the inverse virtual screening platform, and details about the molecules used in experiments 19
A. The PARP1-inhibitor complex (corresponding to Fig. 2a) 19
B. The TACE-inhibitor complex (corresponding to Fig. 2b) 23

VI. Details about the molecular docking experimental results 28

VII. Details about the GBS-based RNA folding prediction 29
A. Example 1 (corresponding to Fig. 2c) 29
B. Example 2 (corresponding to Fig. 2d) 30

VIII. Further scaling and quantum computation advantages of our setup 31

IX. Further discussion about classical combinatorial optimization methods 38
A. Comparison with Genetic Algorithms 38
B. Comparisons with other quantum or quantum-inspired approaches 39

References 40

I. BRIEF REVIEW OF GAUSSIAN BOSON SAMPLING

Gaussian boson sampling (GBS) [1, 2] is a non-universal model of quantum computing and a variant of boson sampling (BS) [3, 4], for which the squeezed states instead of single-photon Fock states are input and evolved under a linear transformation .

The input states can be any Gaussian state such as vacuum state, squeezed vacuum state, coherent state, squeezed coherent state or thermal state. Supplementary Figure 1(a) shows an example of GBS with eight modes, and the input states are squeezed vacuum states. If the Gaussian state has zero displacement, and photon-number-resolving detectors (PNRDs) are used, the photon-number distribution can be described by the Hafnian of the submatrix of A [1, 2]:

$$\Pr(s) = \frac{\mathrm{Haf}(A_s)}{n_1!n_2!\cdots n_N!\sqrt{\det(\sigma + \mathbb{I}/2)}}, \tag{1}$$

where A (A_s is submatrix of A) contains squeezing parameters and linear transformation information and can be written as

$$A = \begin{pmatrix} 0 & \mathbb{I} \\ \mathbb{I} & 0 \end{pmatrix} [\mathbb{I} - (\sigma + \mathbb{I}/2)^{-1}], \tag{2}$$

where σ is the covariance matrix. Here, $s = (n_1, n_2, \cdots, n_M)$ is the photon-number distribution pattern, M is the number of modes, n_i denotes the number of photons in mode i, and the total number of photons is denoted by $N = \sum_{j=1}^{M} n_j$. The $2N \times 2N$ submatrix A_s is constructed by repeating the ith and $(i+M)^{\mathrm{th}}$ row and column of A n_i times. As shown in Supplementary Figure 1(b) and (c), when the second, third and seventh outputs are detected photons (shown in Supplementary Figure 1(a) for example), the submatrix A_s then will be constructed by selecting the elements located at both of 2nd, 3th, 7th, and 10th, 11th, 15th rows and columns in A.

The hafnian of a matrix is defined by:

$$\mathrm{Haf}(A) = \frac{1}{n!2^n} \sum_{\sigma \in S_{2N}} \prod_{i=1}^{N} A_{\sigma(2i-1)\sigma(2i)}, \tag{3}$$

where S_{2N} is the set of all permutations of $2N$ elements. Especially, we note that in graph theory, the hafnian counts the number of perfect matchings in an arbitrary graph with adjacency matrix A [5].

In particular, the block matrix A (as illustrated in Supplementary Figure 1(b)) can be reduced to $A = B \oplus B^*$ (i.e., $C = 0$) if the Gaussian state is pure. Then, the probability of photon-number distribution can be written as:

$$\Pr(s) = \frac{|\mathrm{Haf}(B_s)|^2}{n_1!n_2!\cdots n_N!\sqrt{\det(\sigma + \mathbb{I}/2)}}. \tag{4}$$

Supplementary Equation (1) and (4) are also applicable when threshold (single-photon) detectors are used and the experiments are run in the collision-free space (i.e., n_i=0 or 1). The general expression for threshold detector GBS output probability is given by:

$$\Pr(s) = \frac{\mathrm{Tor}\left[\begin{pmatrix} 0 & \mathbb{I} \\ \mathbb{I} & 0 \end{pmatrix} A_s\right]}{\sqrt{\det(\sigma + \mathbb{I}/2)}}, \tag{5}$$

where $\mathrm{Tor}(\cdot)$ denotes torontonian, which is defined as:

$$\mathrm{Tor}(A) = \sum_{Z \in 2^{\mathcal{M}}} (-1)^{|Z|} \frac{1}{\sqrt{\det(\mathbb{I} - A_Z)}}, \tag{6}$$

where $\mathcal{M} = \{1, 2, \ldots m\}$ and $2^{\mathcal{M}}$ is its power set [6].

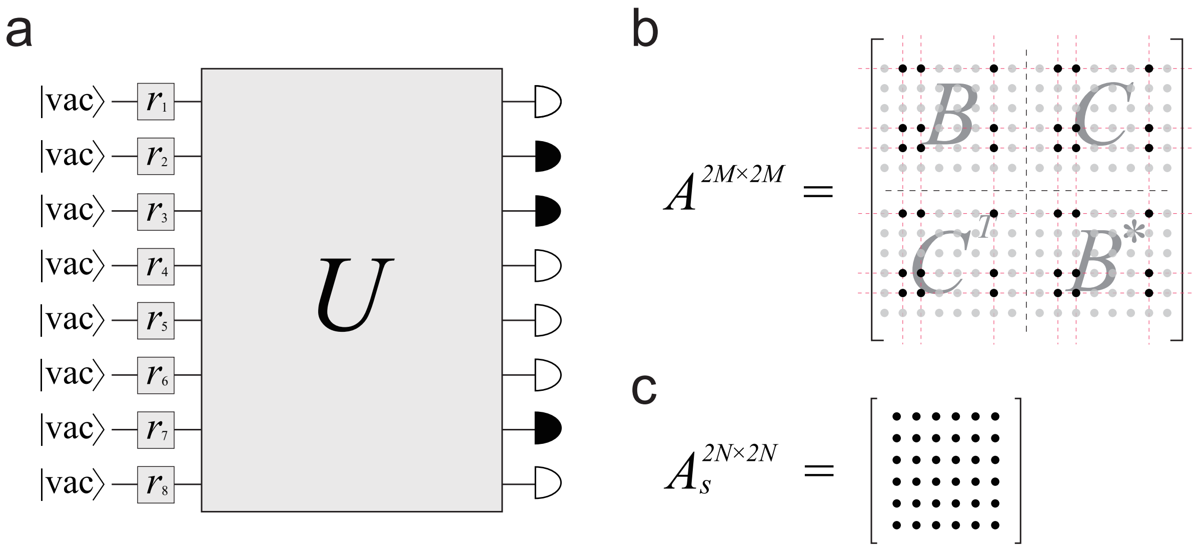

Supplementary Figure 1: **GBS concept and the formulation of the submatrix A_s. a,** An overview of the GBS setup (we use a eight-mode scheme as an example here). Eight squeezed vacuum states with different squeezing parameters r_i are injected into a unitary transformation U, and detected by single-photon or photon-number-resolving detectors. **b,** A is a matrix with $2M \times 2M$ dimension (here we choose $M = 8$ for corresponding to **a**). The red dashed lines shows the rows and columns corresponding to the modes which detect the photons shown in **a**. **c,** The submatrix A_s extracted from A.

II. EXPERIMENTAL DETAILS

A. Squeezed light sources

The 80 MHz 140 fs pulsed pump light from a Ti:sapphire mode-locked laser (Chameleon) operating at 773 nm is reduced in rate by an acoustic-optic modulator (AOM) into a series of pulse sequences which contains n pulses (i.e., n time-bin modes) with 40 MHz repetition rate, as shown in Supplementary Figure 2. After that, an electro-optic modulator (EOM) and a polarizing beam splitter (PBS) are used to modulate the pump power. The EOM is controlled by a computer, and the squeezing parameters (r_i) of each squeezed state can be adjusted by changing the pump power [7].

Then, a spectral shaper, which consists of a spatial light modulator (SLM) inside a 4f system, is used to tailor the bandwidth of the pump light. After the spectral shaper, the pump pulses are engineered with an approximate spectral width of 1.9 nm and are coupled into the 10 mm long periodically-poled KTP (ppKTP) waveguide to generate telecom-wavelength near-degenerate two-mode squeezed vacuum (TMSV) [7–10]. Supplementary Figure 3(a) shows the joint spectrum intensity (JSI) after filtering, which is measured by coincidences between signal and idler photons. In this situation, JSI is generated in a near-degenerate manner with a purity of approximately 91%. In the experiment, a bandpass spectral filter is used to eliminate the sinc-side lobes around the central peak in the JSI.

The TMSV state can be expressed as [11]:

$$|\mathrm{TMSV}\rangle = \sum_{n=1}^{\infty} \frac{1}{\cosh r} \tanh^n r |nn\rangle. \tag{7}$$

We can convert the TMSV into two single-mode squeezed vacuum (SMSV) states by rotating the signal and idler modes to same polarization and interfering them at a 50 : 50 beam splitter [11]. Then, the SMSV in each output of the beam splitter can be written as [12]:

$$|\mathrm{SMSV}\rangle = \sum_{n=1}^{\infty} \frac{1}{\sqrt{\cosh r}} \frac{\sqrt{(2n)!}}{2^n n!} \tanh^n r |2n\rangle, \tag{8}$$

and the SMSV has the same degree of squeezing as the TMSV. The interference visibility of the two modes of TMSV

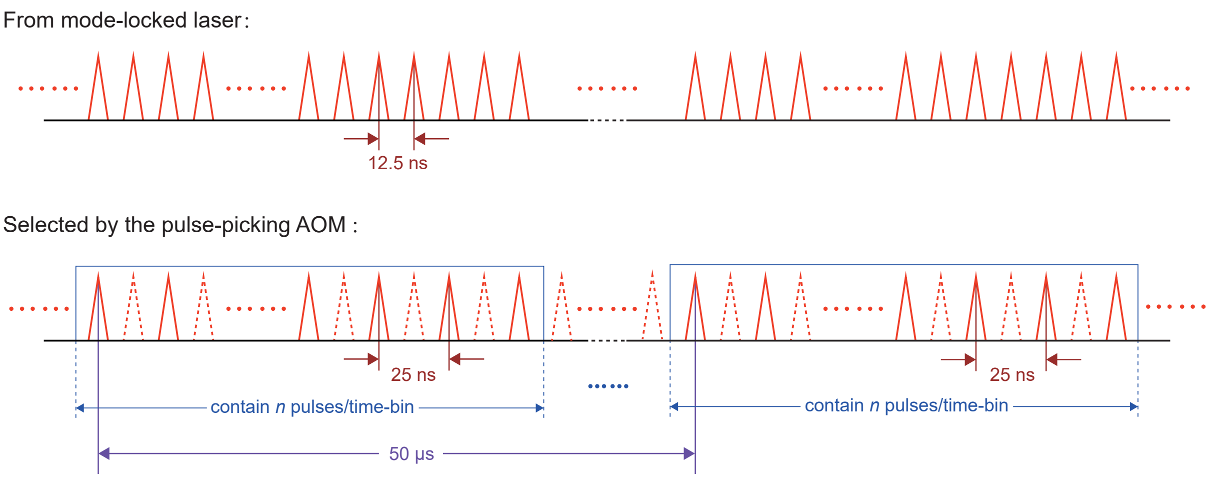

Supplementary Figure 2: **The pump pulses after picking by AOM**. A Ti:sapphire mode-locked laser deliveres pump pulses at a rate of 80 MHz. The pulse-picker AOM produces a series of n-pulse sequences at a rate of 20 KHz (i.e., the time interval between sequences is 50 μs). The pulses in the n-pulse sequence have time interval 25 ns (i.e., 40 MHZ).

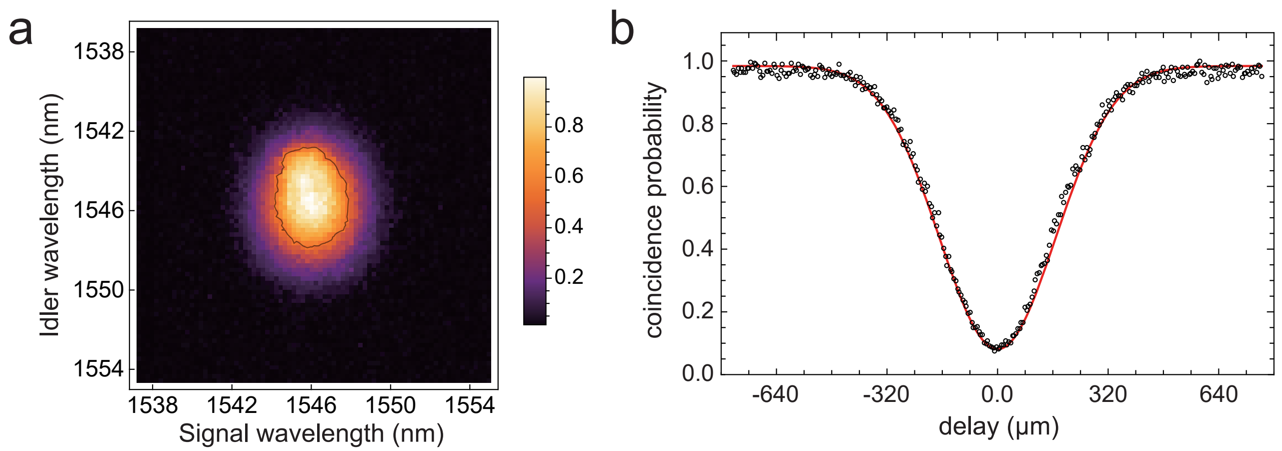

Supplementary Figure 3: **Purity of squeezed states from the source**. **a,** Joint spectral intensity of the TMSV state generated from ppKTP waveguide. The intensity is normalized here, and the black line indicates the region of 50%. **b,** Hong-Ou-Mandel interference results. The black circles are the experimental data (normalized) and the red line is the fitted result.

will determine the quality of the state. Therefore, we also test its Hong-Ou-Mandel interference, and the experimental results show the visibility is $91.53\% \pm 0.66\%$, shown in Supplementary Figure 3(b).

Here, we want to note that a problem of phase stability arises from the laser pulses of Ti:Sapphire mode locked laser. The phase of each pump pulse from Ti:Sapphire mode-locked laser changes according to the carrier-envelope phase, but in GBS experiments here, we need to ensure that all the SMSS have the same phase. In our experiments, since a 40MHz repetition rate laser pulse train is used in our experiments, we can assume that the phase of the pump pulses varies much more slowly than the repetition rate, and hence the phases of each time bin in the pulse train can be considered identical.

Now we demonstrate the tunability of the squeezing light source and illustrate the relationships between the squeezing level (r), the input pump power (P) and the normalized control signal (v) from arbitrary waveform generator (AWG) for controlling the electro-optic modulator (EOM). To measure these relationships, we conducted a step-by-step analysis by detecting the output average photon numbers. Specifically, as the squeezing level increased, we gradually increased the cycling loops to introduce additional loss while maintaining a low average photon number.

This approach ensures that the average number of photons can still be accurately measured, even when using a single photon detector.

Supplementary Figure 4 illustrates the detected squeezing level (r) under various input pump powers (P). In Supplementary Figure 4(a), the input power P refers to the power of the pump laser detected just before the coupling objective lens. The red line represents the fitting result with model $r = \alpha\sqrt{P}$, where this fitting model follows the one discussed in Ref. [7]. The fitting result shows $\alpha = 1.811$. In Supplementary Figure 4(b), we further demonstrate the relationship between squeezing level r and the normalized control signals v. As shown in Fig. 1(a) in the main text, the normalized control voltage v sent to EOM0 adjusts the power of the pump laser P according to the relation $P = A \times \sin^2(\pi/2 \times v)$, where A is the total average power of the pump laser, which in our experiment is 2.1 mW. The normalized control voltage v ranges from 0 to 1, where "0" corresponds to no pump laser being sent into the ppKTP waveguide, and "1" corresponds to the voltage amplifier providing half-wave voltage to EOM0, allowing all pump light to be fed into the ppKTP crystal. Since the EOM0 (and its voltage amplifier) used here can accept continuous analogue signal input, we can continuously and arbitrarily adjust r by changing the power of the pump light.

The blue line in Supplementary Figure 4(b) is plotted based on the function $r = \alpha\sqrt{2.1\sin^2(\pi/2 \times v)}$. This representation more intuitively demonstrates the relationship between the normalized control signal v and the squeezing level r. With this formula, we can conveniently obtain the required squeezing level by adjusting the voltage v of the control signal generated by the AWG.

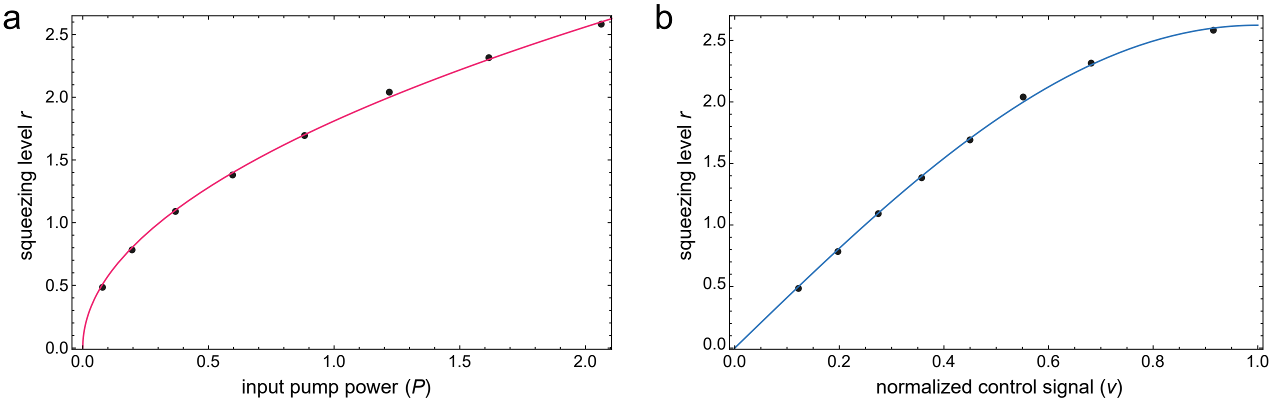

Supplementary Figure 4: **The relation between squeezing level r and input pump power P and normalized control signal v.** The red and blue lines represent the corresponding fitting results, serving as a guide for designing the control signal amplitude during experiments aimed at adjusting the squeezing level.

B. EOMs and the linear transformation

For a given unitary operation U, we can decompose it into a combination of SU(2) operations using Clements' architecture [13], as shown in Supplementary Figure 5. An $N \times N$ unitary can be reconstructed by C_N^2 SU(2) operations ($T_{j,i}(\theta,\varphi)$). As for the transformation between modes i and j ($j = i - 1$), $T_{j,i}(\theta,\varphi)$ can be expressed as:

$$T_{j,i}(\theta,\varphi) = \begin{pmatrix} 1 & 0 & \cdots & \cdots & \cdots\ \cdots & 0 \\ 0 & 1 & \cdots & \cdots & \cdots\ \cdots & 0 \\ \vdots & \ddots & & & & \vdots \\ \vdots & & e^{i\varphi}\cos\theta & -\sin\theta & & \vdots \\ \vdots & & e^{i\varphi}\sin\theta & \cos\theta & & \vdots \\ \vdots & & & & \ddots & \vdots \\ \vdots & & & & 1 & 0 \\ 0 & \cdots & \cdots & \cdots & \cdots\ 0 & 1 \end{pmatrix}, \tag{9}$$

which corresponds to a lossless beamsplitter between modes j and i with reflectivity $\cos\theta$ ($\theta \in [0, \pi/2]$) and a phase shift φ ($\varphi \in [0, 2\pi]$) at input j.

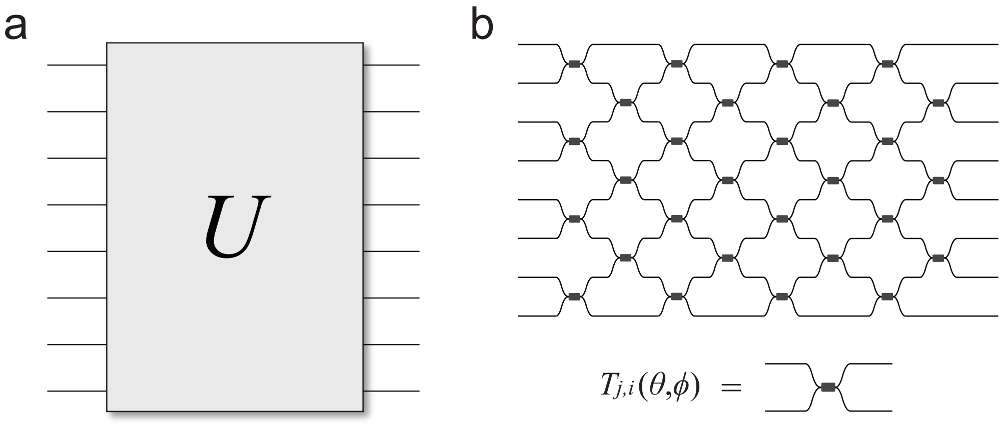

Supplementary Figure 5: **Decomposition of universal $n \times n$ multiport interferometer (shown here for $n = 8$ as an example)**. The interferometer shown in **a** can be decomposed into a series SU(2) operations $T_{j,i}(\theta, \varphi)$ as the architecture shown in **b**.

In experiments, we can use two low-loss, high-speed, customized electro-optical modulators (EOMs, Conoptics Model 360-RA-RP) to realize $T_{j,i}(\theta, \phi)$. The operation when the optical axis of the EOM is aligned to the polarization of the incident light can be written as

$$R_{\mathrm{EOM}:0^\circ}(\phi) = \begin{pmatrix} e^{i\phi/2} & 0 \\ 0 & e^{-i\phi/2} \end{pmatrix}. \tag{10}$$

The operation with the optical axis of the EOM at 45° to the incident light can be written as

$$R_{\mathrm{EOM}:45^\circ}(\phi) = \begin{pmatrix} 1 & 1 \\ -1 & 1 \end{pmatrix} \cdot \begin{pmatrix} e^{i\phi/2} & 0 \\ 0 & e^{-i\phi/2} \end{pmatrix} \cdot \begin{pmatrix} 1 & -1 \\ 1 & 1 \end{pmatrix} = \begin{pmatrix} \cos\phi/2 & i\sin\phi/2 \\ i\sin\phi/2 & \cos\phi/2 \end{pmatrix}. \tag{11}$$

This is the same as setting EOM at 0° (optical axis, only for shifting the phase), and placing two half wave plates (at 22.5°) before and after the EOM (this is exactly the same as the structure shown in Ref. [13]).

Then, we can use EOM1 and EOM2 shown in Fig. 1 in the main text to achieve $T_{j,i}(\theta, \phi)$:

$$\begin{aligned} T_{j,i}(\theta, \phi) &= e^{i\phi_1/2}\left[\begin{pmatrix} 1 & 0 \\ 0 & -i \end{pmatrix} . R_{\mathrm{EOM}:45^\circ}(\phi_2) \begin{pmatrix} 1 & 0 \\ 0 & i \end{pmatrix} . R_{\mathrm{EOM}:0^\circ}(\phi_1)\right] \\ &= e^{i\phi_1/2}\begin{pmatrix} \cos\phi_2/2 & -\sin\phi_2/2 \\ \sin\phi_2/2 & \cos\phi_2/2 \end{pmatrix} \cdot \begin{pmatrix} e^{i\phi_1/2} & 0 \\ 0 & e^{-i\phi_1/2} \end{pmatrix}. \\ &= \begin{pmatrix} e^{i\phi_1}\cos\phi_2/2 & -\sin\phi_2/2 \\ e^{i\phi_1}\sin\phi_2/2 & \cos\phi_2/2 \end{pmatrix}, \end{aligned} \tag{12}$$

where $\phi_1 = \varphi, \phi_2 = 2\theta$, and the global phase shift $e^{i\phi_1/2}$ can be compensated in the next loop. Namely, additional layers (corresponding to the last loop in QPU) are needed for phase compensation.

An important consideration is the phase stability in the time-bin interferometer (as shown in Fig. 1). A computer-controlled nano-displacement stage with piezoelectric scanning accuracy of < 1 nm (SmarAct SLC-17) is used to carefully adjust the time delay between two polarizations (shown in Fig. 1 in the main text with a roof prism mirror mounted on top). In our experiments, the GBS machine has a 20 KHz sampling rate, namely, 10^5 samples can be collected within 1 second. The phase in the interferometer varies much more slowly than the experiment time since we only collect 10^6 samples in one experiment. Thus, we do not use a phase-lock system in our setup, and we can manually check the phase after each experiment.

C. Control system

The control system is a very important component of the experiment (not shown in Fig. 1 in the main text), since it enables the programmability of the GBS machine. There are six computer-controlled elements in total: AOM (acts as a pulse-picker, discussed in Section II.A), EOM0 (controls the squeezing (r_i), Section II.A), EOMa/b (fast optical switches, Section II.B), and EOM1/2 (components of the MZ-interferometer, Section II.B).

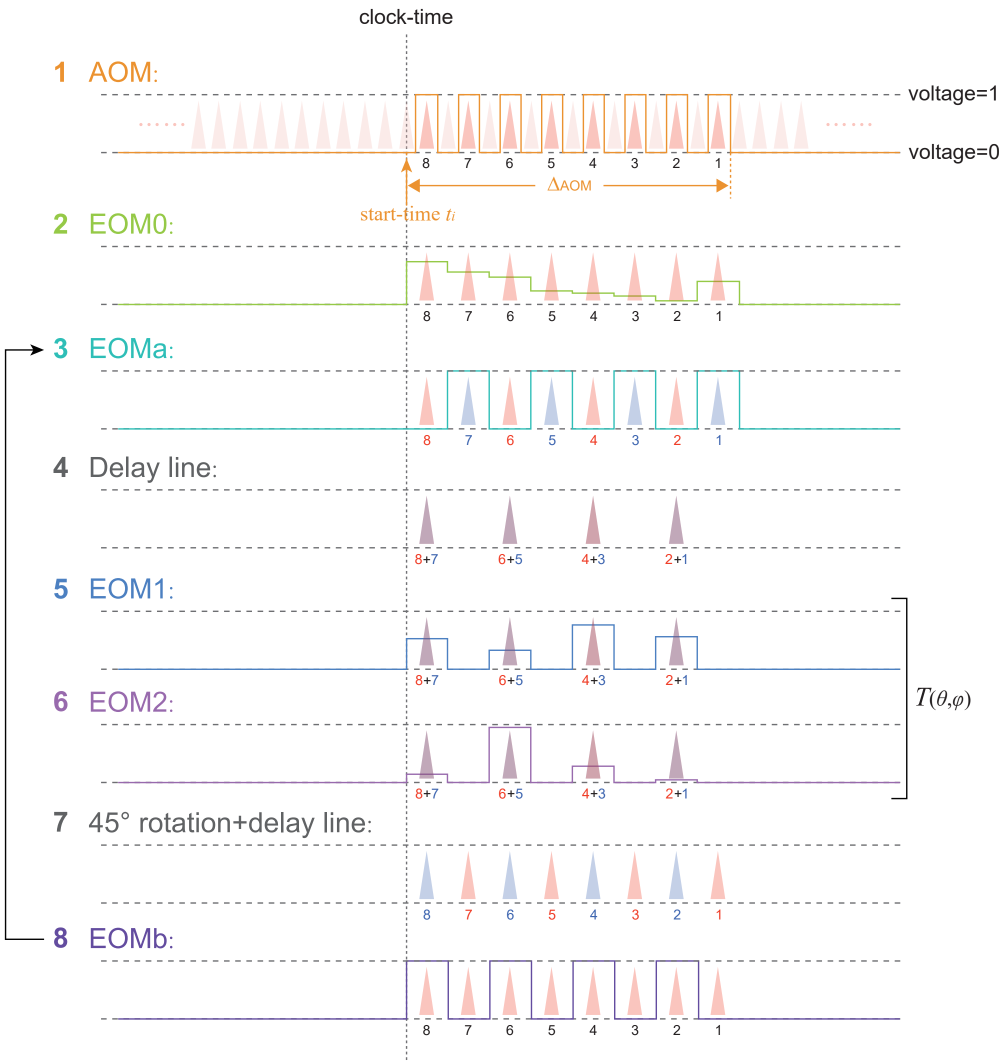

Supplementary Figure 6: **Control sequences for one experiment.** The control sequences for the key elements using an eight-mode GBS experiment as an example. Here, EOM1 (optical axis is set at 0°) varies φ, and EOM2 (optical axis is set at 45°) varies θ.

In our experiments, the above elements are software-controlled by arbitrary waveform generators (AWGs). The program can support arbitrary waveforms and control of three AWGs simultaneously.

The start-time (t_i) of AOM is set as the clock-time. Here, we use the time of pulses from the laser to perform a pre-calibration to ensure that the start-time of AOM lies at an appropriate time between the two laser pulses (as shown in Supplementary Figure 6). The start time τ_{AOM} of the AOM also determines the scale of our GBS experiment. If n modes are required for an experiment, AOM will then start at the time between t_i and $t_i + \Delta_{\text{AOM}}$ (where Δ_{AOM}=$n \times 25$ ns, since the interval of each pulse is 25ns as discussed in Section II. A), and select n pulses to send to subsequent experiments.

EOM0 modulates the power of each pulse by sending a continuous analog signal sequence (shown as Supplementary Figure 6, the second line), starting at time $t_i + \tau_{\text{EOM0}}$, where τ_{EOM0} can be carefully calculated according to the

optical distance between AOM and EOM0.

EOMa is a fast switch to change the polarization of each pulse (shown as Supplementary Figure 6, the third line). It turns on at time $t_i + \tau_{\text{EOMa}}$, and a digital signal sequence is added. The following 7.5 m free-space delay-line combines the adjacent pulses, and this delay-line can be adjusted precisely to the interval time of each pulse (25 ns) by a roof prism mirror mounted on the nano-displacement stage.

Then, two continuous analog signal sequences are sent to EOM1 and EOM2 at time $t_i + \tau_{\text{EOM1}}$ and $t_i + \tau_{\text{EOM2}}$ for adjusting θ and φ, respectively (as shown in Supplementary Figure 6).

Finally, another fast switch, EOMb changes the polarization of each pulse by sending a corresponding digital signal sequence at time $t_i + \tau_{\text{EOMb}}$ (as shown in Supplementary Figure 6), and separates them by going through the 7.5 m free-space delay-line again.

D. Quantum sequential memory with fibre delay line

In our experiments, we use both a free-space optical delay and a long fibre delay line to act as a quantum memory. Since the interval of each time-bin is 25 ns, we need to delay one time-bin by 25 ns (approximately 7.5 m), and combine the adjacent time-bin to perform the unitary operation determined by Supplementary Equation 12. Since this distance is relatively short and can be realized after several reflections in our optical platform, here we perform this part of the delay line through multiple reflections between a pair of rectangular mirrors coated with highly reflective film (as shown in Fig. 1 in the main text). A roof prism mirror is mounted on the nano-displacement stage which can be controlled by a computer to set the length of the delay line. It ensures that the polarization states formed by the combination of two adjacent time-bin modes have high coherence and stability. In order to reduce loss, all mirrors are coated with high reflectivity film optimized for 1546 nm. The throughout of this delay setup exceeds approximate 99% in experiments.

After EOMb, the time-bin sequence is sent into a long fibre to allow all the time-bin modes to be used in their corresponding operations. This module works as a quantum sequential access memory (QuSAM) since each time-bin can be read out in the original order, as required in our experiments. Here, we use a 180 m long fibre ($\leqslant$ 0.18dB/km at 1550nm) with transmittance $\approx 99.3\%$. An important issue here is the coupling efficiency, and a low coupling efficiency will exponentially reduce the efficiency of the whole device.

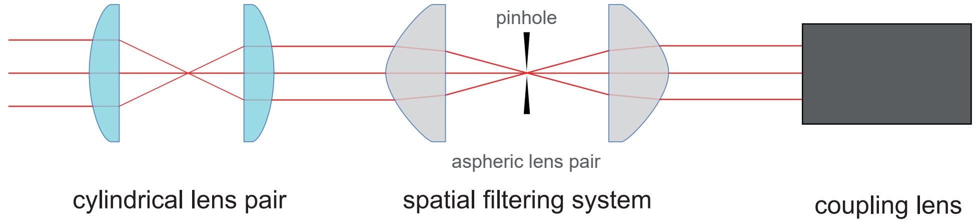

Supplementary Figure 7: **Details about the spatial shaper setup**.

After a series of optical elements, the spatial mode of light will become impure, and this will reduce the coupling efficiency from free space to single-mode fiber. Here, we build a spatial-mode re-shaper to improve the coupling efficiency (as shown in Supplementary Figure 7). A series of lens groups are applied to complete the shaping of the beam. First, we use a pair of cylindrical lens to reshape the spot, and then we send it to a pair of aspheric lenses. Assisted by a pinhole with suitable size, it forms a spatial filtering system which can effectively produce a uniform Gaussian beam. After carefully adjusting using the pair of cylindrical lenses, the transmission in the spatial filtering system can reach ≈ 0.990 in our experiment. Then, a lens is used to couple the light into single-mode fiber. The transmittance of the 180 m long fibre and the coupling efficiency can reach ≈ 0.946 overall, and the total efficiency including the spatial-mode re-shaper is approximate 0.940 in experiment. Furthermore, this 180 m-long delay line fibre will induce dispersion on each state. In our experiment, we note that since all the pulses go through same optical path, dispersion will negligible effect on the non-classical interference.

E. Detection system

In this experiment, since only threshold detection is required and the average number of photons is much less than the sampling size due to loss, superconducting nanowire single-photon detectors (SNSPDs) are used. The detection

efficiency is approximate 0.87. Due to the dead-time of SNSPDs (approximate 50 ns), and the interval of each time-bin 25 ns, we separate the time-bin sequence into two parts and set the interval of time-bins $\geqslant$ 50 ns.

Here, we build a simple demultiplexer with another EOM (Conoptics Model 360-RA-RP, the transmission is approximate 0.97), and change the polarization of the photons in time bins at even positions into vertical polarization and maintain horizontal polarization for the photons in odd time bins, as shown in Supplementary Figure 8.

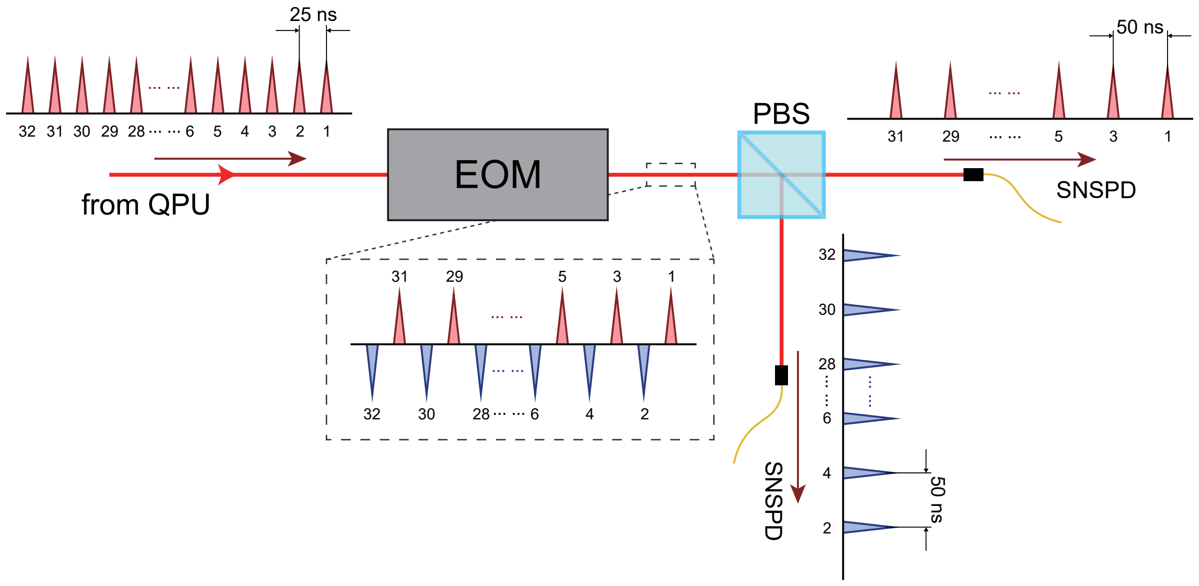

Supplementary Figure 8: **Details on the threshold detection.** After the signal light comes out from QPU, another EOM (same as EOMa or b) is applied for adjusting the polarization of photons in each time-bin. Here, the red signal denotes the pulses with horizontal polarization, and the blue signal denotes the pulses with vertical polarization. This is omitted from Fig. 1 in the main text.

By using two SNSPDs to detect the time-bin sequence separately, we can detect the photons and the photon distributions (as shown in Fig. 1(b and c)) successfully.

F. Analysis of the total loss and its influence to the GBS experiments

First, we list the losses introduced by each component:
The photon transmission efficiency of a single-loop in QPU is approximately 0.82, and this is caused by the following reasons:

- Efficiencies of the fibers (including both the collection efficiencies from free space to fibers and the transmission efficiencies in the fibers, as discussed in Section II.D): $\approx$0.940
- Transmission efficiencies of EOMa: $\approx$0.977, EOMb: $\approx$0.975, EOM1: $\approx$0.954 (double pass), EOM2: $\approx$0.976
- 7.5 m delay line: $\approx$0.995
- All other optical elements: $\approx$0.985

Therefore, the single-loop transmission efficiency can be calculated as $0.82 \approx 0.939 \times 0.977 \times 0.975 \times 0.954 \times 0.976 \times 0.995 \times 0.985$.

Outside the loop, the coupling efficiency from ppKTP waveguide to single-mode fibre is around 0.78, filter after ppKTP induce 0.06 loss (i.e., transmission efficiency 0.94), the coupling efficiency from demultiplexer to single-mode fibre is around 0.93, the transmission of EOM for changing the polarization of each pulse (in the demultiplexer) is approximate 0.97 (discussed in the above section), and the detecting efficiency of SNSPDs are approximate 0.87.

Thus, if we build a 32-mode GBS setup, the total transmission efficiency is $0.82^{33} \times 0.780 \times 0.944 \times 0.930 \times 0.973 \times 0.87 \approx 0.0008$. Here, 33 on the index at the first item indicates that the 32-dimension U operation requires 32 cycles (according to Clements architecture), and one additional cycle for phase adjustment (compensation) at the end. If

we inject 32 squeezed states with squeezing parameter $r = 2.6$, the average photon number is approximately 1.147 (theoretically calculated). This value matches well with the experimental data (1.067±0.096) we detected, as shown in Supplementary Figure 9.

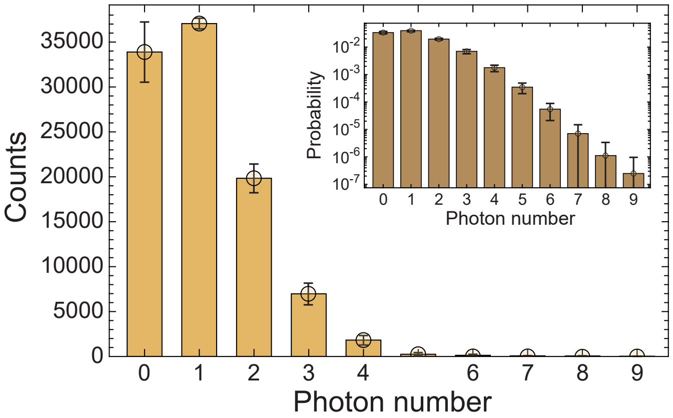

Supplementary Figure 9: **Photon number distribution of 32-mode GBS experiments**. All 32 squeezers are turned on (r_i are all set as 2.6) and the interferometer is selected randomly. The photon counts are shown as the orange bars, and the corresponding probabilities are plotted at inset with brown bars. The height of the bars (overlaid with the circles) are presented as mean values, and the error-bars are calculated by standard deviation with 20 experiments (10^5 samples are collected in each experiment).

Almost all the above losses can be regarded as uniform loss since the pulse train travels through the same path. The only non-uniform loss we need to consider is that induced by the 7.5m-delay-line. Non-uniform loss is unwanted because it results in a unitary different from what we prepare [14]. In our experiment, we reduce this non-uniform loss by using a free-space delay-line (in contrast to the fibre-line-delay, which usually brings a relatively large loss and dispersion), and the aforementioned high reflective film coating to make the transmission rate higher than 99.5%. This minimizes the impact of nonuniform loss on our experiment.

The influence of loss:
In GBS experiments, loss will give a different photon distribution compared with the ideal results. This can be estimated by numerical simulation (using Strawberry Fields [15]), and the results shown in Supplementary Figure 10 and Supplementary Figure 11, which correspond to the situations shown in Fig. 1(b) and (c) in the main text. In this section, we will analyze how the loss and squeezing influence the photon-number-distribution probabilities.

Here, we use the total variation distance (TVD) as a fidelity indicator, and it is defined as:

$$\mathrm{TVD} = \frac{1}{2}\sum_i |p_i - q_i|, \tag{13}$$

where p and q are probability distributions obtained from sampling results.

We first analyze the case of two-photon events sampled from a 32-mode GBS experiment (i.e., the case shown in Fig. 1(b) in the main text). Supplementary Figure 10(a) shows the simulation results of the ideal probability distribution, namely, the situation when loss is zero and $r_{1-3,32} = 2.23$. Supplementary Figure 10(b) shows the simulation results when loss and squeezing is set to the actual experimental value, i.e., $\xi = 0.9992$ and $r_{1-3,32} = 2.23$, respectively. By comparing it with the ideal results (i.e., lossless case), we find TVD = 0.2452. Ref. [12] shows that if we turn down the squeezing (r_i), the distribution pattern will be closer to the ideal situation at the cost of data acquisition time. In this case, TVD = 0.0140, as shown in Supplementary Figure 10(c).

Secondly, we analyze the case of four-photon detection events sampled from a 16-mode GBS experiment (i.e., the case shown in Fig. 1(c) in the main text). Supplementary Figure 11(a) is the simulation results of ideal probability distribution, and Supplementary Figure 11(b) is the simulation results when loss $\xi = 0.9816$ and squeezing parameter $r_{\max} = 1.8$, which is the actual experimental value. Similarly, comparing with the ideal results, we find TVD = 0.3003. Although the TVD is higher, profile still matches with the ideal result. We also calculate when squeezing is reduced to $r_{\max} = 0.1$, and TVD = 0.0945 this time (shown in Supplementary Figure 11(c)).

This result makes us consider that we need to choose an appropriate balance between the accuracy and efficiency of sampling. In Supplementary Figure 12, we plot the relation between TVD and squeezing. We find that when the

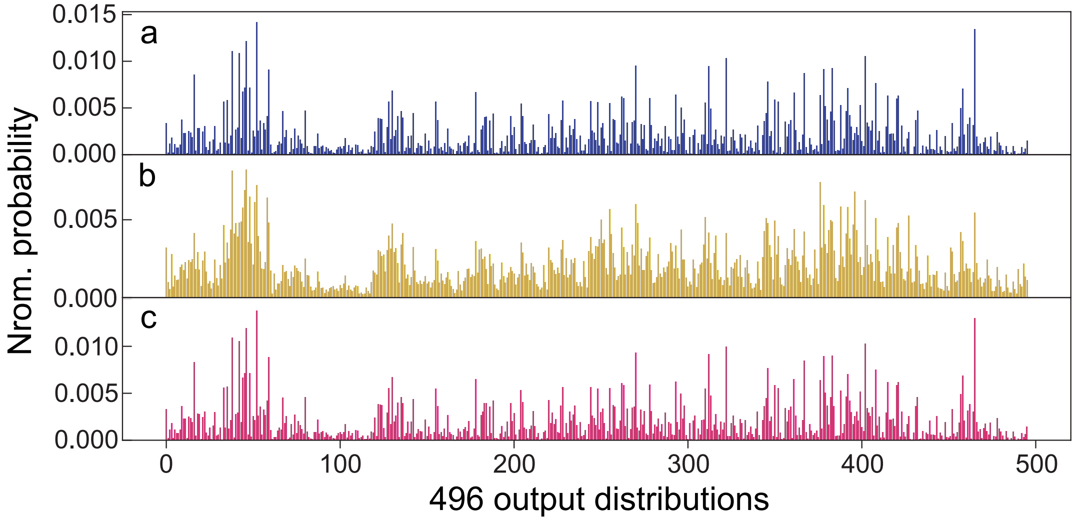

Supplementary Figure 10: **Numerical simulations of the probability distributions of two-photon events in 32-mode GBS with different squeezing**. **a,** The ideal result. **b,** The imperfection case when loss $\xi = 0.99929$ under squeezing $r = 2.23$. **c,** Results obtained by reducing the squeezing to $r = 0.1$ under same loss as **b**.

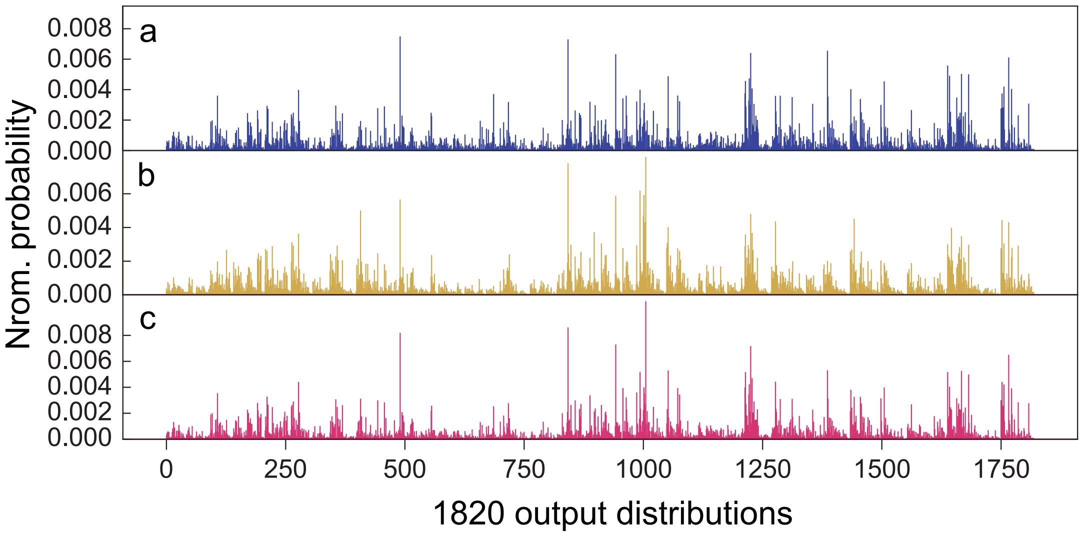

Supplementary Figure 11: **Numerical simulations of the probability distributions for the four-photon events in 16-mode GBS with different squeezing**. **a,** The ideal result. **b,** The case when loss $\xi = 0.9816$ under squeezing $r_{\max} = 1.8$. **c,** Results obtained by reducing the squeezing to $r_{\max} = 0.1$ under same loss as **b**.

degree of squeezing increases in a small range, TVD increases rapidly. However, when the squeezing reaches a large value, the TVD increases slowly and tends to saturation. This indicates under a fixed loss, if we can tolerate TVD under a large squeezing, we can appropriately increase the squeezing to improve the sampling rate without largely changing the TVD.

In our time-bin encoded GBS machine, the loss increases with the size of the interferometer. Supplementary Figure 13 shows the relation between TVD and loss, and it is clear that a larger loss will result in a larger TVD. It is interesting to find that a smaller TVD will be obtained under a larger sampling space when the loss is same. For example, when loss $\xi = 0.6$, TVD in 32-mode GBS experiment (496 sample space where all two-photon events are accounted) is approximately 0.24, while TVD ≈ 0.18 happened in 16-mode GBS experiment (1820 sample space where all four-photon events are accounted).

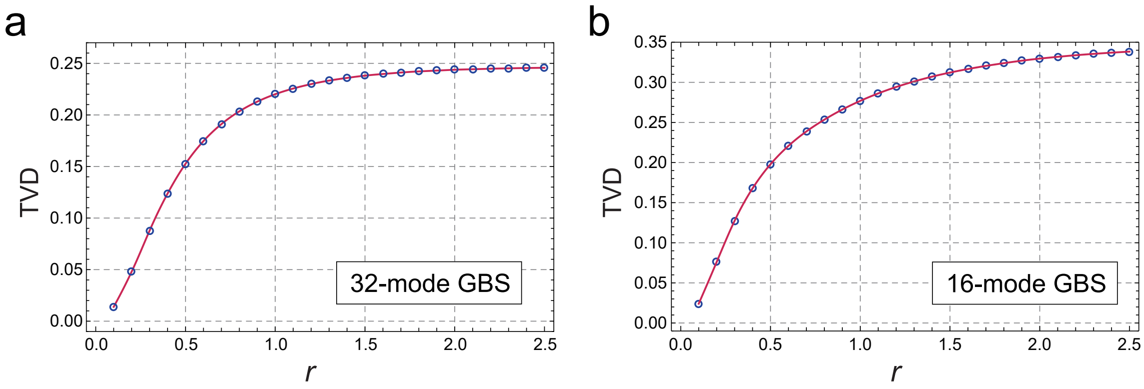

Supplementary Figure 12: **Numerical simulations of the relation between TVD and squeezing under a fixed loss**. **a,** The relation studied under the case shown in Fig. 1(b), i.e., 32-mode GBS experiment. **b,** The relation studied under 16-mode GBS experiment (Fig. 1(c)). In both cases, we find TVD will converge when the squeezing turns large.

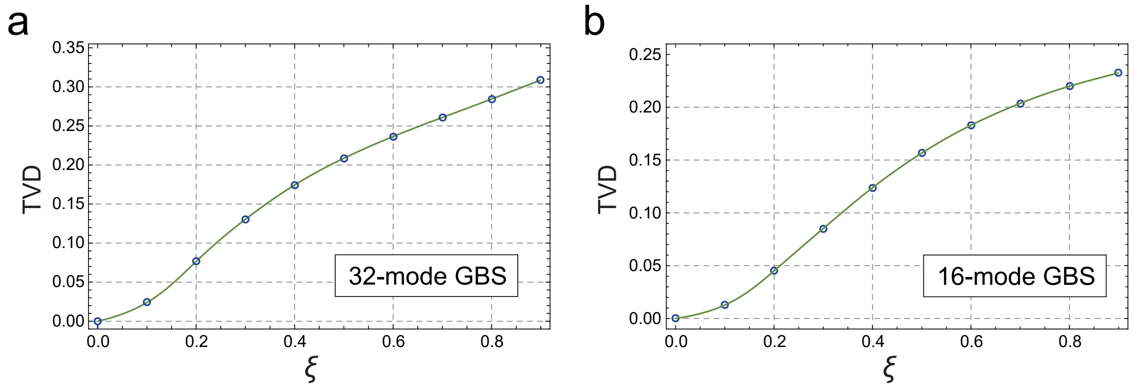

Supplementary Figure 13: **Numerical simulations of the relation between TVD and loss under fixed squeezing**. **a,** and **b,** show the relation studied under 32-mode and 16-mode GBS experiments, respectively. Not surprisingly, the loss increases the sampling error.

From these evidences, we can find a small squeezing will provide a higher fidelity result when loss existed, and same phenomenon is also found in the recent work [12]. This means we can decrease the bright of the squeezed states to obtain a better sampling results but sacrifice the sampling rate.

The issue of large loss is present in all known GBS experiments [16, 17]. Even if we only use bulk optical elements, there still exists approximately 30% loss in experiments [16]. One therefore needs to take these losses into consideration when mapping GBS to practical applications. Lossy GBS experiments sample from a mixed state instead of from a pure state, which can be mimicked as sampling from a pure state with random displacements. Thus, studying GBS with displacements [18] is an interesting direction in the future.

G. Validation of GBS

To validate our sample results are generated by a true quantum sampler, we utilize the modified likelihood ratio test introduced in [19]. This test incorporates a discrimination threshold that can compensate for experimental noise, and allowing us to rule out the hypotheses of thermal states and distinguishable photons.

We classically simulate the sampling results under the conditions of thermal states and distinguishable photon inputs. For the thermal state hypothesis, we assumed that the input states in each mode are single-mode thermal states with the same average number of photons. Under the distinguishable photon hypothesis, we considered a situation where the probability distribution is calculated based on a set of independent single-photon experiments,

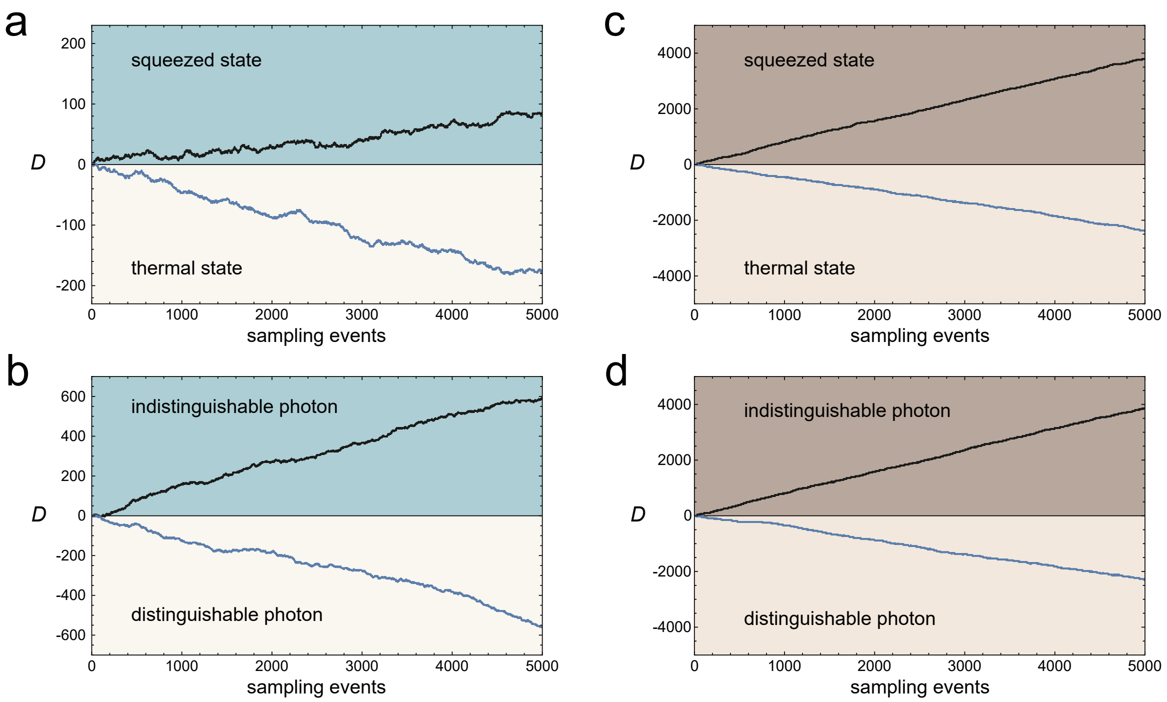

Supplementary Figure 14: **Modified likelihood ratio tests**. The variable D functions as a likelihood ratio test counter. Ideally, it will increase with each sampling event attributed to GBS and decrease with each event attributed to the thermal (or distinguishable) sampler. When $D > 0$, the sampling results are attributed to GBS (input with indistinguishable squeezed state). **a**, **b** These figures show the modified likelihood ratio tests with samples collected from the 32-mode, 2-click situation. **c**, **d** These figures illustrate the tests with samples collected from the 16-mode, 4-click situation. Black points represent the test applied to experimental data, while blue points signify the test applied to simulated data, generated by either the thermal or distinguishable sampler.

effectively eliminating any interference between each photon.

We set $L_i = P_i^{\text{squeezed}}/P_i^{\text{thermal}}$ (or $L_i = P_i^{\text{indist}}/P_i^{\text{dist}}$), where P_i^{squeezed} and P_i^{thermal} (P_i^{indist} and P_i^{dist}) are the probabilities associated with squeezed and thermal states (indistinguishable and distinguishable photons) for the measured outcome. We calculate L_i for each sample and update a discrimination parameter D with rule $D = D + 2$ if $L_i \geqslant 1/a_1$; $D = D + 1$ if $1/a_2 \leqslant L_i < 1/a_1$; $D = D$ if $a_2 \leqslant L_i < 1/a_2$; $D = D - 1$ if $a_1 \leqslant L_i < a_2$; $D = D - 2$ if $L_i < a_1$. In our analysis, we set $a_1 = 0.65$, and $a_2 = 0.87$.

Using the experimental data and the corresponding simulated thermal and distinguishable photon sampling results, we do this modified likelihood ratio test, and the results are shown in Supplementary Figure 14. We observed a persistent deviation between our experimental GBS results and the hypothetical samplers (i.e., thermal state and distinguishable photons). When D is greater than zero, this means that the sampled distribution resembles GBS more than the other assumptions of thermal state and distinguishable photons. As the gap between the discrimination counter D of experimental results and the hypothesis sampler increases, we can exclude both of these two hypotheses with high confidence.

H. Extended discussion: Implement time-domain GBS with displacement

Our time-domain GBS machine *Abacus* can completely be possible to add the displacement operation $D(\alpha)$, and its realization is shown in Supplementary Figure 15.

For realizing the displacement operation, we need to build a more complex photon source module. An Optical Parametric Oscillator (OPO) is placed after mode-locked laser (Chameleon), which is used to provide the seed pump laser, for generating 1550nm laser. Then, the 1550nm laser is split into two paths.

In the bottom path, frequency of the laser is doubled in a periodically poled lithium niobate (PPLN) crystal and subsequently pump a periodically poled potassium titanyl phosphate (ppKTP) waveguide that produces degenerate two-mode squeezed vacuum state. The EOM1 before the spectrum shaper is used for tuning the power of pump laser

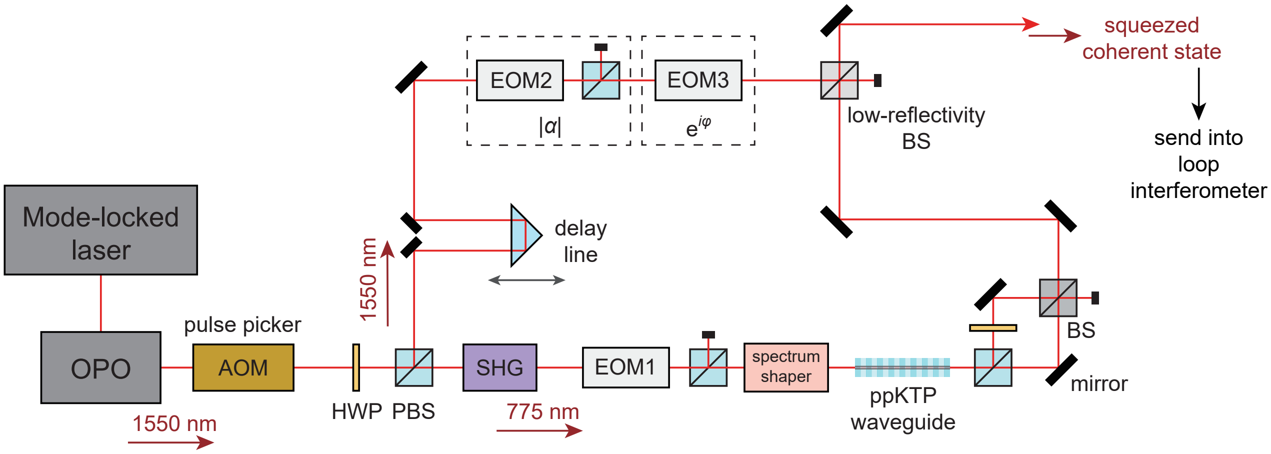

Supplementary Figure 15: **The photon source incorporates a displacement operation**. The displacement operation is achieved by superimposing coherent light with the squeezed vacuum state. EOM2 and EOM3 are employed to adjust the corresponding amplitude, $|\alpha|$, and phase, φ, parameters in the displacement operation.

and realizing the programmability of the squeezing level. This part is quite similar with the setup shown in Fig. 1(a).

The displacement operation is achieved using a local oscillator (LO) in the top path, following a similar method discussed in our recent work [18]. In the top path, we prepare a coherent state to serve as the LO for the displacement operation. The delay line ensures that the coherent state and squeezed state reach the beam splitter (BS) simultaneously, allowing for optimal interference at the output. EOM2 facilitates rapid polarization changes of the coherent states, enabling programmability of the displacement amplitude with the assistance of the polarizing beam splitter (PBS). It is worth noting that due to the presence of a low-reflectivity beam splitter in the subsequent optical path, the amplitudes of the adjusted coherent states should actually be multiplied by τ, where τ represents the reflectivity of the low-reflectivity beam splitter. EOM3 serves as a phase shifter to adjust the phase of the coherent state. Finally, a low-reflectivity beam splitter combines the signal state with a strong coherent state.

We also want to note that, in actual experiments, the phase-locking devices, including a piezo and a proportional¨Cintegral¨Cderivative controller need to be added to the delay line to ensure phase stability [18]. For simplicity, the phase-locking module is not shown here.

I. Extended discussion: Realize photon-number resolving detection in time-domain GBS

Here, we will discuss about how to implement photon-number resolving detection in time-domain GBS with transition edge sensor (TES). Besides the ability to distinguish photon numbers, TES usually need 5 μs to return to its original temperature after a photon absorption event. Therefore, the repetition rate of TES detectors is usually limited to around 100-300 kHz. This necessitates the installation of a demultiplexer in our time-domain GBS setup. In the recent paper [20], a 16-channel demultiplexer has been proposed. Here, we present a more concise demultiplexer.

In Supplementary Figure 16, we demonstrate the implementation of a 9-channel demultiplexer using one electro-optic modulator (EOM). The EOM allows us to manipulate the polarization of photons in each time-bin, determining whether they exit the system via the polarizing beam splitter (PBS) or undergo internal reflection within the loop. In Supplementary Figure 16(a), we observe that the final time-bin, labelled as I, passes through the EOM and directly exits via the PBS. In Supplementary Figure 16(b), the second-to-last time-bin, labelled as H, undergoes polarization modification controlled by the EOM and completes one loop in the circuit. By adjusting the positions of the two mirrors at the bottom, we ensure that the time-bin H precisely aligns with the next time-bin I at the EOM, allowing both to exit the loop simultaneously. Similarly, in Supplementary Figure 16(c), the third-to-last time-bin, labelled as G, undergoes two loops controlled by the EOM and synchronously meets the last two time-bins. Regarding the first time-bin, labelled as A, it requires a delay of 8 loops before exiting via the PBS (as shown in Supplementary Figure 16(d)).

In summary, we can manipulate the polarization of a series of time-bins using a single EOM, maintaining vertical polarization for internal reflection within the loop and transitioning to horizontal polarization for exit (as shown in Supplementary Figure 16(d)). This approach eliminates the need for multiple EOMs. However, due to the different

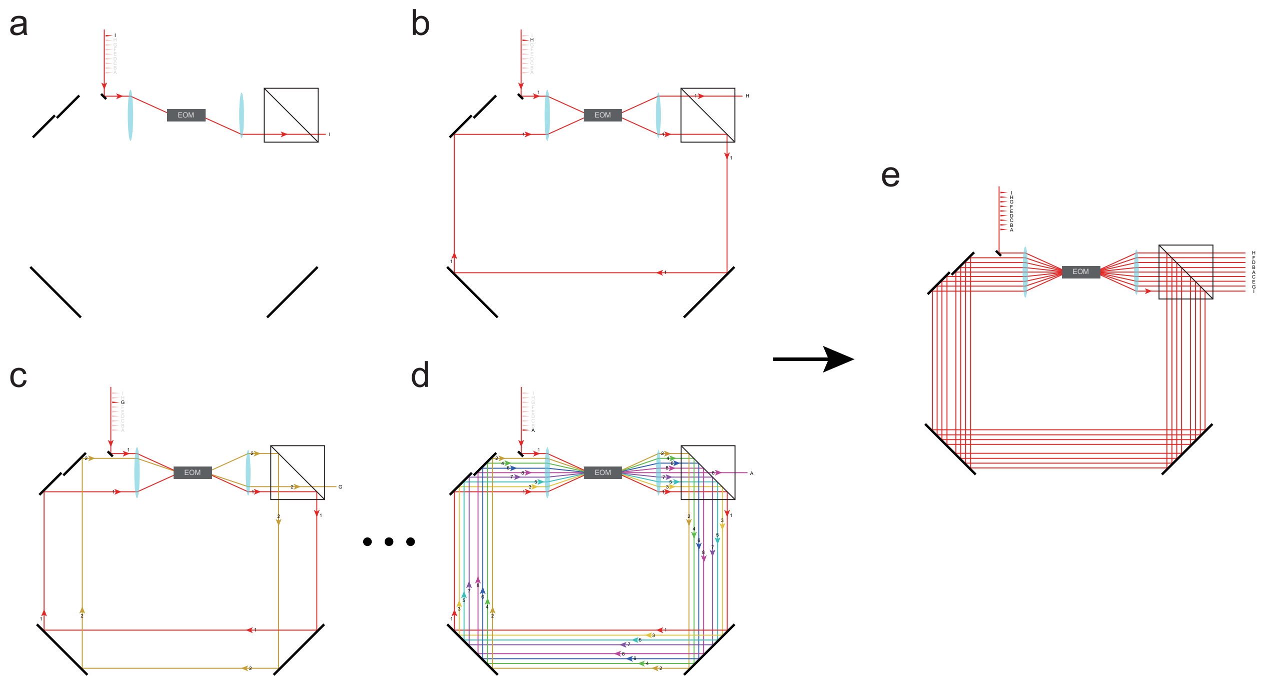

Supplementary Figure 16: **Demultiplexer with one EOM**. **a** to **d** represent the number of cycles required for the demultiplexer to process the time bins at their respective time positions before being emitted. The overall schematic diagram of this 9-channel demultiplexer is depicted as **e**.

number of loops experienced by each time-bin, non-uniform loss is introduced. To mitigate this, we utilize EOMs with high transmission rates (above 99%) and apply high-reflectivity coatings on all reflective surfaces. This helps minimize the discrepancies in non-uniform loss across the time-bins. It is worth mentioning that a similar demultiplexer proposal has been recently published in a paper by L. M. Hansen et. al. [21], although our demultiplexer design differs slightly from theirs.

III. ENCODING A GRAPH ONTO GBS MACHINE

For the node-weighted graph $\mathcal{G}$, we can define a matrix $B = \Omega(D - A)\Omega$, where A is the adjacency matrix of $\mathcal{G}$, Ω is a diagonal matrix with elements $\Omega_{ii} = c(1 + \alpha\omega_i)$ and weights ω_i, and D is the degree matrix of A defined as $D_{ii} = \sum_j A_{ij}$. In order to find suitable squeezing parameters of the input states, and guarantee the spectrum of B within $[0, 1)$, we need to carefully choose the parameters c and α in experiment. Based on the properties of A, it is clear that the matrix B is a symmetric matrix, and we can decompose it into $U \oplus_i^N \tanh(r_i) U^T$. Here, r_i are the squeezing parameters we need to adjust in the experiments, and U can be programmed with the time-bin encoded interferometer. Then, we can detect the cliques from the sampling results with the help of post-processing [25].

As shown in Supplementary Figure 17, a eight-node graph is encoded into the GBS circuit using the method discussed above, and the subgraph can be reconstructed based on where the photons are detected. Due to the errors in experiments, we also need to check whether it is a clique [5].

IV. DETAILED RESULTS ON THE CLIQUE SEARCH

The detailed information of the graph $\mathcal{G}_{32}$ we applied in the main text Fig. 1(d), and more experimental results are shown here. Supplementary Figure 18 depicts all the six-node cliques we find from 328 experimental data and local search with 30 iterations are applied in this case. We found nine six-node cliques in total, and the maximum weighted clique is found with the highest probability. We need to note here that the highest probability may occurs at other cliques but not the maximum weighted one at some times. We think that this is caused by experimental errors (especially the large losses), and the randomness of the local search process. But in any case, the successful

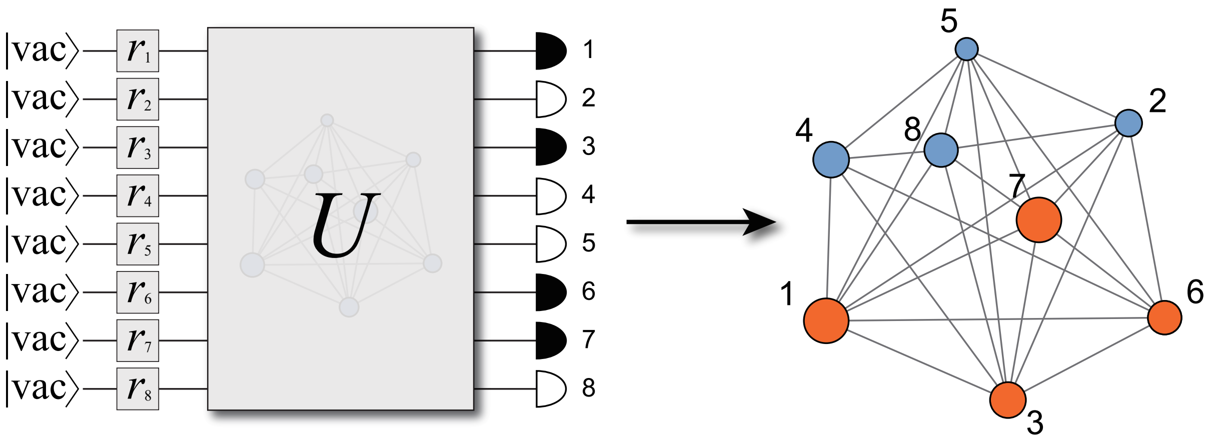

Supplementary Figure 17: **Encoding a graph onto GBS setup and finding the cliques**. The eight-node GBS circuit (assumed lossless) is programmed with a graph shown on the right side. There are four events (i.e., first, third, sixth, and seventh) are detected at outputs, and this indicates a four-node subgraph shown as orange circles.

rate in finding the maximum weighted clique always changes in a relatively fixed range and is always better than the classical random sampling results (shown in Supplementary Figure 20).

Supplementary Table 1 exhibits the weight of the vertexes in graph $\mathcal{G}_{32}$, and Supplementary Figure 19 shows the spectrum of single value decomposition after rescaling and adjacency matrix of $\mathcal{G}_{32}$.

**vertex**	**weight**
3, 5, 13, 18, 24, 25, 29	1.74
2, 7, 9, 16, 22, 32	0.36
1, 8, 23, 27, 31	1.00
17, 21, 28, 30	0.85
4, 6, 10, 11, 12, 14, 15, 19, 20, 26	1.37

Supplementary Table 1: **The weight of each vertex in the 32-node graph**.

In order to showing the advantage of GBS-based maximum weighted clique finding, we compare the GBS results with the classical sampling results. For fair comparison, we make the number of classical samplers same as the number obtained in experimental sampling, and the same number of local searching iterations is used to post process the classical sampling data. The probabilities of finding six-node cliques using classical sampling data are shown in Supplementary Figure 20. We can see that, in this case, the probability of finding the maximum weighted clique does not have advantages.

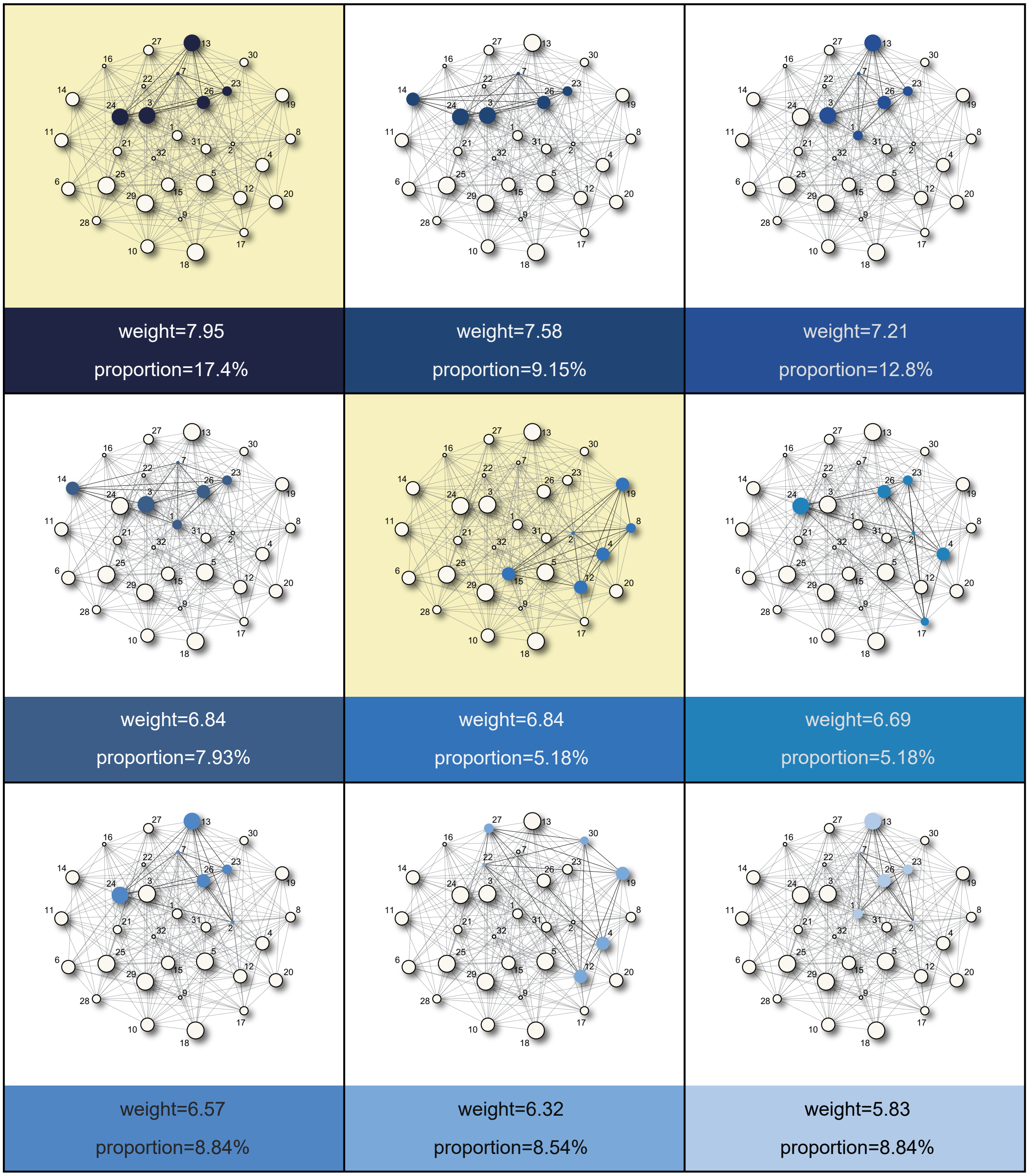

Supplementary Figure 18: **Detailed results data of the clique search**. The subgraphs with yellow background are the two shown in the main text.

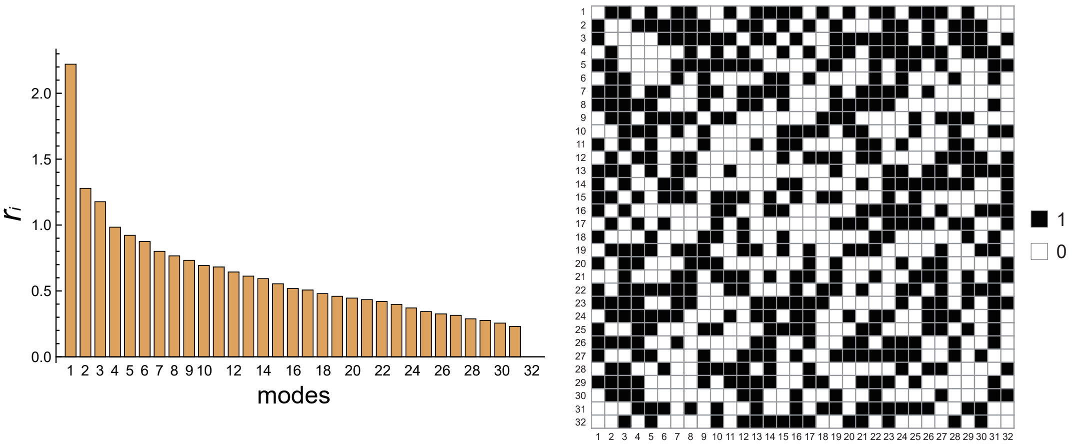

Supplementary Figure 19: **Details about the spectrum of single value decomposition (squeezing parameters r_i) and adjacency matrix of this 32-node graph**.

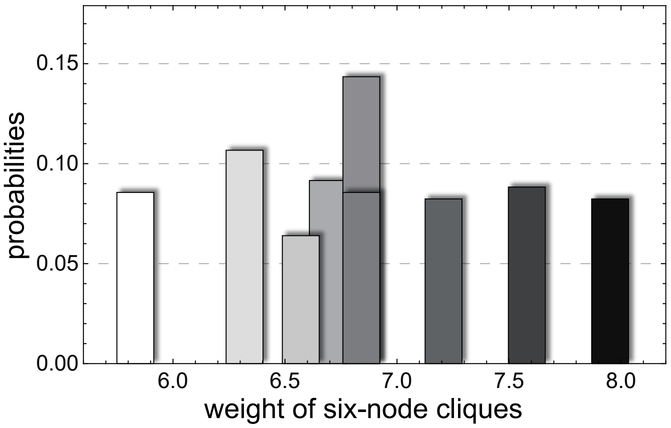

Supplementary Figure 20: **Success rate of finding cliques with classical random sample data**. The probabilities of six-node cliques obtained from classical random sampling. Greedy shrinking and 30 times local search expansion are also applied here as same in Fig. 1(d) in the main text. Comparing with GBS, it is clearly that the successful probability to find the maximum weight clique with classical sampling is only about half.

V. BRIEF INTRODUCTION TO THE INVERSE VIRTUAL SCREENING PLATFORM, AND DETAILS ABOUT THE MOLECULES USED IN EXPERIMENTS

The inverse virtual screening platform

Assuming n and m pharmacophore points are considered in the ligand and the potential protein, respectively, then the adjacency matrix of BIG ($\mathcal{G}_{\text{BIG}}$, Supplementary Figure 21) will be a $nm \times nm$ symmetric matrix $\mathcal{A}$, with diagonal elements that are all zero. Other elements are determined by the distances of the pharmacophore pairs in the ligand and the protein (the dashed lines in boxes 3 and 4, as shown in Supplementary Figure 21), respectively. As for the $\mathcal{A}_{ij}$, if the distance difference between corresponding pharmacophore pair in ligand (D_{L}) and protein (D_{P}) does not exceed $\tau + 2\varepsilon$ (where τ is the flexibility constant, and ε is the interaction distance), i.e., $D_{\text{P}} - D_{\text{L}} < \tau + 2\varepsilon$, we set $\mathcal{A}_{ij}$ and $\mathcal{A}_{ji}$ to 0. Otherwise, we set them as 1.

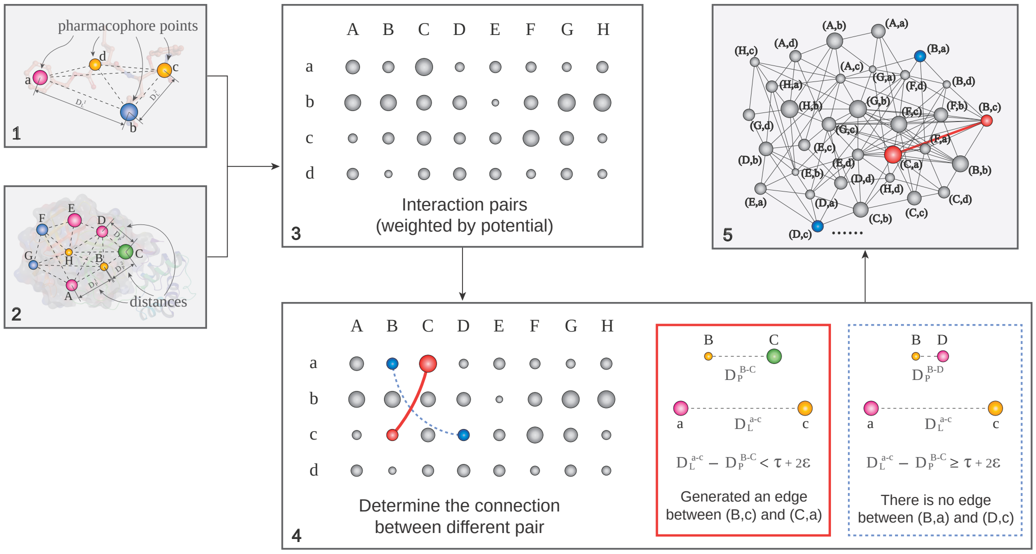

Supplementary Figure 21: **The criterion of edge generation in BIG**.

To highlight *Abacus*'s proficiency in molecular docking, we've established a quantum inverse virtual screening (QIVS) platform (see Supplementary Figure 22). Inverse virtual screening (IVS) pinpoints potential drug targets for specific active molecules through calculations, aiding in target identification, side effect research, and drug repurposing [28, 29]. The IVS method demands significant computational resources due to extensive protein screening. Our approach diverges from traditional IVS by using GBS, resulting in a streamlined and precise QIVS. Based on the selected ligand and proteins, a BIG, $\mathcal{G}_{\text{BIG}}$, is formed (also in Supplementary Figure 22), encoded into the GBS machine, with the optimal binding pose determined by this graph's maximum weighted clique [25].

Details about the molecules used in experiments

In this section, we show the details about the molecules we analyze in our experiments. Here, we analyze the binding interaction of two pairs of protein-ligand complexes. The first experiment is based on the structure of the tumour necrosis factor (TNF)-α converting enzyme (TACE), which is also studied as the example in Banchi's experiment [25], complexed with a thiomorpholine sulfonamide hydroxamate inhibitor [30]; and the second one investigates the molecular binding of an 8-chloroquinazolinone-based ligand to the protein poly (ADP-Ribose) polymerase-1 (PARP1) [32].

Supplementary Figure 23 shows the pharmacophore potential we applied in experiment. We use same data as in Ref. [25], and these experience parameters are derived from PDBbind dataset [33–35].

A. The PARP1-inhibitor complex (corresponding to Fig. 2a)

The second experiment is based on the complex of PARP1 and an 8-chloroquinazolinone-based inhibitor (as shown in Supplementary Figure 24) [32].

As the most abundant member of the PARP family, PARP1 is responsible for numerous cellular activities such as DNA repair, inflammation, cell apoptosis and death, etc. [36]. As the competitive inhibitor, the PARP inhibitor

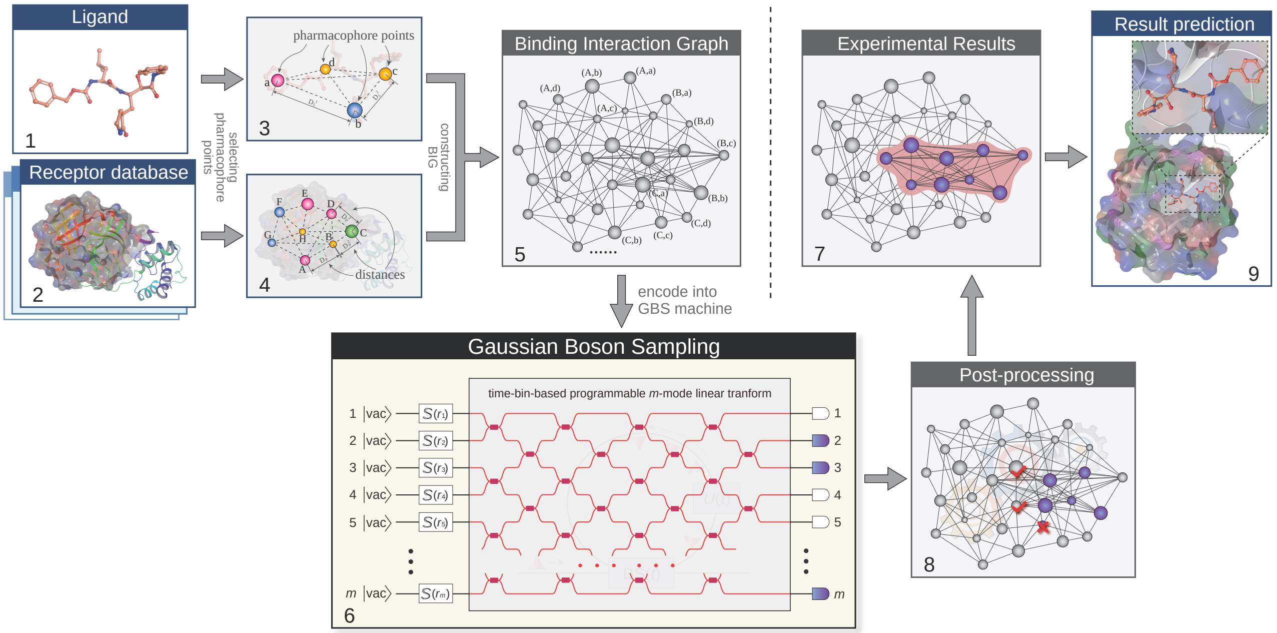

Supplementary Figure 22: **Schematic of the quantum inverse virtual screening platform**. The queried ligand provided by users and the proteins from the database occupy m and n considered pharmacophore points, respectively (the colors of points (a,...,d) and (A,...,H) denote different types of pharmacophore). The pharmacophore pairs (X,x) where X and x denote the pharmacophores from ligand and protein, respectively. All the pairs (X,x) are used to constitute the $(n \times m)$-node BIG, and whether the two nodes in the BIG are connected depends on the distance (D^i_L and D^i_P) between the corresponding pharmacophores (see Methods). The obtained BIG is then encoded on the programmable GBS machine, and the sampling results are used to find cliques of BIG. After post-processing of shrink and local search [25], the maximum weighted clique can be found, and the corresponding binding pose is then obtained. Finally, we can rank the different docked-proteins by protein-ligand interaction estimation giving us the solution.

Pharmacophore type	NC	PC	HD	HA	Hp	Ar
NC	0.2953	0.6459	0.7114	0.6450	0.1802	0.0000
PC	0.6459	0.1596	0.4781	0.7029	0.0679	0.1555
HD	0.7114	0.4781	0.5244	0.6686	0.1453	0.1091
HA	0.6450	0.7029	0.6686	0.5478	0.2317	0.0770
Hp	0.1802	0.0679	0.1453	0.2317	0.0504	0.0795
Ar	0.0000	0.1555	0.1091	0.0770	0.0795	0.1943

Supplementary Figure 23: **Pharmacophore potential**. Data is obtained from Ref. [33–35]. NC: negative charge; PC: positive charge; HD: hydrogen-bond donor; HA: hydrogen-bond acceptor; Hp: hydrophobe; Ar: aromatic.

(PARPi) mimics the protein's endogenous substrate NAD^+ and binds to the conserved nicotinamide binding site (NI) [37]. It is promising to use the PARPi as an anti-cancer drug. It could be used collectively with other anti-cancer therapies that cause DNA breaks, e.g., chemotherapy and radiotherapy [38–40]. It could also be used individually by BRCA-deficient patients with breast/ovarian cancers due to synthetic lethality [41]. Some studies show that PARPi may treat central nervous system (CNS) diseases such as Parkinson's disease and Alzheimer's disease as well probably due to its role in the inflammation process and its ability to stop NAD+ depletion in the CNS [42, 43]. The protein-ligand interaction between the PARP protein and a PARPi, especially in the NI, is well studied and we can easily

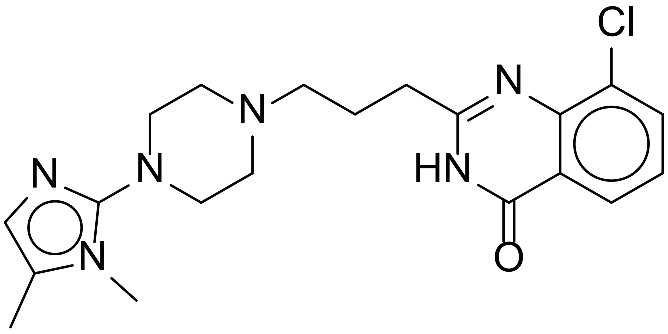

Supplementary Figure 24: **Structure of poly (ADP-Ribose) polymerase-1 inhibitor**.

a

Ligand:

	O5	N3	O1	N2
	hd1	hd2	ha1	hd3
hd1	0.000	1.400	5.660	11.49
hd2	1.400	0.000	4.370	10.94
ha1	5.660	4.370	0.000	8.440
hd3	11.49	10.94	8.440	0.000

Protein:

	GluOE1	GluOE2	Gly349O	Gly349N	Leu348N	Glu398OE2	Val440O
	HA1	NC1	HA2	HD1	HD2	NC2	HA3
HA1	0.000	2.170	4.230	5.640	8.010	14.10	12.77
NC1	2.170	0.000	3.240	3.970	6.090	12.05	10.96
HA2	4.230	3.240	0.000	2.720	4.610	12.94	12.62
HD1	5.640	3.970	2.720	0.000	2.780	10.75	11.25
HD2	8.010	6.090	4.610	2.780	0.000	9.420	10.39
NC2	14.10	12.05	12.94	10.75	9.420	0.000	4.970
HA3	12.77	10.96	12.62	11.25	10.39	4.970	0.000

b

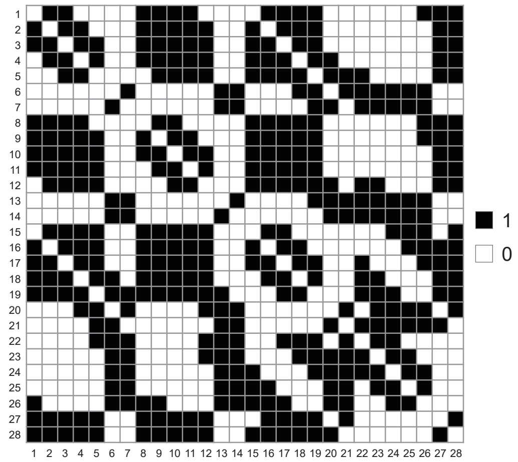

Supplementary Figure 25: **Distance data of the molecule and adjacency matrix of the graph of crystal structure of catalytic domain of TACE with a thiomorpholine sulfonamide hydroxamate inhibitor**.

identify the protein's and the ligand's pharmacophores. Important residues in the PARP1 protein include Gly-863, Ser-904 and Tyr-907 in the NI and provide pharmacophores including hydrogen bond acceptor (HA), hydrogen bond donor (HD) and $\Pi - \Pi$ interaction component (Ar). The benzamide analog is the most important group in a PARPi. In the benzamide, the carboxamide moiety is responsible for accepting and donating hydrogens interacting with Gly-863, Ser-904, and the aromatic component interacts with Tyr-907 through p-p interaction [44]. The PARP-inhibitor complex is also chosen due to the geometry of its active site. The cleft of the conserved binding site NI and the ligand fragment inside the NI are planar while the overall conformation of the binding pocket and the ligand are not. This experiment on the PARP-inhibitor complex will be a step forward compared to previous study [25].

Supplementary Figure 25(a) shows the detailed information about the distance data of the ligand (8-

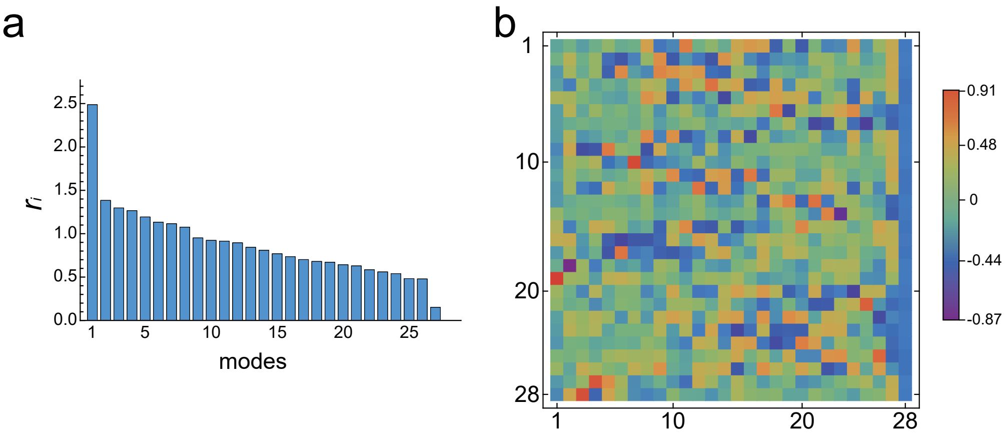

Supplementary Figure 26: **Squeezing parameters and unitary operation used in Fig. 2(a) experiments**.

chloroquinazolinone-based) and protein (PARP1), and Supplementary Figure 25(b) denotes the 28-mode adjacency matrix constructed according to the distance information of the molecular pair. Here, we choose $\tau = 3.5$ and $\varepsilon = 0.8$, and if $d_P < d_L + \tau$ (d_L and d_P represent the distance values shown in Supplementary Figure 25(a)), we set the corresponding element as 1, otherwise, we set the corresponding element as 0. At the end, we set all the diagonal elements as 0. For example, the mode numbers $1, 2, \cdots, 28$ in written in Supplementary Figure 25(b) denote the interaction pairs (hd_1-HA_1,hd_1-NC_1,hd_1-HA_2,hd_1-HD_1,$\cdots$,hd_3-HA_3). Thus, the element of adjacency matrix in first column and second row denotes the interaction pair [(hd_1-HA_1)-(hd_1-NC_1)]. Since $d_L = 0$ and $d_p = 2.17$, and of course we can see $2.17 < 0 + 3.5 + 2 \times 0.8$, we set this element as 1. At the same time, for the element of adjacency matrix in first column and fourth row (i.e., the interaction pair [(hd_1-HA_1)-(hd_1-HD_1)]), we will set it as 0 since $d_L = 0$ and $d_p = 5.64$, and $5.64 > 0 + 3.5 + 2 \times 0.8$.

Supplementary Figure 26(a) and (b) show the squeezing we used in experiments after a suitable rescaling and the unitary matrix (all the elements are real here), respectively.

Comparison with the results obtained from Schrodinger and MOE:
The complex file PDB ID 2A8H was fetched by Schrodinger Maestro and the protein and the ligand was saved as different entries. Solvent molecules were deleted. The protein was prepared using Protein Preparation Wizard with default settings. A grid for ligand binding site was generated according to default settings around the centroid of selected protein pharmacophores Leu348, Gly349, Glu398, Glu406 and Val440, of which the length is 20 Å. The ligand was prepared using LigPrep with default settings. The generated ligands were docked into the protein using Ligand Docking with default settings with XP (extra precision). Four ligands with different conformations and charges were generated and docked into the protein. The highest-scored docking result was taken as the docking result from Schrodinger Maestro. The 3D structure Supplementary Figure 27(a) below is obtained from Schrodinger package.

The complex file PDB ID 2A8H was imported to MOE excluding solvent molecules. The protein and the ligand were prepared by Structure Preparation respectively with default settings. The ligand was docked into the protein with the default settings and the site was determined by selected residues, i.e., the protein pharmacophores. Five docking results were generated and the highest-scored one was chosen as the docking result from MOE, and the corresponding 3D structure is shown in Supplementary Figure 27(b).

Supplementary Table 2 presents the highest-scoring docking result obtained from Schrodinger Maestro and MOE. By sorting these contacts based on their distances, we can identify the most probable contacts by considering only those within a distance of 4. Additionally, we sort the contacts obtained from our GBS experiments based on their weights, which correspond to their respective pharmacophore potentials shown in Supplementary Figure 23.

Here, we evaluate the quality of our GBS-based molecular docking results using precision and compare it with the commercialized software Schrodinger and MOE. Precision is calculated as the ratio of correctly classified positive samples to the total number of samples classified as positive (TP/(TP+FP)). In this case, the precision of our GBS result is 0.900 (TP=9, FP=1), while Schrodinger and MOE achieve 0.667 (TP=6, FP=3) and 0.909 (TP=10, FP=1) respectively. Our GBS method demonstrates accuracy similar to MOE and superior to Schrodinger.

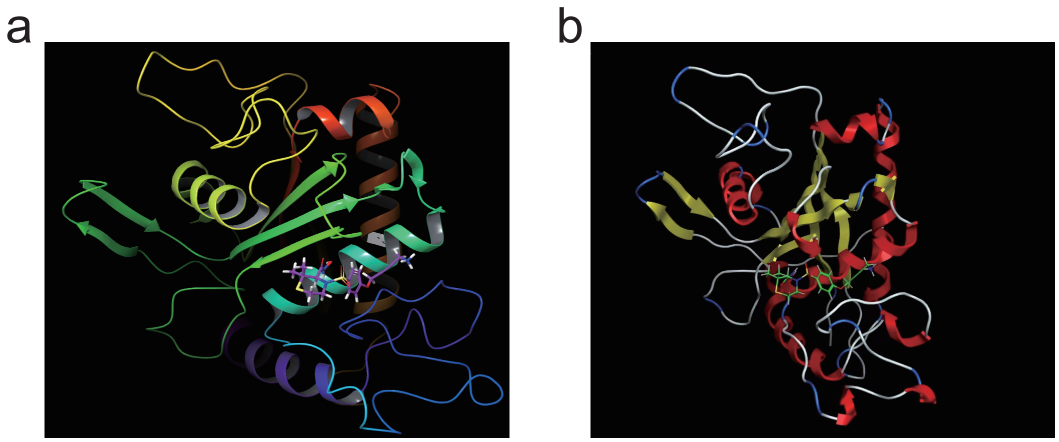

Supplementary Figure 27: **Docking results obtained from Schrodinger and MOE**.

**Original structure:**		**Schrodinger results:**		**MOE results:**		**GBS results:**	
contacts	distance	contacts	weighted	contacts	distance	contacts	weighted
HD2, HA1	2.69	NC1, HD1	2.59	NC1, HD1	2.81	NC1, HD1	0.7114
HA3, HD3	2.69	NC2, HD3	2.76	HA1, HD1	2.92	NC1, HD2	0.7114
HA1, HD1	2.99	HA3, HD3	2.78	NC2, HD3	2.94	NC2, HD3	0.7114
NC2, HD3	3.03	NC1, HD2	2.92	HA3, HD3	2.98	HA1, HD1	0.6686
NC1, HD2	3.10	HD1, HD1	3.13	HA2, HD2	3.64	HA1, HD2	0.6686
NC1, HD1	3.15	HA2, HD2	3.28	HD2, HA1	3.65	HA2, HD2	0.6686
HA2, HD2	3.19	HA2, HD1	3.41	NC1, HD2	3.70	HD1, HA1	0.6686
HD1, HA1	3.61	HD1, HD2	3.96	HA2, HA1	3.99	HD2, HA1	0.6686
HA1, HD2	3.75	HD2, HA1	3.99	HA2, HD1	3.63	HA3, HD3	0.6686
HA2, HA1	3.89	distance=4 cut-off		HD1, HA1	3.69	HA2, HD1	0.6686
distance=4 cut-off		HD2, HD1	4.10	HA1, HD2	3.96		
HA2, HD1	4.09	HA1, HD2	4.67	distance=4 cut-off			
NC1, HA1	5.12	HA1, HD1	4.70	NC1, HA1	4.86		
HD1, HD2	5.18	HD2, HD2	4.70	HD1, HD2	4.99		
HD1, HD1	6.06	HD1, HA1	5.64	HD1, HD1	5.64		
HD2, HD2	6.42	HA2, HA1	5.97	HD2, HD2	6.15		
HA1, HA1	6.96	NC1, HA1	6.79	HA1, HA1	6.64		
HD2, HD1	7.60	HA1, HA1	8.66	HD2, HD1	7.20		

Supplementary Table 2: **Results comparison between Schrodinger and GBS**.

B. The TACE-inhibitor complex (corresponding to Fig. 2b)

TACE is complexed with a thiomorpholine sulfonamide hydroxamate inhibitor, as shown in Supplementary Figure 28 [30]. The inhibitor shares a similar structure to the aryl-sulfonamide compound in the previous study while delivering a strong inhibition ability against TACE. As stated in the Banchi's study [25], TACE is chosen for its planar geometry of the active site cleft [31]. Meanwhile, the aryl-sulfonamide compound is chosen as it shares similar molecular binding pattern to Banchi's ligand (RMSD = 0.7 Å as calculated by Schrodinger Maestro while aligning the binding pocket, as shown in Supplementary Figure 23). Thus, the experiment is designed for verification and comparison purposes.

In experiments, we find that the accuracy of the docking results is likely influenced by pharmacophore points we choose. Using the same method as introduced in Ref. [25], higher accuracy docking results can always be found if only hydrogen-bond acceptor/donor, negative charge, or hydrophobe are considered. However, incorrect results will appear when aromatic (Ar) pharmacophore points are included.

This is close related to how the BIG is constructed. In Ref. [25], there are two assumptions under the judgement

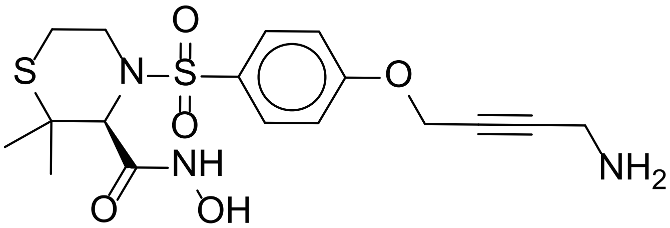

Supplementary Figure 28: **Structure of thiomorpholine sulfonamide hydroxamate inhibitor**.

$s_2 \leqslant s_1 + \tau + 2\varepsilon$:
1. each interaction taking place within a certain distance range;
2. the distances for different interaction to form are mostly identical.

Actually, the first assumption is true, but the second one is not suitable in general cases. In another word, the principle assumes, for example, a hydrogen bond is as same in length as a $\pi - \pi$ interaction. However, the distance between two functional groups forming a $\pi - \pi$ interaction, as a weaker interaction, could be greater than the distance between two functional groups forming a hydrogen bond, as a stronger interaction [45, 46].

a

Ligand:

	ha1 (O)	hd1 (N4)	ha2 (N)	ar1 (N4)
ha1	0.000	2.260	12.19	2.690
hd1	2.260	0.000	10.47	1.380
ha2	12.19	10.47	0.000	11.05
ar1	2.690	1.380	11.05	0.000

Protein:

	HA1 (G863)	HD1 (G863)	HD2 (S904)	HD3 (D770)	HA2 (D766)	Ar1 (Y907)
HA1	0.000	2.780	6.010	8.890	10.97	5.810
HD1	2.780	0.000	3.940	10.93	13.45	6.940
HD2	6.010	3.940	0.000	14.48	16.57	6.860
HD3	8.890	10.93	14.48	0.000	4.300	12.98
HA2	10.97	13.45	16.57	4.300	0.000	13.33
Ar1	5.810	6.940	6.860	12.98	13.33	0.000

b

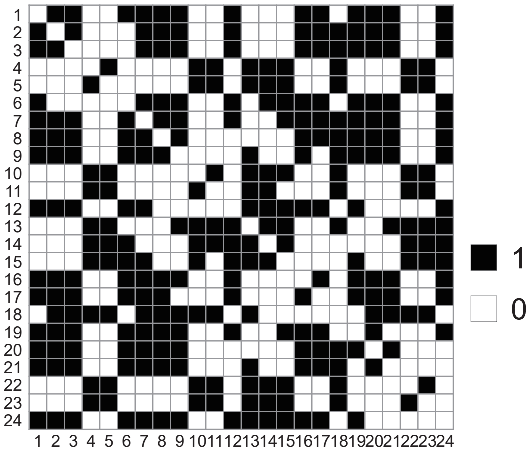

Supplementary Figure 29: **Distance data of the molecule and adjacency matrix of the graph of PARP1 catalytic domain in complex with an 8-chloroquinazolinone-based inhibitor.**

In order to fix this problem, we can use several separated interaction distances (ε) in the distance comparison judgement, and a more accurate result can be obtained when pharmacophore point (as shown in Supplementary Figure 31), for example, aromatic pharmacophore points (Ar), is considered in the docking process.

In Ref. [25], the constants τ and ε are set for simplicity, as discussed above. However, more accurate methods exist that can consider the variable flexibility of the ligand and receptor. Therefore, the changeable interaction distance feature selection method was introduced here, allowing exploration of a broader conformational space and potential

capture of the correct binding interaction and pose.

It is widely recognized that crystal structures do not provide exact representations of protein-ligand complexes, as the resulting structures can vary among different crystals. This variation is evident in the PARP1-Olaparib complex (PDB 5DS3 and 7AAD), where the distances from Arg878 to the piperazine ring differ. As a result, we observed varying flexibility among different pharmacophore points, leading us to conclude that $(\tau + 2\varepsilon)$ should not be treated as constants but rather allowed to vary based on the specific pharmacophore points involved. For instance, in our experiments, we noted distinct flexibility in the interaction distances of hydrogen-bonds and aromatics compared to other points. Therefore, unlike the fixed $(\tau + 2\varepsilon)$ in Ref. [25], we assigned different flexibilities $(\tau + 2\varepsilon)$ to different types of pharmacophore points. For example, we set flexibility as $(\tau + 2\varepsilon)$ between hydrogen-bonds and as $(\tau' + 2\varepsilon')$ between aromatics. The flexibility values chosen in Ref. [25] were based on experience and experimentation. In our work, we initially made empirical choices guided by the values used in Banchi et al. [25], setting $\tau = 3.5$, and $\varepsilon = 1.2$. Additionally, we provided a reasonable range of variation (i.e., $\varepsilon \to \varepsilon \pm \omega$) for different pharmacophore points based on our experience. In our experiments, we selected specific ranges of variation for different contact types: the variation of ε is set as [1.2-0.2, 1.2+0.2], [1.2-0.5, 1.2+0.5], [1.2-0.2, 1.2+0.2] for HA/HD-ha/hd, AR-ha/hd (or HA/HD-Ar), AR-Ar, respectively. These ranges were chosen based on empirical considerations, and it's important to note that the choice of ω is not unique. We thoroughly explored the full range of flexibility with an accuracy of 0.05, and based on the co-crystal structure information, we selected optimal values of ε as 1.30, 0.75, and 1.25 for HA/HD-ha/hd, AR-ha/hd (or HA/HD-Ar), and AR-Ar, respectively.

When the correct answer for molecular docking is unknown, the evaluation of proper binding interactions and poses can be achieved through the assessment of their scoring functions, such as binding energies. Docking programs often employ scoring functions that incorporate various energy terms, including van der Waals interactions, electrostatic interactions, hydrogen bonding, and solvation effects, to estimate the binding affinity between the ligand and the protein.

The researchers can systematically traverse the flexibility of these pharmacophore points within a certain range and choose a binding pose with high score. Then, the likelihood of identifying the correct binding interaction and pose can be increased. However, it is important to note that when utilizing this method or even employing mature docking software to identify the best scenarios, researchers still need to conduct wet laboratory experiments for validation. Comparison with a positive control group can be performed if available.

Overall, when the correct answer for molecular docking is unknown, a combination of consideration of ligand and protein changeable flexibility, careful algorithm selection, optimization of scoring functions, exploration of ensemble docking approaches, and integration of experimental data is necessary to increase the likelihood of selecting the changeable interaction distance feature and obtaining the proper binding interaction and pose.

Supplementary Figure 29(a) shows the detailed information about the distance data of the ligand (thiomorpholine sulfonamide hydroxamate inhibitor) and protein (TACE), and Supplementary Figure 29(b) denotes the corresponding 24-mode adjacency matrix. Here, τ is set as 3.5, ε is set as 1.30, 0.75, 1.25 for HA/HD-ha/hd, AR-ha/hd (or HA/HD-ar), AR-ar, respectively.

Supplementary Figure 30(a) and (b) show the squeezing we used in experiments after a suitable rescaling and the unitary matrix (all the elements are real here), respectively.

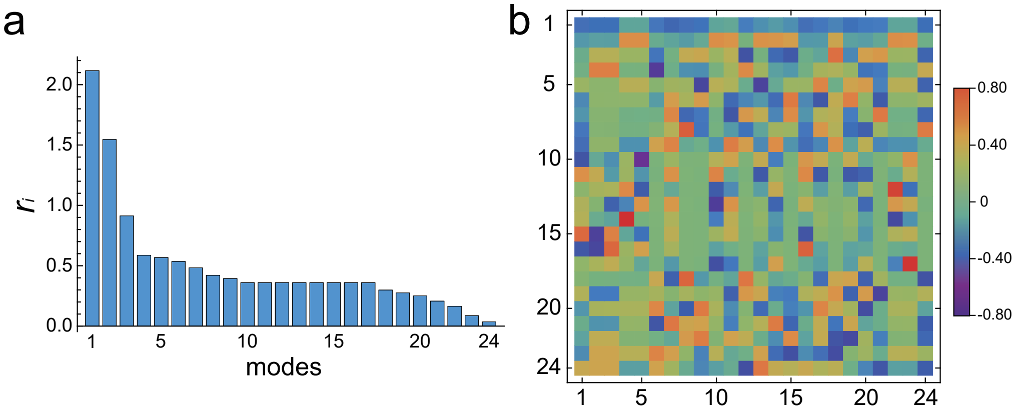

Supplementary Figure 30: **Squeezing parameters and unitary operation used in Fig. 2(b) experiments**.

The outcomes under whether the changeable interaction distances feature is considered are shown in Supplementary Figure 31. Three cases are analysis here as examples, and we find the best binding result only appears when the

changeable interaction distances feature is considered.

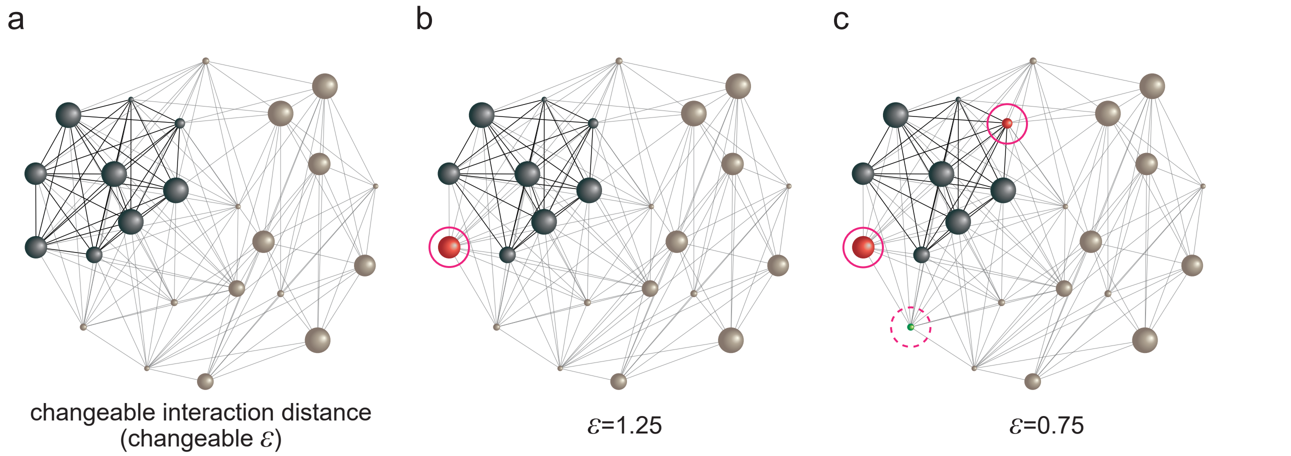

Supplementary Figure 31: **Comparison of experimental results with or without considering changeable** ε. **a,** The parameter ε is set as we discussed above. **b,** ε is set as 1.25 constantly. **c,** ε is set as 0.75 constantly. We can see in the cases shown in **b** and **c**, some connections becomes incorrect (the solid pink circle denote the corresponding interaction pair is missing, and the dashed pink circle denote the corresponding interaction pair is surplus).

Comparison with the results obtained from Schrodinger and MOE:
The complex file with PDB ID 7ONR was obtained using Schrodinger Maestro, and the protein and ligand were saved as separate entries. Solvent molecules were removed. The protein was prepared using the Protein Preparation Wizard with default settings. A grid for the ligand binding site was generated around the centroid of selected protein pharmacophores Gly863, Ser904, Asp770, and Tyr907, with a length of 20 Å, using default settings. The ligand was prepared using LigPrep with default settings. The generated ligands were docked into the protein using Ligand Docking with default settings and extra precision. Four ligands with different conformations and charges were generated and docked into the protein. The highest-scoring docking result obtained from Schrodinger Maestro was considered as the final docking result. The 3D structure Supplementary Figure 32(a) below is obtained from Schrodinger package. The complex file with PDB ID 7ONR was imported into MOE, excluding solvent molecules. The protein and the ligand were separately prepared using the Structure Preparation tool with default settings. The ligand was then docked into the protein using the default settings, and the binding site was determined based on selected residues, specifically the protein pharmacophores. Five docking results were generated, and the highest-scoring result was selected as the final docking result obtained from MOE, and the corresponding 3D structure is shown in Supplementary Figure 32(b).

Supplementary Table 3 presents the highest-scoring docking result obtained from Schrodinger Maestro and MOE. We also sort the contacts obtained from our GBS experiments based on their weights.

We observe that the GBS results ($precision$=0.889) show remarkable similarity to the original structure and Schrodinger results ($precision$=1.00) for contacts with higher weights. The only discrepancy occurs in the contact [HA1, Ar1], which results in a larger distance in the original structure and Schrodinger. Additionally, the GBS results align well with those obtained from MOE ($precision$=0.875), except for two contacts [HD1, HD1] and [HA1, Ar1]. These findings underscore the effectiveness of the GBS-based macromolecular docking method.

$*$**Comparing the clique-finding results with classical Bron-Kerbosch algorithm and Maximum Clique algorithm**

During the experimental process, we make modifications to the graphs (i.e., re-scaling the corresponding adjacency matrix $A = cB + d\mathbb{I}$) to align them with the system. However, these adjustments were primarily performed to ensure the successful encoding of the graph's adjacency matrix A in the GBS machine and to detect a higher number of photons at the output. It is important to note that such modifications do not impact the sampling results, specifically the selection of cliques.

To be more specific, any matrix B with spectra in the range $[0, 1]$ can be encoded into the GBS machine. However, the adjacency matrix A of a graph typically lacks this spectral property. Fortunately, it can always be re-scaled as $A = cB + d\mathbb{I}$, where B possesses the desired spectra and c, d are appropriate re-scaling constants. By leveraging the Hafnian property, namely $\mathrm{Haf}(A) = c^N \mathrm{Haf}(B)$, and $\mathrm{Haf}(B \oplus B^*) = |\mathrm{Haf}(B)|^2$, we have discovered that the GBS device can be programmed to sample from the probability distribution $p(S) \sim |\mathrm{Haf}(A_S)|^2/c^N$ (where N represents

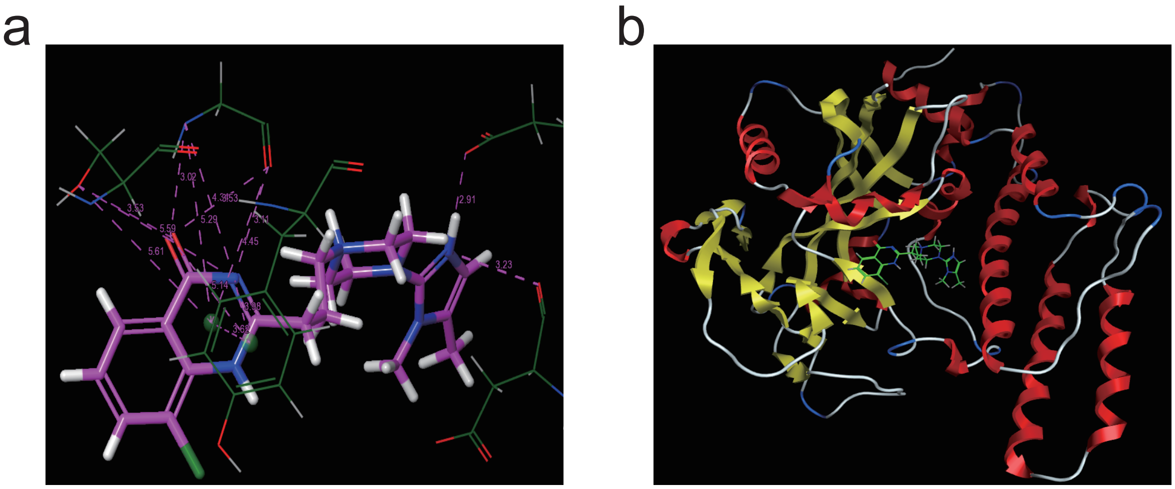

Supplementary Figure 32: **Docking results obtained from Schrodinger and MOE**.

**Original structure:**		**Schrodinger results:**		**MOE results:**		**GBS results:**	
contacts	distance	contacts	weighted	contacts	distance	contacts	weighted
HA1, HD1	2.85	HD1, HA1	2.82	HD3, HA2	2.91	HD1, HA1	0.6686
HD2, HA1	2.88	HD2, HA1	2.92	HD1, HA1	3.02	HD2, HA1	0.6686
HD3, HA2	2.90	HD3, HA2	2.94	HA1, HD1	3.11	HD3, HA2	0.6686
HD1, HA1	2.92	HA1, HD1	2.98	HA2, HA2	3.23	HA1, HD1	0.6686
HA2, HA2	3.30	HA1, HA1	3.64	HA1, HA1	3.53	HA1, HA1	0.5478
Ar1, Ar1	3.72	Ar1, Ar1	3.65	HD2, HA1	3.53	HA2, HA2	0.5478
HD1, HD1	3.80	HA2, HA2	3.70	Ar1, Ar1	3.62	HD1, HD1	0.5244
HA1, HA1	3.84	HD1, HD1	3.99	Ar1, HD1	3.98	Ar1, Ar1	0.1943
distance=4 cut-off		distance=4 cut-off		distance=4 cut-off		HA1, Ar1	0.0770
Ar1, HD1	4.19	Ar1, HD1	4.01	HD1, HD1	4.34		
HA1, Ar1	4.22	HA1, Ar1	4.34	HA1, Ar1	4.45		
HD1, Ar1	4.96	Ar1, HA1	5.04	Ar1, HA1	5.14		
HD2, HA1	5.04	HD1, Ar1	5.05	HD1, Ar1	5.29		
HD2, Ar1	5.27	HD2, HD1	5.15	HD2, HD1	5.59		
Ar1, HA1	5.29	HD2, Ar1	5.26	HD2, Ar1	5.61		

Supplementary Table 3: **Results comparison between Original, Schrodinger, MOE and GBS results**.

the number of modes and S denotes the sampling patterns) [25].

This mathematical relationship clearly demonstrates that the modification (i.e., scaling operation) of the graph's adjacency matrix A solely results in an overall scaling of probabilities, without altering the sampling distribution. In other words, it does not impact the results of clique-finding. Consequently, the graph modification ensures the correctness of solving the clique-finding problem.

To validate the accuracy of our results, we applied several widely used graph optimization methods, such as Bron-Kerbosch algorithm and Maximum Clique algorithm, to analyse the same system for clique-finding. We find that the results obtained using these mature classical graph optimization algorithms are exactly the same as those obtained using GBS experiments, and the related classical algorithms can be found with this paper.

VI. DETAILS ABOUT THE MOLECULAR DOCKING EXPERIMENTAL RESULTS

In this section, we show all the results (with their weight and probabilities) of the binding pose indicated by cliques which are found in the GBS experiments.

Supplementary Figure 33 shows the results of the cliques found in the 28-node graph constructed by PARP-CQ complex. After 100 local search iterations of 347 effective sampling results (i.e., sampling results with four or more photon events), we get totally 11 groups of cliques, and there are 4 10-vertex cliques among them. The optimal binding pose of this complex is the first one with maximum weight 6.8144, and highest proportion 24.78% among them.

No.	Outcomes	Proportion	Weight
1	(HA1, hd1) (NC1, hd1) (HA2, hd1) (HA1, hd2) (NC1, hd2) (HA2, hd2) (HD1, ha1) (HD2, ha1) (NC2, hd3) (HA3 hd3)	31.71%	6.8144
2	(HA1, hd1) (NC1, hd1) (HA1, hd2) (NC1, hd2) (HA2, hd2) (HA2, ha1) (HD1, ha1) (HD2, ha1) (NC2, hd3) (HA3 hd3)	17.89%	6.6936
3	(HA1, hd1) (NC1, hd1) (HA2, hd1) (NC1, hd2) (HA2, hd2) (HD1, hd2) (HD1, ha1) (HD2, ha1) (NC2, hd3) (HA3 hd3)	17.07%	6.6702
4	(HA1, hd1) (NC1, hd1) (NC1, hd2) (HA2, hd2) (HD1, hd2) (HA2, ha1) (HD1, ha1) (HD2, ha1) (NC2, hd3) (HA3 hd3)	23.58%	6.5494
5	(HA2, hd1) (HD1, hd1) (HD2, hd1) (NC1, hd2) (HA2, hd2) (HD1, hd2) (NC1, ha1) (NC2, hd3) (HA3, ha3)	0.81%	5.6468
6	(NC2, hd1) (HA3, hd1) (NC2, hd2) (HD2, ha1) (HA1, hd3) (NC1, hd3) (HA2, hd3)	0.81%	4.8086
7	(NC2, hd1) (HA3, hd1) (NC2, hd2) (HA3, hd2) (NC1, hd3) (HA2, hd3) (HD1, hd3)	3.25%	4.6644
8	(NC2, hd1) (NC2, hd2) (HA3, hd2) (HA3, ha1) (NC1, hd3) (HA2, hd3) (HD1, hd3)	1.63%	4.5436
9	(NC2, hd2) (HA3, hd2) (NC2, ha1) (HA3, ha1) (NC1, hd3) (HA2, hd3) (HD1, hd3)	1.63%	4.4772
10	(HA3, hd1) (NC2, hd2) (HA3, hd2) (NC2, ha1) (HA2, hd3) (HD1, hd3) (ND2, hd3)	0.81%	4.4110
11	(NC2, hd1) (NC2, hd2) (HA3, hd2) (HA3, ha1) (HA2, hd3) (HD1, hd3) (ND2, hd3)	0.81%	4.3566

Supplementary Figure 33: **Detailed experimental results corresponding to Fig. 2(a)**.

Supplementary Figure 34 shows the results of the cliques found in the 24-node graph constructed by TACE-TS complex. After five local search iterations of 254 effective sampling results (in order to avoid the interference of errors, we only select three or more photon events as effective sampling data), we get four groups of cliques. The max weight cliques corresponds to the optimal binding pose of this complex, and it also accounts for the largest proportion of all cliques.

No.	Outcomes	Proportion	Weight
1	(HA1, ha1) (HD1, ha1) (HD2, ha1) (HA1, hd1) (HD1, hd1) (HD3, ha2) (HA2, ha2) (HA1, ar1) (Ar1, ar1)	18.50%	4.5657
2	(HA1, ha1) (HD1, ha1) (HD2, ha1) (HA1, hd1) (HD1, hd1) (HD2, hd1) (HD3, ha2) (HA1, ar1) (Ar1, ar1)	11.42%	4.5423
3	(HA1, ha1) (HD1, ha1) (HD2, ha1) (HA1, hd1) (HD1, hd1) (HD3, ha2) (HA2, ha2) (HD1, ar1) (HD2, ar1)	9.84%	4.5126
4	(HA1, ha1) (HD1, ha1) (HD2, ha1) (HA1, hd1) (HD1, hd1) (HD2, hd1) (HD3, ha2) (HD1, ar1) (HD2, ar1)	14.96%	4.4892
5	(HA1, ha1) (HD1, ha1) (HD2, ha1) (HA1, hd1) (HD1, hd1) (HD3, ha2) (HA2, ha2) (HA1, ar1) (HD1, ar1)	15.75%	4.4805
6	(HA1, ha1) (HD1, ha1) (HD2, ha1) (HA1, hd1) (HD1, hd1) (HD2, hd1) (HD3, ha2) (HA1, ar1) (HD1, ar1)	15.75%	4.4571
7	(HA1, ha1) (HD1, ha1) (HD2, ha1) (HA1, hd1) (Ar1, hd1) (HD3, ha2) (HA2, ha2) (HA1, ar1) (Ar1, ar1)	6.30%	4.1504
8	(HA1, ha1) (HD2, ha1) (HA1, hd1) (HD1, hd1) (HD2, hd1) (HD3, ha2) (HA1, ar1) (Ar1, ar1)	0.39%	3.8737
9	(HD3, ha1) (HA2,ha1) (HD3, hd1) (HA1, ha2) (HD1, ha2) (HD2, ha2) (HD3, ar1) (HA2, ar1)	3.15%	3.8119
10	(HD3, ha1) (HA2,ha1) (HD3, hd1) (HA2, hd1) (HA1, ha2) (HD1, ha2) (HD3, ar1) (HA2, ar1)	1.18%	3.8119
11	(HD3, ha1) (HA2,ha1) (HD3, hd1) (HA2, hd1) (HA1, ha2) (Ar1, ha2) (HD3, ar1) (HA2, ar1)	2.76%	3.2203

Supplementary Figure 34: **Detailed experimental results corresponding to Fig. 2(b)**.

We need to note that the shrink and local search methods introduced in Ref. [25] will give different results in different post-processing calculations while same experimental data are used. Namely, we find the largest proportion may not appear in the maximum weighted clique at sometimes even same experimental data are used. This is due to the randomness of local search in the post-processing program. The above phenomenon may also be caused by errors in the experiment, such as large loss exists in experiments. In any case, maximum weighted clique always accounts for a relatively large proportion of all results, which can enable us to quickly find the correct docking results even some imperfections exist.

VII. DETAILS ABOUT THE GBS-BASED RNA FOLDING PREDICTION

A stem graph, also known as a stem-loop diagram, is a useful tool for visualizing and analyzing the secondary structure of RNA molecules [47] and play an important role in RNA structure prediction and drug design. In our work, we utilize the method proposed in Ref. [47] to construct the full stem graph. Furthermore, we assign the length of each stem as the weight of its corresponding node. We then encode this weighted full stem graph (WFSG) into *Abacus* to efficiently find the maximum weighted cliques, which correspond to the RNA folding prediction information.

A. Example 1 (corresponding to Fig. 2c)

In this case, we use a tRNA fragment (accession number: AH003339) [48] to demonstrate the folding prediction ability of *Abacus*.

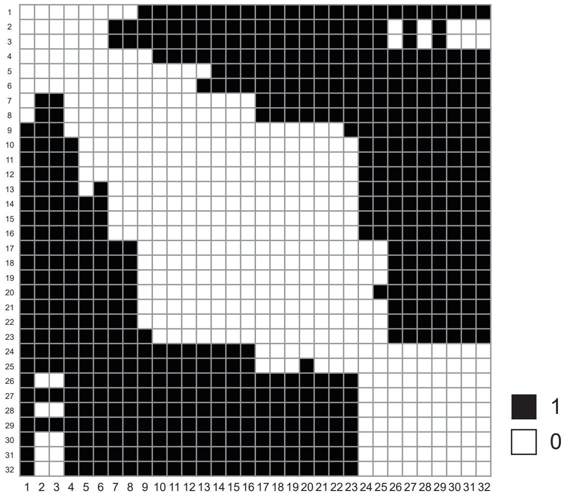

Supplementary Figure 35: **Adjacency matrix of tRNA fragment (accession number: AH003339) used in experiments**.

The RNA sequence here is:

$$\texttt{GGGGC CAUAG CUCAG UUGGU AGAGC GCCUG CUUUG CAAGC AGGUG UCGUC GGUUC GAAUC CGUCU GGCUC CACCA.} \tag{14}$$

Based on the sequence, we can construct a corresponding full stem graph via the method introduced in Ref. [47], and add the weights on each node according to the length of stem. The parameters for constructing WFSG are set as: minimum stem length is 3; lower bound and upper bound of stem-loop score are 3.0 and 5.4, respectively; minimum, maximum distance are 12 and 18, respectively; and $\texttt{G}-\texttt{U}$ pair is allowed here. The corresponding adjacency matrix is shown in Supplementary Figure 35. These settings also apply in Example 2.

Supplementary Figure 36 displays two possible predictions that are deduced from the sampling results, corresponding to the GBS-found cliques shown in Fig. 2(c) in the main text. We evaluated the accuracy of these predictions using the Matthews correlation coefficient (MCC), which is defined as $\text{MCC} = \sqrt{\text{Sens} \times \text{PPV}}$, where Sensitivity (Sens) $= TP/(TP + FN)$, Positive Prediction Value (PPV)$= TP/(TP + FP)$, and TP, FP, and FN respectively denote true positive (i.e., correct base-pairs matching), false positive (i.e., redundant base-pairs matching), and false negative

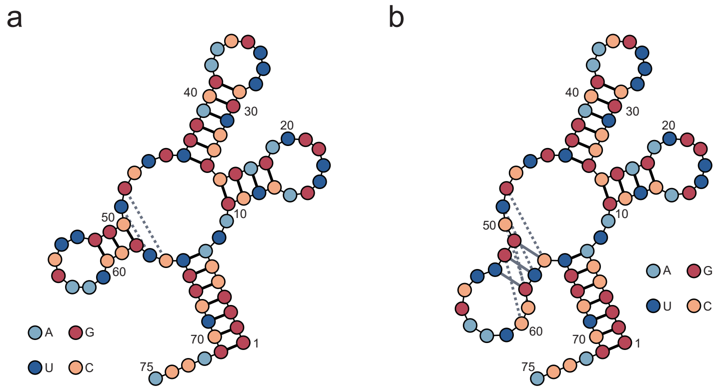

Supplementary Figure 36: **All tRNA sequences (in example 1) folding prediction results correspond to maximum cliques obtained from GBS experiments**. The four colored circles (A, G, U, C) in the RNA structure represent four different bases. The MCC values corresponding to the prediction in (a) and (b) are 0.953 and 0.810, respectively. The grey solid lines denote the false positive base-pair matching, and the grey dashed lines represent the false negative base-pair matching.

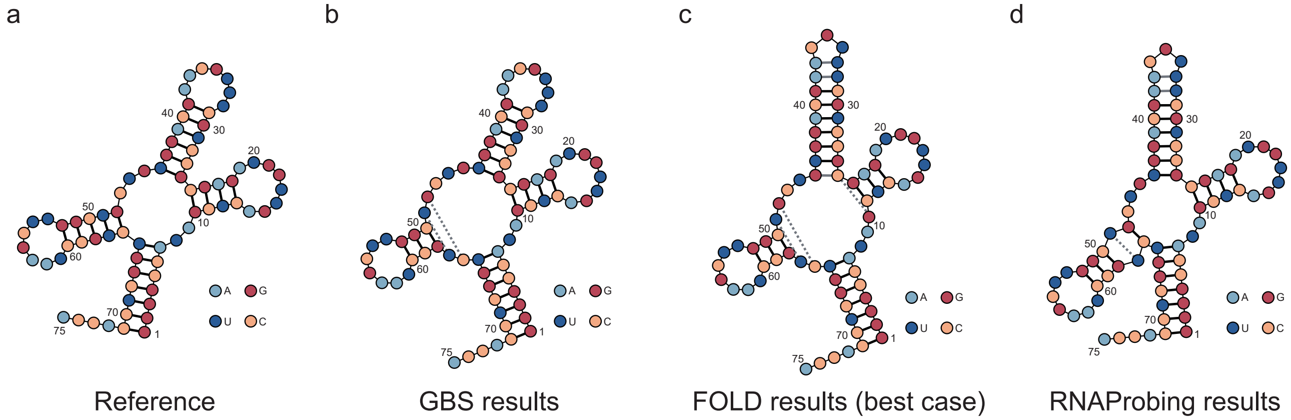

Supplementary Figure 37: **Comparing the predictions results**. The prediction results of RNA fragment (AH003339) using GBS, and FOLD and RNAProbing are compared with the reference structure. We want to note that the result of FOLD shown in **c** is the best one among the total three results (other two results found by FOLD with lower quality are not shown here).

(i.e., missed base-pairs matching) [47]. Comparing with the reference structure (shown in Supplementary Figure 37(a)), we obtained MCC values of 0.953 and 0.810 for the two folding predictions, respectively. In the main text, we choose to present the optimal prediction, i.e., the one shown in Supplementary Figure 36(a).

Supplementary Figure 37 shows the folding prediction outcomes with different tools. Comparing with the reference structure, we can see WFSG-based GBS method provides the best result with matthews MCC = 0.953 (with $TP = 20$, $FP = 0$, and $FN = 2$), while MaxExpect and FOLD only get MCC=0.857 (with $TP = 18$, $FP = 3$, $FN = 3$, and the results from MaxExpect and the best case from FOLD is same here). This shows our quantum folding predictor can give a more accurate result.

B. Example 2 (corresponding to Fig. 2d)

In this example, we use the tRNA fragment (accession number: AB041850) [49] and its sequence is:

$$\texttt{GUCCC GUUCG UCUAG AGGCC UAGGA CACCG CCCUU UCACG GCGGU AACAG GGGUU CGACU CCCCU ACGGG AUACC A.} \tag{15}$$

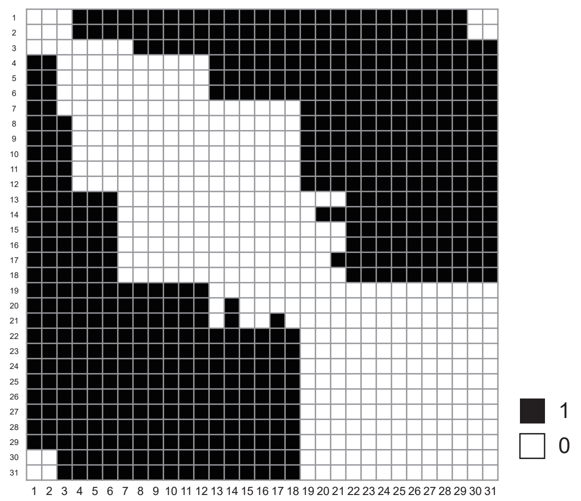

Supplementary Figure 38: **Adjacency matrix of tRNA fragment (accession number: AB041850) used in experiments**.

Its corresponding adjacency matrix is shown in Supplementary Figure 38.

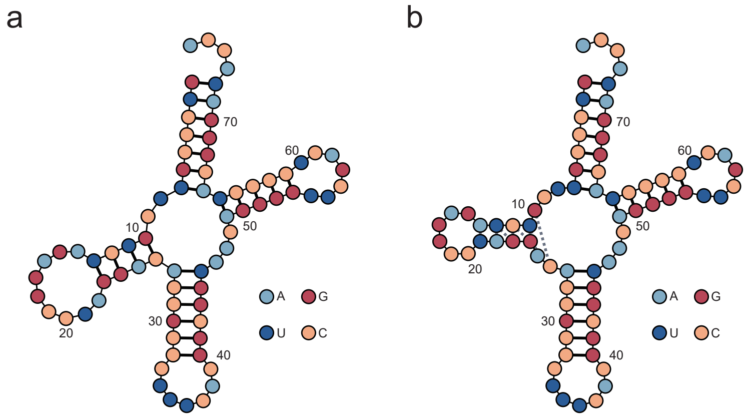

Supplementary Figure 39: **All tRNA sequences (in example 2) folding prediction results correspond to maximum cliques obtained from GBS experiments**. The MCC values corresponding to the prediction in (a) and (b) are 1.000 and 0.818, respectively. The grey solid lines denote the false positive base-pair matching, and the grey dashed lines represent the false negative base-pair matching.

Supplementary Figure 39 shows the GBS-found two results which corresponds to the two cliques shown in the Fig. 2(d). In the main text. we show the optimal prediction (i.e., the prediction shown in Supplementary Figure 39(a)).

We also compare the above optimal GBS result with other classical methods, and the results shown in Supplementary Figure 40. FOLD and RNAProbing only get MCC=0.870 (with $TP = 20$, $FP = 4$, and $FN = 2$) and 0.914 (with $TP = 21$, $FP = 3$, and $FN = 1$), respectively, while the best result comes from *Abacus* find the ideal prediction result.

VIII. FURTHER SCALING AND QUANTUM COMPUTATION ADVANTAGES OF OUR SETUP

Increasing the number of modes and the average number of photons can help to achieve quantum advantage in GBS experiments [50]. In order to perform GBS in the collision-free regime in our experiment, the average number of photon is limited guaranteeing that the cliques can be found accurately. Thus, scaling to a large number of modes becomes the most viable way of achieving quantum computational advantage.

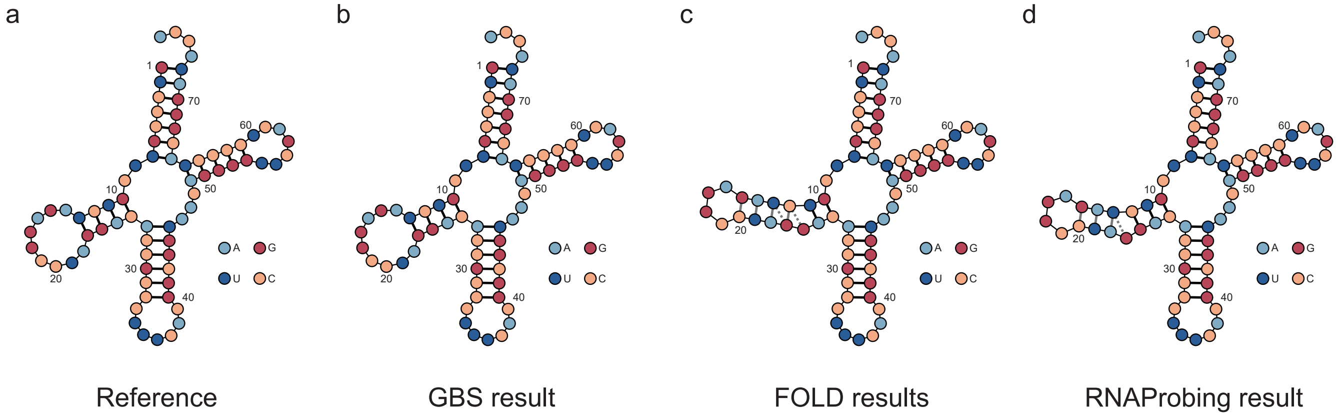

Supplementary Figure 40: **Comparing the predictions results**. The prediction results of RNA fragment (AB041850) using GBS, and FOLD and RNAProbing are compared with the reference structure.

To scale the number of modes whilst minimizing loss, we propose a "multi-core" encoding method. Inspired by the multi-core structure in classical central processing units (CPU), we can insert an additional "core" in our GBS device. The interferometer we built in this work can be regarded as one core. Additional cores can be inserted into the loop where each core has a different length delay line. In this section, we want to discuss how the number of mode to be scaled by this "multi-core" encoding method with the setup we built in this experiment. In order to maintain the universality feature in our GBS machine, we have to ensure the interferometer circuit is fully connected and has enough freely adjustable parameters (i.e., N^2 parameters, where N is the number of the modes) can be adjusted. Therefore, we will continue use the loop-based scheme, and combine it with the 'multi-core" structure, as shown in Supplementary Figure 41.

The circuit in blue denotes the linear transformation $T_{i,i+1}(\varphi,\theta)$ between the adjacency modes. They are in groups of ten, and there are totally 10 such groups in this 100-mode circuit. We make that these modes are processed in x-core, and can be denoted as $*0, *1, \ldots, *9$. Here $*$ in the first digit represents the pattern which will be processed by y-core, and the second digit represents the pattern which will be processed by x-core. If we regard QPU module in Fig. 1(a) as one core (QPU in Fig. 1(a) only has one core, which constitutes with EOM1, EOM2, and 7.5 m delay line), it can achieve $T_{i,i+1}(\varphi,\theta)$ (the blue part) between $0* \leftrightarrow 1*, 2* \leftrightarrow 3*, \ldots, 8* \leftrightarrow 9*$ modes in odd cycles (or between $1* \leftrightarrow 2*, 3* \leftrightarrow 4*, \ldots, 7* \leftrightarrow 8*$ modes in even cycles). Besides, the circuit in red denotes the linear transformation $T_{j,j+10}(\varphi,\theta)$ acted on modes $0* \leftrightarrow 1*, 2* \leftrightarrow 3*, \ldots, 8* \leftrightarrow 9*$ in odd cycles (or acted on modes $1* \leftrightarrow 2*, 3* \leftrightarrow 4*, \ldots, 7* \leftrightarrow 8*$ in even cycles). It has same architecture as the blue one, and we can add one more core and replace the delay line with a 10 times longer one to realize them.

The above "dual-core operation" constitutes a unit in the circular circuit, and the number of loops will be decided by whether we can have enough free parameters can be tuneable, and make the whole circuit fully connected. There are 100^2 free parameters that needs to be adjusted in this 100-mode time-bin interferometer, and in each unit, there are 100 (or 80 for even cycles) linear transformations in total, which introduces 200 (or 160 for even cycles) free parameters (including both real (θ), and image (φ) parameters). To achieve full programmability, $2 \times \lceil 100^2/((100+80)\times 2)\rceil = 56$ iterations needs to be applied (as shown in Supplementary Figure 41). At the same time, this number of iterations is also enough to ensure full connectivity of the circuit.

To further reduce the depth of circuit, we can slightly change our setup (i.e., the core) by putting EOM1 (for adjusting φ) before PBS and putting EOM2 (for adjusting θ) between the PBSs which are located at the delay line part (similar as Ref. [20, 51]), and achieve a more compact encoding. Then a specific circuit will be performed to ensure full connectivity and a sufficient number of tunable parameters of these 100 modes, as shown in Supplementary Figure 42:

1. A linear transformation $T(\theta,\varphi)$ will act on every adjacent mode in $x-$core, i.e., $[*0] \leftrightarrow [*1]$,$[*1] \leftrightarrow [*2]$,…,$[*8] \leftrightarrow [*9]$, which can be achieved by the slightly modified setup QPU module with a τ delay line. There are in total 90 $T(\theta,\varphi)$ operations in this 100-mode circuit.

2. Another linear transformation $T'(\theta,\varphi)$ will act on every adjacent mode in $y-$core (i.e., $[0*] \leftrightarrow [1*]$,$[1*] \leftrightarrow [2*]$,…,$[8*] \leftrightarrow [9*]$, and there are also 90 operations in total). This can be achieved by inserting another similar QPU module but with a 10τ delay line.

We can see there are totally 180 linear transformations (i.e., 360 free parameters can be adjusted) in every loops,

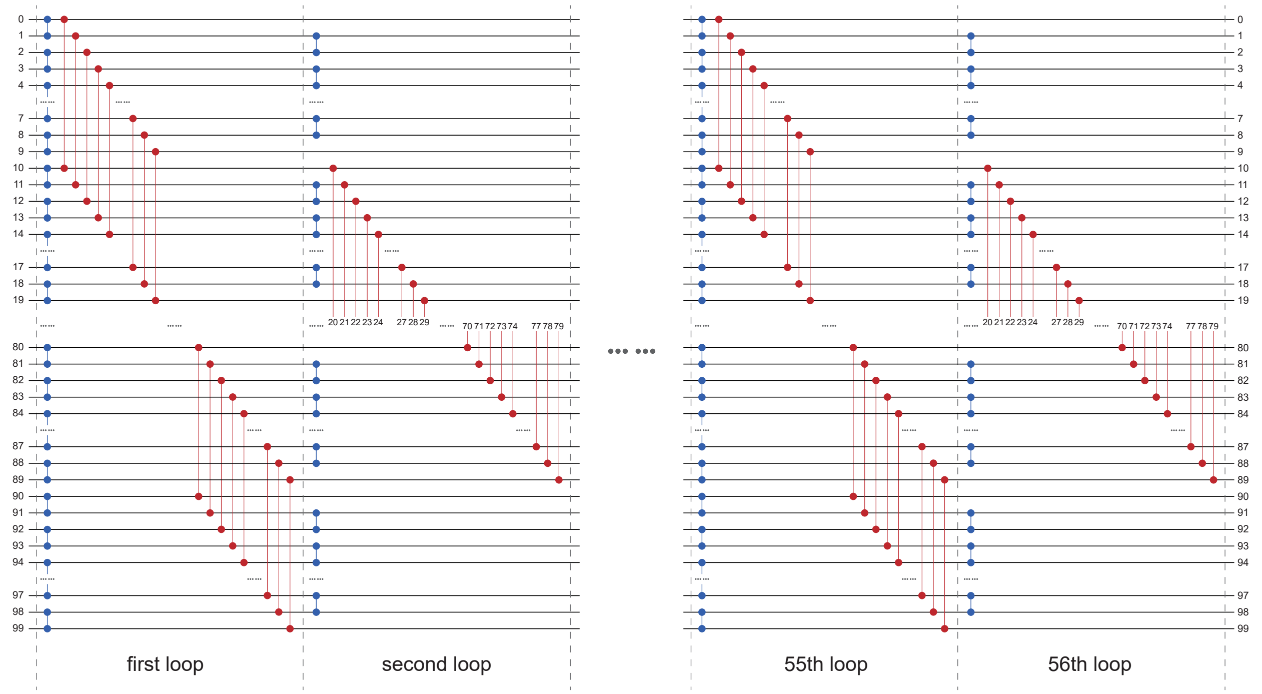

Supplementary Figure 41: **An example circuit of 100-mode GBS setup with multidimensional encoding architecture**.

and therefore, only $2 \times \lceil 100^2/180 \rceil = 28$ loops are needed to ensure the universality in this case. The corresponding experimental realization is shown in Supplementary Figure 43.

It is clearly that such a "multi-core" architecture can greatly reduce the depth of the circuit. A 100-mode full programmable interferometer needs 101 iterations (i.e., circuit depth, an additional iteration works for phase compensation) if we use the original Clements' architecture, but only 28 iterations can achieve that if we use this upgraded "multi-core" encoding method. This new architecture has laid a foundation for us to realize quantum advantage in our future works.

In order to provide a concrete and convincing proof that it can realize arbitrary unitary operation, we use a six-mode circuit here, and find all the parameters by simple annealing algorithm. The six-mode unitary operation is randomly selected:

$$U_{\text{target}} = \begin{pmatrix} 0.281075 + 0.432963i & 0.347705 - 0.325001i & 0.20674 - 0.490816i & 0.120311 - 0.0155808i & -0.17314 + 0.239625i & 0.279291 - 0.207979i \\ 0.152251 - 0.152428i, & 0.603675 + 0.336299i, & -0.435531 + 0.0461203i, & 0.134311 - 0.335874i, & 0.195416 + 0.131419i, & 0.222207 + 0.220374i \\ 0.413104 - 0.490277i, & 0.0785029 + 0.132735i, & 0.637615 + 0.0190317i, & -0.213425 - 0.00455166i, & 0.137261 + 0.290226i, & -0.044657 + 0.0874085i \\ -0.173144 + 0.073664i, & -0.275901 - 0.247104i, & 0.259585 + 0.109779i, & 0.565238 - 0.412949i, & 0.402607 + 0.152151i, & 0.140619 + 0.230089i \\ 0.217094 + 0.210293i, & 0.230053 + 0.163725i, & 0.138736 + 0.0882522i, & 0.267503 + 0.268241i, & 0.547823 - 0.394719i, & -0.27511 - 0.356049i \\ 0.338696 - 0.186079i, & -0.235051 - 0.00349251i, & -0.0684531 + 0.0804419i, & 0.203938 + 0.365702i, & -0.0541733 - 0.331636i, & 0.695272 + 0.112215i \end{pmatrix}. \tag{16}$$

According to the above analysis, a six-mode interferometer needs 36 adjustable parameters. The "multi-core" encoding architecture provides 14 adjustable parameters in each cycle, and that means 3 cycles are needed here (as shown in Supplementary Figure 44). Through annealing searching, we find all values of total 42 parameters:

The reconstructed unitary matrix is:

$$U_{\text{reconstructed}} = \begin{pmatrix} 0.281075 + 0.432963i & 0.347705 - 0.325001i & 0.206741 - 0.490816i & 0.120311 - 0.0155808i & -0.17314 + 0.239625i & 0.279291 - 0.207979i \\ 0.152251 - 0.152428i & 0.603675 + 0.336299i & -0.435531 + 0.0461203i & 0.134311 - 0.335874i & 0.195416 + 0.131419i & 0.222207 + 0.220374i \\ 0.413104 - 0.490277i & 0.0785029 + 0.132735i & 0.637615 + 0.0190316i & -0.213425 - 0.00455161i & 0.137261 + 0.290226i & -0.0446571 + 0.0874084i \\ -0.173144 + 0.073664i & -0.275901 - 0.247104i & 0.259585 + 0.109779i & 0.565238 - 0.412949i & 0.402607 + 0.152151i & 0.140619 + 0.230089i \\ 0.217094 + 0.210293i & 0.230053 + 0.163724i & 0.138736 + 0.0882522i & 0.267503 + 0.268241i & 0.547822 - 0.394719i & -0.27511 - 0.356049i \\ 0.338696 - 0.186079i & -0.235051 - 0.00349248i & -0.0684531 + 0.080442i & 0.203938 + 0.365702i & -0.0541733 - 0.331636i & 0.695272 + 0.112214i \end{pmatrix}, \tag{17}$$

and the error $\sqrt{\sum_{ij} |U_{\text{target}}(i,j) - U_{\text{exp.}}(i,j)|^2}$ is 4.913×10^{-7}. This shows our "multi-core" encoding architecture

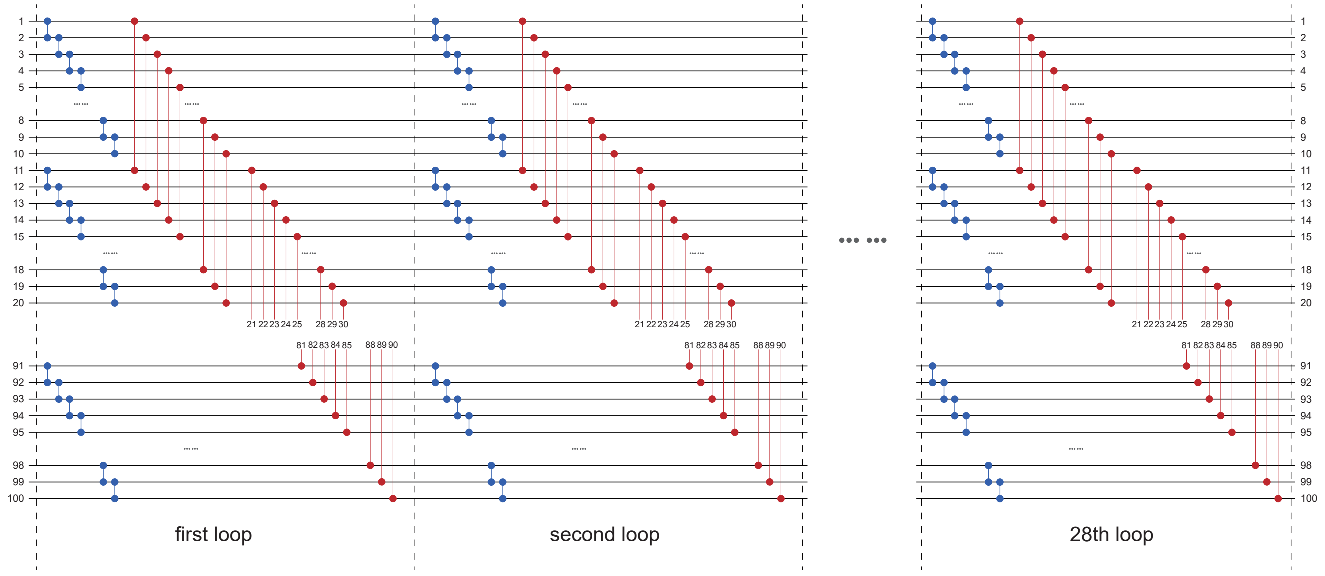

Supplementary Figure 42: **A more compact circuit for 100-mode GBS**. The blue circles and lines indicate the linear transformation $T(\theta,\varphi)$ act on every adjutancy modes (e.g., between first and second modes) with $x-$core. The red circles and lines indicate the linear transformation $T'(\theta,\varphi)$ act on the adjacent modes (e.g., between first and eleventh modes) with $y-$core. More QPU cores can be appended in the similar way for further scaling the GBS machine.

θ_1 = 3.93356	θ_{12} = 4.75644	φ_1 = 2.65679	φ_{12} = 2.22716
θ_2 = 0.585623	θ_{13} = 3.55327	φ_2 = 3.95457	φ_{13} = 3.01646
θ_3 = 1.43303	θ_{14} = 2.41713	φ_3 = 1.95002	φ_{14} = 3.44137
θ_4 = 2.5317	θ_{15} = 1.96771	φ_4 = 2.60377	φ_{15} = 1.24815
θ_5 = 3.52346	θ_{16} = 1.12191	φ_5 = 4.90323	φ_{16} = 2.29103
θ_6 = 1.4547	θ_{17} = 2.34792	φ_6 = 2.40476	φ_{17} = 2.0957
θ_7 = 2.29501	θ_{18} = 1.53458	φ_7 = -0.0550217	φ_{18} = 3.12359
θ_8 = 2.20983	θ_{19} = 2.90917	φ_8 = 0.940404	φ_{19} = 3.93657
θ_9 = 1.22198	θ_{20} = 2.78055	φ_9 = 2.64705	φ_{20} = 1.10818
θ_{10} = 1.90854	θ_{21} = 2.81034	φ_{10} = 3.37029	φ_{21} = 1.61904
θ_{11} = 2.23667		φ_{11} = 1.41565	

Supplementary Table 4: **Full parameters for reconstructing the unitary operation**. Expect the above parameters, a global phase compensation $\phi = 4.13373$.

can successfully construct any arbitrary unitary operation.

After discussing the expansion method for mode number and photon number, we will now discuss about reducing losses further and increasing the photon number to demonstrate even stronger quantum computational advantages. To increase the number of photons and increase computational complexity, two key aspects need to be considered. Firstly, optimizing the encoding method to reduce the circuit depth required for achieving large-scale GBS. Secondly, minimizing the loss within each cycle. In the above, we have discussed that by employing a combination of "multi-core" and "polarization-time-hybrid" encoding, the circuit depth for a 100-mode GBS task can be reduced from the current requirement of 100 cycles to only 28 cycles. This significant reduction in circuit depth has the potential to greatly decrease the total loss and improve the photon number.

Regarding the second aspect, it is an area where we are trying to make improvements. The key to reducing loss within each cycle is the use of electro-optic modulators (EOM) with low insertion loss. The EOMs we currently have from QuBIG (the same ones used in the Borealis machine [20]) demonstrate significant advantages in our time-domain GBS setup, with extremely high transmittance (measured up to approximately 99.5%). Thus, we discuss how to build a relatively low-loss scalable GBS machine using these EOMs as an example. Since the bandwidth of these EOMs is 6.7 MHz, a long fibre delay-line is required for a larger number of modes. For example, a 100-mode GBS experiment

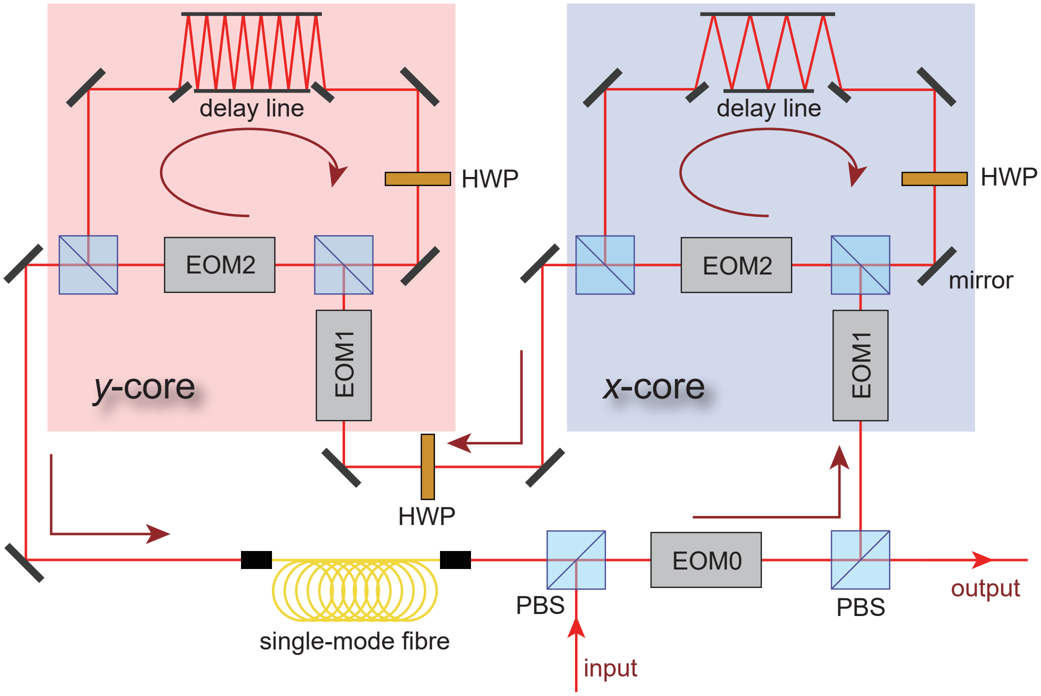

Supplementary Figure 43: **Experimental setup scheme for realizing "multi-core" encoding**. The parts covered with blue and red shadows work as $x-$core and $y-$core, respectively. EOM1 and EOM2 are used to adjust phase and polarization in each core, respectively. The length of the delay-line is different in each core. EOM0 is used to control the time-bin sequences that are still evolved in the loop or detected by SNSPD.

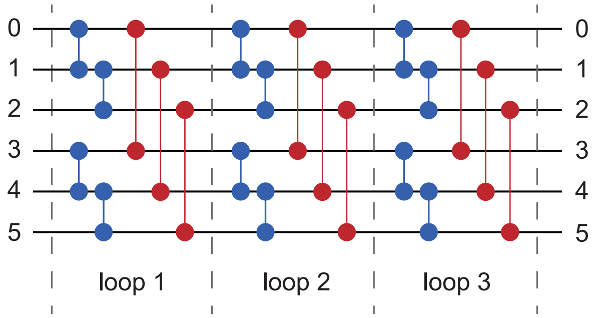

Supplementary Figure 44: **Six-mode multidimensional encoding circuit**.

would necessitate a fibre delay line of approximately 3,000 meters, resulting in an additional 12% loss. To mitigate this significant loss, a direct approach is to introduce a free-space optical storage device.

By incorporating a modified Herriott cell structure, as described in a recent work by N. T. Arnold et al. [52], and extending the length of the cell to a relatively long scale (e.g., 6 meters) while maintaining a vacuum environment, along with applying high-reflection coatings to achieve a reflectivity of 0.9999 on the mirrors for the signal light, we can achieve a path length of 3,000 meters with minimal loss. Specifically, a 3,000 m delay line requires a total of 500 reflections within the cell, and then the loss is $1 - 0.9999^{500} = 0.049$.

While this direct method of using a very long cell for free space optical storage is straightforward in concept, practical implementation poses challenges due to limitations in experimental space and other factors. These difficulties need to be taken into consideration when considering the direct approach of a free space optical storage device.

An alternative and effective approach is to employ a "distributed" computing method. In this approach, we utilize the concept of "distributed quantum computing" by introducing additional spatial dimensions to scale the GBS machine. Multiple individual dual-core nodes are utilized to perform separate unitary operations on the same table

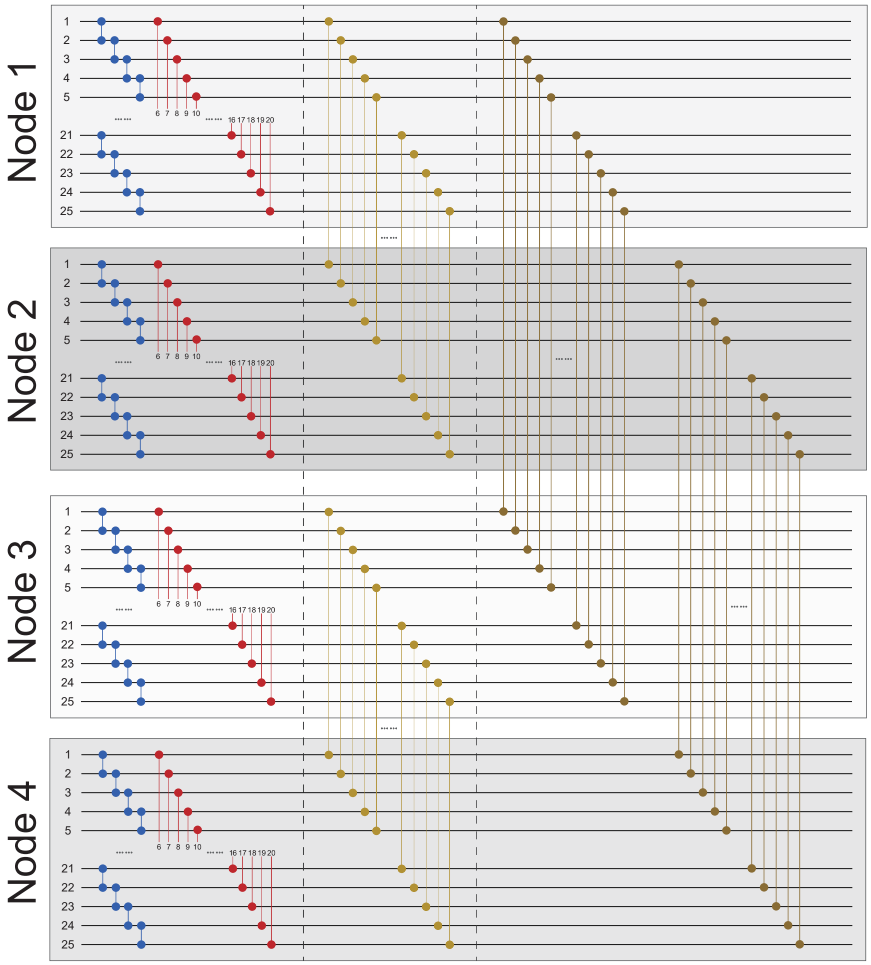

Supplementary Figure 45: **Circuits about the "distribute" scheme**. The blue and red connections denote the local SU2 operations, and the light (or dark) yellow connections denote the operations between each node.

and are then interconnected. The figures below depict a one-loop circuit for a 100-mode Gaussian boson sampling (GBS) task and the corresponding experimental setup. Unlike the scenario shown in Supplementary Figure 42, this "distributed" circuit configuration illustrates that the 100-mode GBS task is achieved by combining four independent quantum processing units (QPUs) instead of a single QPU. Each independent QPU consists of 25 modes, resulting in a reduction of three-quarters in the length of fibre delay lines compared to a single QPU performing the 100-mode GBS task, requiring only 750 meters. The blue and red lines represent local operations, while the light (or dark) yellow lines represent spatial interference between each node.

This reduction significantly reduces losses associated with long fibre delay lines. The circuit shown in Supplementary Figure 45 illustrates the process of achieving the 100-mode GBS task using a "distributed" computing approach. Furthermore, the universality of the entire device can still be preserved through iterative operations which is depicted in the diagram above. By employing the same approach as previously described, we can calculate that in order to maintain universality and programmability, this circuit needs to be iterated for 20 cycles, which is equivalent to $\lceil 100^2/((160+50+50)\times 2)\rceil = 20$ (in each loop, there are totally 160 local SU2 operations (denoted as red and blue lines), and 50 SU2 operations between each node (denoted as light and dark yellow lines, respectively), and each operation contains two free parameters, θ and φ).

Based on the transmittance analysis values of each component (as shown in Supplementary Table 5):

The loss per cycle is calculated as $0.995^8 \times 0.995 \times 0.989 \times 0.97 \times 0.969 \times 0.995 \times 0.990 \approx 0.880$, resulting in a total loss of $0.880^{20} \times 0.90$ (coupling efficiency from ppKTP to fibre)$\times 0.80$ (input by AOM)$\times 0.80$ (output by AOM)$\times 0.97$ (coupling efficiency from QPU to fibre)$\times 0.973$ (transmission of demultiplexer)$\times 0.95$ (detection efficiency of SNSPDs)≈ 0.0357. If the squeezing level of light sources in per time-bin is 2.2, the average number of photons can

Experimental component	Description	Number in per loop	Transmission
EOM	QuBIG	8	≈ 0.995
45m delay line	Herriott cell (1m-cell)	1	≈ 0.995
225m delay line	Herriott cell (2m-cell)	1	≈ 0.989
coupling from free space to single mode fibre	Graded-Index (GRIN) lens coupler	1	≈ 0.970
750m long single mode fibre	SMF-28 Ultra	1	≈ 0.969
AOM	transmission efficiency	1	≈ 0.995
Other components like mirrors and wave plates	all coated with high-transmission film	≈ 20	≈ 0.990

Supplementary Table 5: **Approximate transmittance about each component**.

be increased to 70. This would be highly advantageous for achieving quantum computational advantages, and this "distributed" quantum computing approach enables scalability in our time-bin system, ensuring that the overall loss of the GBS machine remains relatively low even at higher modes.

In that case, our GBS machine would be capable of solving the clique-finding problem for a graph with 100 vertices. In this scenario, we can detect approximately 50 clicks (not in the collision-free space), meaning that a subgraph with around 50 vertices that can be detected (by employing a similar scale of squeezing level, as illustrated in Supplementary Figure 26(a) or Supplementary Figure 26(b)). This situation may can be applied to measure the similarity of graphs [53]. However, many other graph problems require operating in a collision-free space (like the clique-finding task introduced in our work). This condition can be achieved by reducing the squeezing level on each time-bin mode. We have observed that when the output average photon number is reduced to around 20, there are virtually no collisions between two photons. Therefore, we can expect to detect cliques with 20 vertices in a collision-free space with a probability of approximately 7.3% (i.e., in one second, there are around 1460 20-vertice-clique sample events can be detected).

We want to emphasis that this universality feature is also quite important to exhibit the quantum computational advantage. In our present experiments, not only the loss hinders the demonstration of quantum computational advantage, but also the sampling matrix here is non-negative (i.e., the adjacency matrix of graph), and this feature will also reduce the difficulty of classical simulation. Although the purpose of this work is not to show quantum computational advantage, because our setup is universal programmable, it can be sample from any arbitrary complex sampling matrix, which is classically hard one. Therefore, this improved *Abacus* can potentially exhibit quantum computational advantage on more general clique decision problems.

Using the architecture shown in Supplementary Figure 45, we can realize a large-scale, low-loss GBS machine, and can achieve quantum computational advantages.

The loss estimate here may seem high, but the system still retains non-classicality despite this loss. This can be verified by violating the inequality in Ref. [54]:

$$\operatorname{sech}\Big\{\frac{1}{2}\Theta\Big[\ln\Big(\frac{1-2q_d}{\eta e^{-2r}+1-\eta}\Big)\Big]\Big\} > e^{-\epsilon^2/4K}. \tag{18}$$

Here, r is the squeezing parameter of the photon sources; η is the overall transmission rate; $q_D = p_D/\eta_D$, where p_D is the dark count rate and η_D is the quantum efficiency; and Θ is the ramp function $\Theta(x) = \max(x, 0)$. Violation of this inequality means the GBS experiment cannot be approximated by classical states with non-negative phase-space quasi-probability distributions, up to total variation distance epsilon. According to the above analysis, if we send 100 squeezed vacuumed state (i.e., $K = 100$) with $r = 2.40$ to the "multi-core"-"distributed"-improved 100-mode *Abacus*, and $\eta = 0.025$ ($\eta_D = 0.95$, and $p_D = 10^{-4}$ can be obtained from empirical parameters), we can find this inequality is still violated for error up to 17%. We want to note here that this experiment design is not in the collisionless regime anymore. But even if a non-negligible number of outcomes are collisional, we still expect this device to attain quantum advantage over existing classical devices. Since there are no present simulation algorithms able to exploit the repeated entries for speedup, and in fact, state-of-the-art GBS algorithm [55] perform worse in the presence of collision.

Finally, we will estimate the time for calculating the probability distribution of a pure-state GBS experiment with the best-known algorithm [20]:

$$\text{time}(N) = \frac{1}{2}cMN^3G^{N/2}, \tag{19}$$

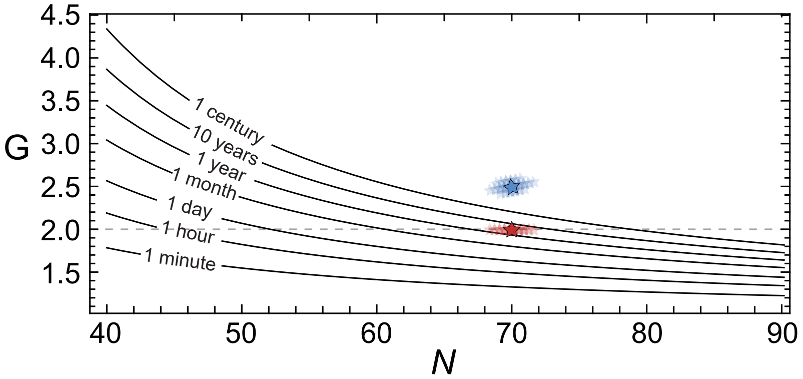

Supplementary Figure 46: **Diagram about classical simulation time**. The time required for calculating 10^7 100-mode Gaussian boson sampling results with supercomputer *Frontier*. The red stars denote the classical simulation time in the case of $N \approx 70$ when threshold detection is used, and the blue stars denote the classical simulation time corresponds to the condition under which the photon-number-resolved detection is used in GBS experiment.

where the collision parameter G is always equal to 2 since the experiment is performed in the collision-free region with threshold detectors, and N is the total number of detectors that detect photon. As for the fastest supercomputer at present, the *Frontier* is 2.5 times faster than *Fugaku* (estimate the data from LINPACK), and $c_{\text{Frontier}} = c_{\text{Fugaku}}/2.5 = 1.764^{-17}$s. If using the above introduced 100-mode GBS machine as an example, then $M = 100$, and N can reach approximately 70 with a relative high probability when we set all the squeezing parameters r as 2.4. It can be easily achieved since the squeezing parameter r can already reach around 2.9 using ppKTP waveguide based on the existing techniques [7]. In this case, we can use Supplementary Equation 19 to estimate that it will take *Frontier* near several years to calculate 10^7 sampling data. Besides, if photon number resolving detection is considered in experiments, it takes about 500 years for the same amount of data to be simulated by *Frontier*, as shown in Supplementary Figure 46. At the same time, as for the above introduced improved *Abacus*, we can estimate the probability to obtain a 120-photon event (corresponding to $N \approx 70$) is approximately 0.02 [55] under the total transmission $\eta = 0.03$. Besides, the sampling repetition rate can be set as 4kHz, since 100/2(number of modes/2)×150ns(using 6.7MHz bandwidth)×32(number of loop)=240μs (namely, approximate 4kHz). Thus, the improved *Abacus* only need approximate 42 minutes to obtain these 10^7 sampling data.

Lastly, we want to highlight here that our architecture, when compared to current photonic quantum processors, holds great promise as a leading candidate for real-world applications and commercialization based on the current level of technology. This makes it possible for the integration of quantum computing and its computational advantages into actual drug design industries in the near future.

IX. FURTHER DISCUSSION ABOUT CLASSICAL COMBINATORIAL OPTIMIZATION METHODS

A. Comparison with Genetic Algorithms

Quantum computing-based clique-finding algorithms, such as those using GBS, have the potential to offer advantages over classical combinatorial optimization methods like genetic algorithms [56]. However, like we discussed before, the field of quantum computing is still in its infancy. Its practical application and comparison with classical computing software still have limitations.

Genetic algorithms and other classical methods typically represent the problem using bit strings or other discrete representations. In contrast, quantum algorithms like GBS can naturally represent the problem using continuous variables. This difference in representation can lead to different approaches for problem modelling and optimization.

Genetic algorithms use evolutionary search techniques, including creating and evolving a set of potential solutions. This kind of algorithm relies on various mechanisms such as mutation, crossover, and selection to explore the solution space. However, quantum algorithms use the quantum resources like coherence or entanglement to find the solutions. Quantum algorithms can leverage quantum interference effects to enhance or suppress certain solutions, potentially leading to more efficient exploration of the solution space for specific problems.

One of the potential advantages of quantum computing is the ability to solve certain problems faster than classical

methods, especially for problems that are computationally hard. However, the actual speedup of quantum algorithms depends on several factors, including the size and complexity of the problem [57], the scalability of the quantum hardware, and the algorithm design (as we discussed above). At present, the scale of all quantum computing is still limited, so at this stage, using quantum computer to efficiently solve large-scale practical problems is still a continuing research content.

The choice between quantum and classical methods for clique-finding or any combinatorial optimization problem depends on the specific problem characteristics, available resources, and performance requirements. Classical methods like genetic algorithms have been extensively studied and optimized for various problem domains. They may be more suitable when dealing with smaller problem instances or problems that can be efficiently solved classically. Meanwhile, quantum device may provide computational advantages for large-scale problems that are difficult to solve using classical methods. Furthermore, using the quantum-classical hybrid methods to solve the large-scale problems is also an interesting area in the future.

It's worth to note that the comparison between quantum methods and classical methods is an active research field. At present, more efficient classical algorithms are constantly challenging the quantum computational advantages, while the performance of quantum algorithms and quantum devices is also still being explored and improved. As quantum technologies advance and larger, more fault-tolerant quantum computers become available, a clearer understanding of the relative strengths and limitations of quantum computing for combinatorial optimization problems like clique-finding will emerge.

B. Comparisons with other quantum or quantum-inspired approaches

GBS is a specific quantum computing approach that focuses on sampling from the output distribution of a linear-optical circuit. While GBS shows promise for applications like simulating boson sampling circuits, it is important to distinguish it from other quantum or quantum-inspired approaches for combinatorial optimization. Here, we will analyze and discuss several commonly used quantum or quantum-inspired approaches.

Quantum Approximate Optimization Algorithm (QAOA) is a popular approach in combinatorial optimization methods. QAOA utilizes a sequence of quantum gates to evolve a quantum state that encodes potential solutions to the optimization problem. Extensive research has been conducted on QAOA, demonstrating promising results for specific optimization problems, including graph problems such as maximum clique and MaxCut problems [58, 59]. QAOA employs a quantum circuit with problem-specific and driver gates to encode and drive the quantum state towards optimal solutions. Due to its ability to search the solution space in a parallel manner, QAOA may provide speedups than classical algorithms. Although QAOA has been proven to improve the solution quality with increasing depth, scaling this approach on real NISQ quantum hardware is challenging due to errors caused by qubit imperfections, such as cross-talk or decoherence, as well as errors resulting from faulty gates and measurements. If we aim to process a graph with 32 nodes in our work, the QAOA method typically requires a 32-qubit programmable quantum computer. However, achieving the necessary hardware conditions is currently difficult. Recent references suggest that a significant quantum advantage is likely to be observed on real quantum hardware once the noise decreases by two orders of magnitude [60]. Consequently, researchers are constrained to running QAOA experiments only on small circuits [61].

Quantum annealing is another method that utilizes quantum effects to solve combinatorial optimization problems. It entails encoding the problem into a quantum system's ground state and gradually evolving the system towards that state using specialized hardware like D-Wave's quantum annealers. One specific application of quantum annealing is solving graph problems, such as clique-finding [62–64]. The process follows the principle of adiabatic quantum computation, where the quantum system evolves towards the lowest energy state representing the optimal solution. Graph problems are typically formulated as Ising models, aiming to find the spin configuration that minimizes the Ising energy function. Quantum annealing hardware, such as D-Wave systems, is designed with qubits to facilitate the annealing process. Evaluating the performance of quantum annealing involves comparing obtained solutions with known optima or utilizing relevant metrics specific to the graph problem at hand. It is important to note that the effectiveness of quantum annealing for graph problems heavily depends on the problem size, structure, and the particular hardware implementation being used. While quantum annealing shows potential advantages over classical approaches for large and complex instances, it is essential to consider limitations like qubit connectivity and noise effects. Hybrid approaches that combine classical and quantum algorithms can be employed to enhance performance.

Digital annealer, on the other hand, is classical computing devices inspired by quantum annealing. It applies classical hardware to simulate the behavior of quantum annealers and aim to solve optimization problems using annealing techniques. Unlike quantum annealer, digital annealer operates on classical principles. It provides a classical approach to solving graph problems, such as clique-finding. By employing simulated annealing techniques on classical hardware, digital annealers explore the solution space and seek optimal configurations. While digital

annealers are suitable for smaller instances, scaling their capabilities to larger graphs can pose challenges. Evaluating their performance entails comparing solutions with known optima and considering factors such as solution quality and runtime. To further enhance their graph problem-solving capabilities, hybrid approaches that combine digital annealing with classical techniques can be employed.

When comparing GBS to these approaches, it is important to consider the specific problem being addressed and the available computational resources. GBS primarily focuses on sampling from the output distribution of linear-optical circuits, while QAOA, quantum annealers, and digital annealers aim to find optimal solutions to combinatorial optimization problems The applicability of each method depends on various factors, such as different types of problems, available hardware, and specific requirements for optimization tasks.

---

[1] Hamilton, C. S., Kruse, R., Sansoni, L., Barkhofen, S., Silberhorn, C. & Jex, I. Gaussian boson sampling. *Phys. Rev. Lett.* **119**, 170501 (2017).

[2] Kruse, R., Hamilton, C. S., Sansoni, L., Barkhofen, S., Silberhorn, C. & Jex, I. Detailed study of Gaussian boson sampling. *Phys. Rev. A* **100**, 032326 (2019).

[3] Spring, J. B. et. al. Boson sampling on a photonic chip, *Science* **339**, 798-801 (2012).

[4] Tillmann, M., Dakić, B., Heilmann, R., Nolte, S., Szameit, A. & Walther, P. Experimental boson sampling, *Nature Photon.* **7**, 540-544 (2013).

[5] Brádler, K., Dallaire-Demers, P.-L., Rebentrost, P., Su, D. & Weedbrook, C. Gaussian boson sampling for perfect matchings of arbitrary graphs. *Phys. Rev. A* **98**, 032310 (2018).

[6] Jahangiri, S., Arrazola, J. M., Quesada, N., & Killoran, N. Pointer processes with Gaussian boson sampling. *Phys. Rev. E* **101**, 022134 (2020).

[7] Harder, G., Bartley, T. J., Lita, A. E., Nam, S. W., Gerrits, T., & Silberhorn, C. Single-mode parametric-down-conversion states with 50 photons as a source for mesoscopic quantum optics. *Phys. Rev. Lett.* **116**, 143601 (2016).

[8] Taballione, C. et. al. 8×8 reconfigurable quantum photonic processor based on silicon nitride waveguides. *Optics Express* **27**, 26842 (2019).

[9] Eckstein, A., Christ, A., Mosley, P. J., & Silberhorn, C. Highly efficient single-pass source of pulsed single-mode twin beams of light. *Phys. Rev. Lett.* **106**, 013603 (2011).

[10] Bell, B. A., Thekkadath, G. S., Ge, R., Cai, X., & Walmsley, I. A., Testing multi-photon interference on a silicon chip. *Optics Express* **27**, 35646 (2019).

[11] Lvovsky, A. I., Squeezed light. arXiv:1401.4118 (2016).

[12] Sempere-Llagostera, S., Patel, R. B., Walmsley, I. A., & Kolthammer, W. S., Experimentally finding dense subgraphs using a time-bin encoded Gaussian boson sampling device. *Phys. Rev. X* **12**, 031045 (2022).

[13] Clements, W. R., Humphreys, P. C., Metcalf, B. J., Kolthammer, W. S., & Walmsley, I. A. Optimal design for universal multiport interferometers. *Optica* **12**, 1460-1465 (2016).

[14] Motes, K. R., Dowling, J. P., Gilchrist, A. & Rohde, P. P., Implementing boson sampling with time-bin encoding: Analysis of loss, mode mismatch, and time jitter. *Phys. Rev. A* **92**, 052319 (2015).

[15] Killoran, N. et. al. Strawberry Fields: A software platform for photonic quantum computing. *Quantum* **3**, 129 (2019).

[16] Zhong, H.-S. et. al. Quantum computational advantage using photons. *Science* **370**, 1460-1463 (2020).

[17] Arrazola, J. M. et. al. Quantum circuits with many photons on a programmable nanophotonic chip. *Nature* **591**, 54-60 (2021).

[18] Thekkadath, G. S., Sempere-Llagostera, S., Bell, B. A., Patel, R. B., Kim, M. S., & Walmsley, I. A., Experimental demonstration of Gaussian boson sampling with displacement. *Phys. Rev. X Quantum* **3**, 020336 (2022).

[19] Spagnolo, N., et al. Experimental validation of photonic boson sampling. *Nature Photonics* **8**, 615-620 (2015).

[20] Madsen, L. S. et. al. Quantum computational advantage with programmable photonic processor. *Nature* **606**, 75-81 (2022).

[21] Hansen, L. M. et. al. Single-active-element demultiplexed multi-photon source. arXiv: 2304.12956 (2023).

[22] Kharkar, P. S., Warrier, S., & Gaud, R. S., Reverse docking: a powerful tool for drug repositioning and drug rescue. *Future medicinal chemistry* **6**, 333-342 (2014).

[23] Bhattacharjee, B., & Chatterjee, J., Identification of proapoptopic, anti-inflammatory, anti-proliferative, anti-invasive and anti-angiogenic targets of essential oils in cardamom by dual reverse virtual screening and binding pose analysis. *Asian Pacific Journal of Cancer Prevention* **14** 3735-3742 (2013).

[24] Carvalho, D. et. al. Structural evidence of quercetin multi-target bioactivity: A reverse virtual screening strategy. *European Journal of pharmaceutical sciences* **106** 393-403 (2017).

[25] Banchi, L., Fingerhuth, M., Babej. T., Ing, C., & Arrazola, J. M., Molecular docking with Gaussian boson sampling. *Sicence Advances* **6**, eaax1950 (2020).

[26] Dias, R., & Azevedo Jr, W. F. de, Molecular docking algorithms, *Curr. Drug Targets* **9**, 1040-1047 (2008).

[27] Kuhl, F. S., Crippen, G. M., & Friesen, D. K., A combinatorial algorithm for calculating ligand binding. *J. Comput. Chem.* **5**, 24-34 (1984).

[28] Rognan, D. Structure-based approaches to target fishing and ligand profiling. *Molecular Information* **29**, 176-187 (2010).

[29] Koutsoukas, A. et. al. From in silico target prediction to multi-target drug design: current database, methods, and application. *Journal of Proteomics* **74**, 2554-2574 (2011).

[30] Levin, J. I., et. al. Acetylenic TACE inhibitors. Part 3: Thiomorpholine sulfonamide hydroxamates, *Bioorganic & medicinal chemistry letters* **16**, 1605-1609 (2006).
[31] DesJarlais, R. L., Sheridan, R. P., Dixon, J. S., Kuntz, I. D., Venkataraghavan, R., Docking flexible ligands to macromolecular receptors by molecular shape. *J. Med. Chem.* **29**, 2149¨C2153 (1986).
[32] Johannes, J. W., et. al. Discovery of 5-4-[(7-Ethyl-6-oxo-5, 6-dihydro-1, 5-naphthyridin-3-yl) methyl] piperazin-1-yl-N-methylpyridine-2-carboxamide (AZD5305): A PARP1-DNA Trapper with High Selectivity for PARP1 over PARP2 and Other PARPs, *Journal of Medicinal Chemistry* **64**, 14498-14512 (2021).
[33] Wang, R., X. Fang, X., Lu, Y., & Wang, S., The PDBbind database: Collection of binding affinities for protein-ligand complexes with known three-dimensional structures. *J. Med. Chem.* **47**, 2977-2980 (2004).
[34] Wang, R., X. Fang, X., Lu, Y., Yang, C.-Y., & Wang, S., The PDBbind database: Methodologies and updates. *J. Med. Chem.* **48**, 4111-4119 (2005).
[35] Liu, Z. et.al. PDB-wide collection of binding data: Current status of the PDBbind database. *Bioinformatics* **31**, 405-412 (2014).
[36] Amé, J., Spenlehauer, C., & Murcia, G. de, The PARP superfamily, *Bioessays* **26**, 882-893 (2004).
[37] Rose, M., et. al. PARP inhibitors: clinical relevance, mechanisms of action and tumor resistance, *Frontiers in Cell and Developmental Biology* **8**, 564601 (2020).
[38] Powell, C., et. al. Pre-clinical and clinical evaluation of PARP inhibitors as tumour-specific radiosensitisers, *Cancer treatment reviews* **36**, 566-575 (2010).
[39] Lu, Y., et. al. Double-barreled gun: Combination of PARP inhibitor with conventional chemotherapy, *Pharmacology & therapeutics* **188**, 168-175 (2018).
[40] Tangutoori, S., Baldwin, P., & Sridhar, S. PARP inhibitors: A new era of targeted therapy, *Maturitas* **81**, 5-9 (2015).
[41] Lord, C. J., & Ashworth, A. PARP inhibitors: Synthetic lethality in the clinic, *Science* **355**, 1152-1158 (2017).
[42] Olsen, A. L., & Feany, M. B., PARP inhibitors and Parkinson's disease, *New England Journal of Medicine* **380**, 492-494 (2019).
[43] Martire, S., Mosca, L., & d'Erme, M., PARP-1 involvement in neurodegeneration: a focus on Alzheimer's and Parkinson's diseases, *Mechanisms of ageing and development* **146**, 53-64 (2015).
[44] Canan, S. S., Structure Based Design of PARP Inhibitors, *PARP Inhibitors for Cancer Therapy* Humana Press, Cham, 205-221 (2015).
[45] Martin, T. W., and Derewenda. Z. S. The name is bond-H bond. *Nature structural biology* **6**, 403-406 (1999).
[46] Tao, C., Li, M., & Liu. J. $\Pi - \Pi$ stacking interaction: a nondestructive and facile means in material engineering for bioapplications. *Crystal Growth & Design* **18**, 2765-2783 (2018).
[47] Tang, M., Hwang, k., & Kang, S. H. StemP: A fast and deterministic Stem-graph approach for RNA secondary structure prediction. IEEE/ACM Transactions on Computational Biology and Bioinformatics 1-14 (2023).
[48] Feingold, J., Bellofatto, V., Shapiro, L., & Amemiya, K. Organization and nucleotide sequence analysis of an rRNA and tRNA gene cluster from *Caulobacter crescentus*, *Journal of Bacteriology* **163**, 155-166 (1985).
[49] Maeda, T., Takada, N., Furushita, M. and Shiba, T., Structural variation in the 16S-23S rRNA intergenic spacers of Vibrio parahaemolyticus, *FEMS Microbiol. Lett.* **192**, 73-77 (2000).
[50] Bulmer, J. F. F., et al. The boundary for quantum advantage in Gaussian boson sampling, *Sicence Advances* **8**, eabl9236 (2022).
[51] Enomoto, Y., Yonezu, K., Mitsuhashi, Y., Takase, K., & Takeda, S., Programmable and sequential Gaussian gates in a loop-based single-mode photonic quantum processor, *Sci. Adv.* **7**, eabj6624 (2021).
[52] Arnold, N. T., Victora, M., Goggin, M. E., & Kwiat, P. G., Free-space photonic quantum memory, Proc. SPIE 12446, *Quantum Computing, Communication, and Simulation III*, **12446**, 1244606, (2023).
[53] Schuld, M., Bradler, K., Israel, R., Su, D., & Gupt, B., Measuring the similarity of graphs with a Gaussian boson sampler, *Phys. Rev. A* **101**, 032314 (2020)]
[54] Qi, H.,Brod, D. J.,Quesada, N., & Garcia-Patron R., Regimes of Classical Simulability for Noisy Gaussian Boson Sampling. *Phys. Rev. Lett.* **124**, 100502 (2020).
[55] Deshpande, A. et. al. Quantum computational advantage via high-dimensional Gaussian boson sampling. *Science Advances* **8**, eabi7894 (2022).
[56] Acampora, G., Chiatto, A., & Vitiello, A., Genetic algorithms as classical optimizer for the Quantum Approximate Optimization Algorithm, *Applied Soft Computing* **142**, 110296 (2023).
[57] [Oh, C., Lim, Y., Wong, Y., Fefferman, B., & Jiang, L., Quantum-inspired classical algorithm for molecular vibronic spectra, arXiv: 2202.01861 (2023).
[58] Zhou, Z., Du, Y., Tian, X., & Tao, D., QAOA-in-QAOA: Solving large-scale MaxCut problems on small quantum machines, *Phys. Rev. Applied* **19**, 024027 (2023).
[59] Herrman R. et. al., Impact of graph structures for QAOA on MaxCut, *Quantum Information Processing* **20**, 289 (2021).
[60] Franca, S., & Garcia-Patron, R. *Nat. Physics* **17**, 1221¨C1227 (2021).
[61] Kurowski, K. et. al., *European Journal of Operational Research* **310**, 518¨C528 (2023).
[62] Chapuis, G., Djidjev, H., Hahn, G., & Rizk, G., Finding maximum cliques on a quantum annealer, *Proceedings of the Computing Frontiers Conference* 63-70 (2017).
[63] Djidjev, H. N., Chapuis, G., Hahn, G., & Rizk, G., Efficient combinatorial optimization using quantum annealing, arXiv:1801.08653 (2018).
[64] Metwalli, S. A., Gall, F. L., & Meter, R. V., Finding small and large k-clique instances on a quantum computer, *IEEE Transactions on Quantum Engineering* **1**, 3102911 (2020).
